# BCAT2 Shapes a Noninflamed Tumor Microenvironment and Induces Resistance to Anti‐PD‐1/PD‐L1 Immunotherapy by Negatively Regulating Proinflammatory Chemokines and Anticancer Immunity

**DOI:** 10.1002/advs.202207155

**Published:** 2023-01-15

**Authors:** Zhiyong Cai, Jinbo Chen, Zhengzheng Yu, Huihuang Li, Zhi Liu, Dingshan Deng, Jinhui Liu, Chunliang Chen, Chunyu Zhang, Zhenyu Ou, Minfeng Chen, Jiao Hu, Xiongbing Zu

**Affiliations:** ^1^ Department of Urology Xiangya Hospital Central South University Changsha Hunan 410008 P. R. China; ^2^ National Clinical Research Center for Geriatric Disorders Xiangya Hospital Central South University Changsha Hunan 410008 P. R. China; ^3^ Research Center of Carcinogenesis and Targeted Therapy Xiangya Hospital Central South University Changsha Hunan 410008 P. R. China

**Keywords:** immunotherapy, molecular subtype, precision therapy, tumor microenvironment

## Abstract

To improve response rate of monotherapy of immune checkpoint blockade (ICB), it is necessary to find an emerging target in combination therapy. Through analyzing tumor microenvironment (TME)‐related indicators, it is validated that BCAT2 shapes a noninflamed TME in bladder cancer. The outcomes of multiomics indicate that BCAT2 has an inhibitory effect on cytotoxic lymphocyte recruitment by restraining activities of proinflammatory cytokine/chemokine‐related pathways and T‐cell‐chemotaxis pathway. Immunoassays reveal that secretion of CD8^+^T‐cell‐related chemokines keeps a robust negative correlation with BCAT2, generating a decreasing tendency of CD8^+^T cells around BCAT2^+^ tumor cells from far to near. Cotreatment of BCAT2 deficiency and anti‐PD‐1 antibody has a synergistic effect in vivo, implying the potential of BCAT2 in combination therapy. Moreover, the value of BCAT2 in predicting efficacy of immunotherapy is validated in multiple immunotherapy cohorts. Together, as a key molecule in TME, BCAT2 is an emerging target in combination with ICB and a biomarker of guiding precision therapy.

## Graphical abstract

By multiomics analysis, experiment validation and cohort comparison, it is found that BCAT2 is a potential combination therapy target in immunotherapy. Inhibition of BCAT2 can transform a noninflamed tumor microenvironment (TME) to an inflamed TME by regulating secretion levels of CD8^+^T‐cell‐related chemokines. Furthermore, it is also a potential predictive biomarker of efficacy of immunotherapy.

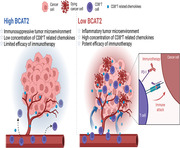

## 1 Introduction

Bladder cancer (BLCA) is one of the most common malignancies in the urinary system.^[^ ^1^ ^]^ It is estimated that over 430 000 patients diagnosed worldwide every year.^[^ ^2^ ^]^ In spite of radical surgery treatment, almost a half of patients with muscle‐invasive bladder cancer occur metastasis.^[^ ^3^ ^]^ Hence, systemic therapy plays an important role in advanced bladder cancer. With the development of immunotherapy, especially immune checkpoint blockade (ICB), accumulating evidences indicated that ICB has an excellent performance on eliminating tumor burden.^[^ ^4^ ^]^ However, there are still a large number of patients failed to respond to monotherapy of ICB by reason of primary resistance or acquired resistance.^[^ ^5^ ^]^ For resolving the unmet clinical need, combination other rational therapy with ICB provides a brand‐new insight into monotherapy resistance.

Tumor microenvironment (TME) is a complicated system and has a profound impact on efficacy of immunotherapy.^[^ ^6^ ^]^ With insufficient infiltration level of cytotoxic T lymphocytes (CTLs), noninflamed TME was considered as a critical factor in failing to generate potent antitumor immune response during immunotherapy.^[^ ^7^ ^]^ Therefore, it is vital to find a key molecule which can remodel noninflamed TME into inflamed TME and has potential to be a combination therapy target.

Branched chain aminotransferase 2 (BCAT2) is a core enzyme in the process of sulfur amino acid metabolism.^[^ ^8^ ^]^ Li et al. found that BCAT2 was essential for development of pancreatic cancer by mediating branched‐chain amino acids (BCAAs) catabolism.^[^ ^9^ ^]^ Lee et al. demonstrated that BCAT2 deficiency inhibited tumor growth of pancreatic ductal adenocarcinoma (PDAC) by regulating lipid metabolism.^[^ ^10^ ^]^ Although there were several researches documented that BCAT2 directly influenced the biological process of tumor by regulating metabolic related pathways, its role in regulating TME immune status has never been explored.

In our study, through comprehensive analysis of TME‐related indicators in Xiangya BLCA cohort and multiple public BLCA cohorts, we screened that BCAT2 probably shapes an immunosuppressive TME in BLCA. For exploring molecular mechanisms, we further performed single‐cell RNA sequencing (scRNA‐seq) and bulk‐RNA seq, revealed that BCAT2 acts a repressive part in recruitment CTLs into TME, by inhibiting activities of cytokine/chemokine‐associated signal pathways and T‐cell‐chemotaxis signal pathways. In vitro, multi‐immunoassays demonstrated that secretion of CTL‐related chemokines has stable negative correlations with expression of BCAT2. According to panoramic analysis of tissue microarray (TMA), we found a mutual exclusive relationship between CTLs and BCAT2^+^tumor cells in spatial distribution. In vivo, a cooperative effect was revealed in combination therapy of BCAT2 loss and ICB. More importantly, its role in forecasting curative effect of immunotherapy was validated in Xiangya BLCA immunotherapy cohort.

## 2 Results

### 2.1 BCAT2 Negatively Correlates with Anticancer Immunity of TME in BLCA

In most of cancer types, the expression of BCAT2 is higher in cancer tissues than in normal tissues (Figure S1A, Supporting Information) and its expression pattern in BLCA was validated in Xiangya BLCA Cohort (Figure S1B, Supporting Information). Besides, BCAT2 widely expresses in various cancer cell lines (Figure S1C, Supporting Information). Further, the outcomes of comprehensive pan‐cancer analysis on chemokine system, MHCs, immunostimulators and TIICs indicated that BCAT2 has significant immunosuppressive effects in a couple of cancer types, including bladder cancer (BLCA), breast cancer (BRCA), kidney renal papillary cell carcinoma (KIRP), pancreatic adenocarcinoma (PAAD), sarcoma (SARC) and thyroid carcinoma (THCA) (Figure S2A,B, Supporting Information). In addition, negative correlations between BCAT2 and common inhibitory immune checkpoints (PD‐1, PD‐L1, CTLA‐4, and LAG‐3) were also found (Figure S2C–F, Supporting Information).

**Figure 1:**
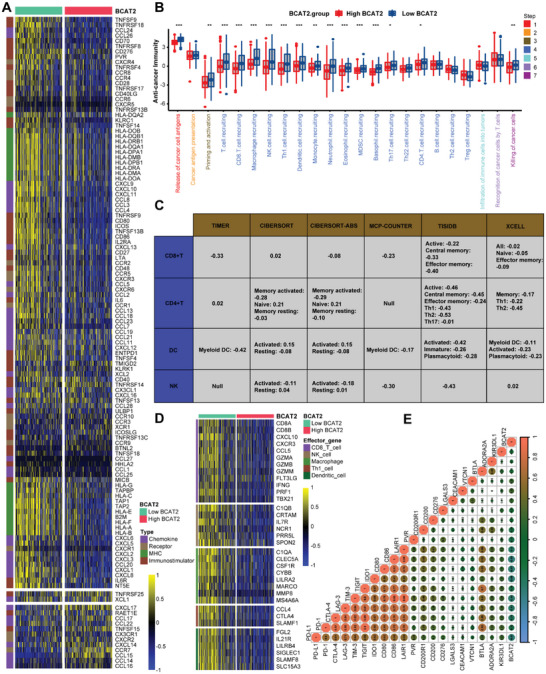
BCAT2 negatively correlates with anticancer immunity in TME of BLCA. A) Expression pattern of chemokines, chemokine receptors, MHC molecules, and immunostimulators in high and low BCAT2 groups. B) Activity of cancer immunity cycle in high and low BCAT2 groups. Seven colors represent seven steps in the cycle. *p < 0.05; **p < 0.01; ***p < 0.001. C) Correlations between BCAT2 and multiple types of immune cell (CD8^+^T cell, CD4^+^T cell, DC cell, and NK cell) in six independent algorithms. D) Expression patterns of multiple subtypes of immune cell (CD8^+^T cell, NK cell, Macrophage, Th1 cell, and DC cell) related effector genes in high and low BCAT2 groups. E) Correlations between BCAT2 and ICB related effector genes. Number in the circle means correlation coefficient.

Through comprehensive pan‐cancer analysis, we found that BCAT2 has the most profound immunosuppressive effect in BLCA. Hence, we further explored its immunological role in BLCA. On the basis of median expression value of BCAT2, we divided individuals into high‐BCAT2 group and low‐BCAT2 group in TCGA‐BLCA cohort. Obviously, a series of chemokines, chemokine receptors, MHC related molecules and immunostimulators were down‐expressed in high‐BCAT2 group and over‐expressed in low‐BCAT2 group (**Figure** **1**A). Similar outcome was found in cancer immunity cycle, which covers multiple essential steps in recruiting and infiltrating of immune cells (Figure 1B). Moreover, we demonstrated robust negative connections between infiltration level of immune cell (CD8^+^T cell, CD4^+^T cell, nature killer (NK) cell and dendritic (DC) cell) and BCAT2 in different algorithms (Figure 1C). Likewise, lower expression of immune cell's effector genes (CD8^+^T cell, NK cell, Macrophage, T helper 1 (Th1) cell and DC cell) were found in high‐BCAT2 group comparing to low‐BCAT2 group (Figure 1D). More importantly, when correlated BCAT2 with TIS score (Figure S3, Supporting Information) and inhibitory immune checkpoints (Figure 1E), obvious negative relationships were revealed between them. Hence, we speculated that BCAT2 may negatively regulates the TME immune response and affects efficacy of immunotherapy profoundly. Furthermore, above results were well validated in nine independent BLCA cohorts (GSE31684, GSE32894, GSE48075, GSE48276, GSE69795, GSE83586, GSE86411, GSE87304, and GSE128702) (Figures S4–S12, Supporting Information).

**Figure 2:**
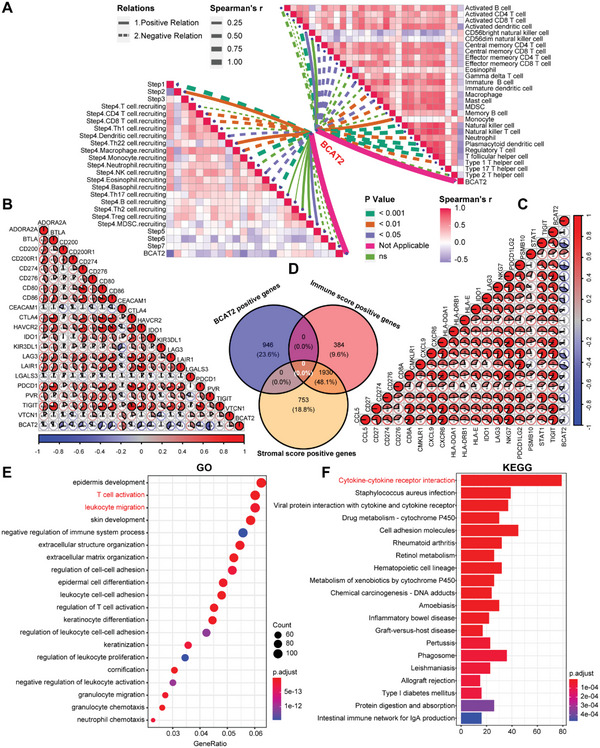
Validation the function of BCAT2 by Xiangya BLCA cohort. A) Correlations between BCAT2/cancer immunity cycle (left) and BCAT2/TIICs (right). Solid and dotted lines mean positive and negative relationships, thickness of the lines mean coefficient of correlations, lines with different colors represent *p*‐value of correlations. B) Correlations between BCAT2 and ICB related effector genes. C) Correlations between BCAT2 and TIS score related effector genes. Number in the circle represents correlation coefficient. D) Intersection of BCAT2, immune score and stromal score positive‐related genes. E,F) Top 20 enrichment signaling pathways of GO and KEGG analyses in the BCAT2‐related genes.

At last, we verified the association between BCAT2 and TME again in Xiangya BLCA Cohort. Consistently, the expression of BCAT2 has negative relations to the activity of cancer immunity cycle, infiltration levels of TIICs, TIS score and expression levels of immune checkpoints (**Figure** **2**A–C). Collectively, we revealed that high expression of BCAT2 forms a noninflamed TME in BLCA and low expression of BCAT2 shapes an inflamed TME in BLCA.

### 2.2 Exploring the Mechanism of BCAT2 in Shaping TME by Bulk RNA‐seq and scRNA‐seq

In TCGA‐BLCA cohort, DEGs between high/low BCAT2 groups, high/low immune score groups and high/low stromal score groups were identified and took intersection (Figure S13A, Supporting Information). Interestingly, BCAT2 positive‐related DEGs had no intersection with immune and stromal score positive‐related DEGs (Figure 2D). Meanwhile, same phenomenon occurred in negative‐related DEGs among them (Figure S13B, Supporting Information). These findings indicated that BCAT2 probably negatively regulates immune related pathways. Coincidently, the results of GO and KEGG analyses revealed that BCAT2‐related DEGs were most significantly enriched in the pathways of leukocytes migration, T cell activation and cytokine–cytokine receptor interaction (Figure 2E,F). Hence, we speculated that BCAT2 shapes a noninflamed TME in BLCA by inhibiting activities of cytokine/chemokine related pathways.

**Figure 3:**
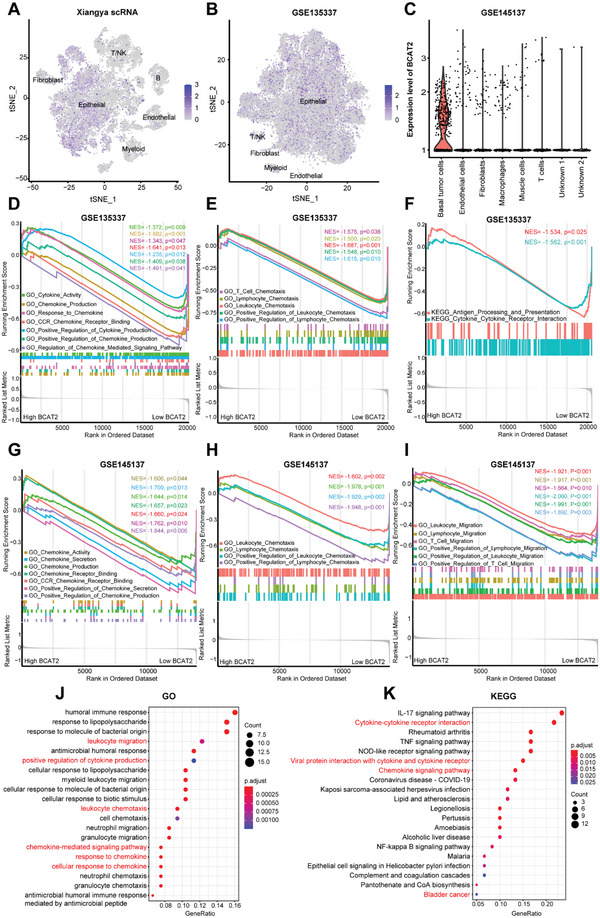
Exploring the mechanism of BCAT2 in shaping TME by bulk RNA‐seq and scRNA‐seq. A,B) tSNE plots of single‐cell expression pattern of BCAT2 in Xiangya scRNA cohort and GSE135337 cohort. C) Violin plot of single‐cell expression pattern of BCAT2 in GSE145137 cohort. D,E) GSEA analysis of GO term indicate activities of cytokine/chemokine related pathways and immune cells chemotaxis related pathways between different BCAT2 groups in GSE135337 cohort. F) GSEA analysis of KEGG term indicate activities of antigen presentation and interaction of cytokine and cytokine receptor pathway between different BCAT2 groups in GSE145137 cohort. G–I) GSEA analysis of GO term indicate activities of cytokine/chemokine related pathways, immune cells chemotaxis related pathways and immune cells migration related pathways between different BCAT2 groups in GSE145137 cohort. J,K) GO and KEGG analysis of DEGs between BCAT2 OE and BCAT2 KD cell lines.

However, the characteristic of bulk RNA sequencing determines its limitation, whose expression level of gene is calculated by mean value of all cells in tissue. To overcome the limitation, scRNA was performed on three BLCA samples. More than 19 000 cells were analyzed and classified into six categories: epithelial cell, fibroblast cell, T/NK cell, endothelial cell, B cell and myeloid cell (**Figure** **3**A), refer to recognized biomarkers (EPCAM, LYZ, CD3D, COL1A1, CD79A, CD19, PECAM1, and VWF).^[^ ^11^ ^]^ Strikingly, BCAT2 was mainly expressed in epithelial cells (EPCAM^+^), rather than endothelial cells and immune cells. On the basis of CNV accumulation, EPCAM^+^ epithelial cells were regarded as malignant urothelial cells. To validate expression pattern of BCAT2, two external scRNA cohorts were included in the study. In GSE135337 scRNA cohort, more than 36 000 cells were processed and divided into five clusters, primary expressed cell subtype of BCAT2 was still malignant bladder urothelial cell (Figure 3B). Similar expression pattern was reappeared in GSE145137 scRNA cohort (Figure 3C). Therefore, pattern of majority expression on malignant cells inferred that BCAT2 acts an immunoregulator part in TME mainly by means of varying characteristic of cancer cell.

Based on expression level of BCAT2, malignant urothelial cells were divided into high‐BCAT2 group and low‐BCAT2 group. GSEA analysis of GO terms in GSE135337 and GSE145137 scRNA cohorts revealed that a bunch of cytokine/chemokine related pathways were significantly downregulated in high‐BCAT2 group, including chemokine production, chemokine secretion, regulation of chemokine mediated signaling pathway, response to chemokine, chemokine receptor binding, cytokine activity, cytokine binding and cytokine receptor binding (Figure 3D–G; Figure S14B, Supporting Information). Meanwhile, leukocyte chemotaxis, lymphocyte chemotaxis, T cell chemotaxis and their regulatory pathways had significantly negative correlations to BCAT2 (Figure 3E–I; Figure S14A, Supporting Information). GSEA analysis of KEGG terms indicated that pathways of cytokine–cytokine receptor interaction and antigen processing and presentation were obviously downregulated in high‐BCAT2 group (Figure 3F). Together, high expression of BCAT2 represses the activities of proinflammatory cytokines and chemokines related pathways, leading to a decreased infiltration level of TIICs, which shapes a noninflamed TME ultimately.

### 2.3 BCAT2 Varies Expression Patterns of CD8^+^T‐Cell‐Related Chemokines and Inhibits Cytotoxic Capacity of CTLs

To validate enrichment pathways mentioned above, BCAT2 overexpression (BCAT2 OE) and BCAT2 knockdown (BCAT2 KD) human (T24)/murine (MB49) bladder cancer cell lines were constructed successfully (Figure S15A–D, Supporting Information) and were tested with high‐throughput RNA sequencing. Expectedly, GO analysis of T24 cell lines revealed that DEGs were significantly enriched in the pathways of chemokine‐mediated signaling, response to chemokine, leukocyte migration and leukocyte migration (Figure 3J). KEGG pathway analysis of T24 cell lines showed that chemokine signaling pathway, cytokine–cytokine receptor interaction pathway and bladder cancer pathway were significantly enriched (Figure 3K). Similarly, several critical cytokine/chemokine related pathways were found in GO and KEGG analyses of MB49 cell lines (Figure S16A,B, Supporting Information).

**Figure 4:**
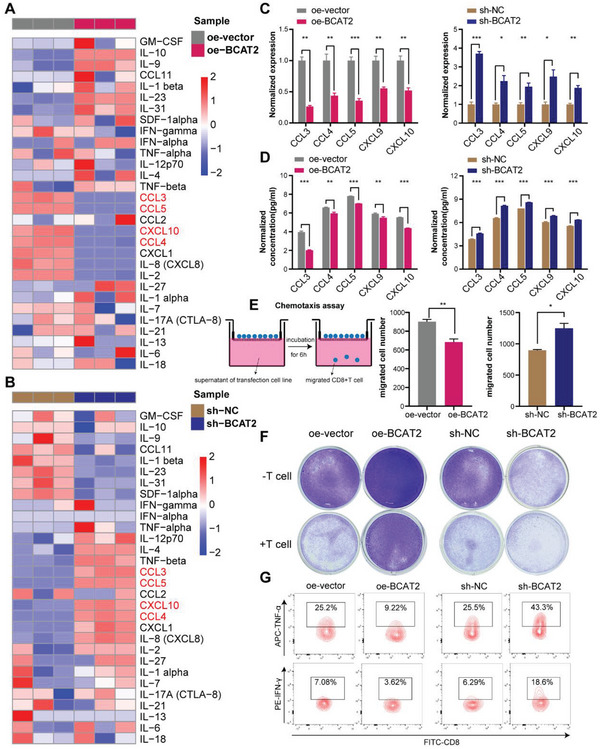
BCAT2 varies expression patterns of CD8^+^T‐cell‐related chemokines and inhibits cytotoxic capacity of CTLs. A,B) Heatmaps of ProcartaPlex multiple immunoassays display secretion variation of common cytokines and chemokines in BCAT2 OE, BCAT2 KD and negative control groups (*n* = 3 per group). C,D) Histograms show normalized mRNA expression levels and protein secretion concentrations of CCL3, CCL4, CCL5, CXCL9, and CXCL10 in BCAT2 OE (left), BCAT2 KD (right) and negative control groups (*n* = 3 per group). *p < 0.05; **p < 0.01; ***p < 0.001. E) Chemotaxis assay indicates different chemotaxis ability of CTLs in BCAT2 OE, BCAT2 KD, and negative control groups (*n* = 3 per group). *p < 0.05; **p < 0.01. F) T‐cell‐mediated cancer cell killing assay indicates different killing abilities of T cells cocultured with BCAT2 OE, BCAT2 KD, and negative control cell lines (*n* = 3 per group). G) Flow cytometry analysis indicates different activities of CD8^+^T cells in different coculture groups (*n* = 3 per group).

Self‐evidently, various of cytokines and chemokines act critical roles in regulating TME. Therefore, it is necessary to screen cytokines/chemokines which are specifically regulated by BCAT2. Hence, ProcartaPlex multiple immunoassays were applied to identify secretion variation of cytokines/chemokines in human and mouse bladder cancer cell lines. Surprisingly, secretion levels of CCL3, CCL4, CCL5, and CXCL10 upregulated in human and murine BCAT2‐KD cell lines and downregulated in human and murine BCAT2‐OE cell lines (**Figure** **4**A,B; Figure S16C,D, Supporting Information). In previous study, CCL3, CCL4, CCL5, CXCL9, and CXCL10 were regarded as crucial chemokines for recruiting CD8^+^T cells into TME.^[^ ^12^ ^]^ Consequently, for better comprehensive analyzing CD8^+^T‐cell‐related chemokines, all of them were included for further validation. Strikingly, RNA expression levels of CCL3, CCL4, CCL5, CXCL9, and CXCL10 were significantly downregulated in BCAT2 OE cells and significantly upregulated in BCAT2 KD cells (Figure 4C). Similarly, outcomes of ELISA showed that secreted protein levels of them also had negative correlations with expression of BCAT2 (Figure 4D). These findings indicated that overexpression of BCAT2 inhibits transcriptome and protein levels of CD8^+^T‐cell‐related chemokines, including CCL3, CCL4, CCL5, CXCL9, and CXCL10. More importantly, chemotaxis assay revealed that BCAT2 OE cell line significant inhibited chemotaxis ability of CD8^+^T cells (Figure 4E, left). Conversely, cell supernatants of BCAT2 KD cell line were capable of attracting more CD8^+^T cells than its negative control (Figure 4E, right). These findings indicated that BCAT2 can affect chemotaxis capacity of CD8^+^T cells by regulating mRNA and protein level of related chemokines. Furthermore, T‐cell‐mediated cancer cell killing assay was used to explore the cytotoxicity variation of T cells after direct contact with tumor cells expressed different level of BCAT2. As shown in Figure 4F and Figure S17A,B (Supporting Information), overexpression of BCAT2 on tumor cells directly suppressed cytotoxic function of T cells and knock down of BCAT2 on tumor cells significantly reversed the tendency. Flow cytometry analysis of collected T cells found that proportions of CD8^+^TNF‐*α* ^+^ T cells and CD8^+^IFN‐*γ* ^+^ T cells had significant differences in different coculture systems, which meant massive change on activity of CD8^+^T cell (Figure 4G; Figure S17C,D, Supporting Information). Collectively, BCAT2 is capable of inhibiting recruitment ability of CD8^+^T cells by regulating related chemokines levels as well as suppressing activities of CTLs by direct contact.

Additionally, the outcome of T‐cell‐mediated cancer cell killing assay (without T cells group) can infer that BCAT2 plays an oncogenic role in bladder cancer. Therefore, plate colony assay and transwell migration/invasion assay were utilized to validating this hypothesis. As anticipated, overexpression of BCAT2 significantly enhanced proliferation, migration and invasion ability of tumor cells and knockdown of BCAT2 markedly suppressed these behaviors (Figure S18A–F, Supporting Information).

### 2.4 Validation of Exclusive Spatial Relationship between BCAT2^+^ Tumor Cell and CD8^+^T Cell by TMA of Xiangya BLCA Cohort

**Figure 5:**
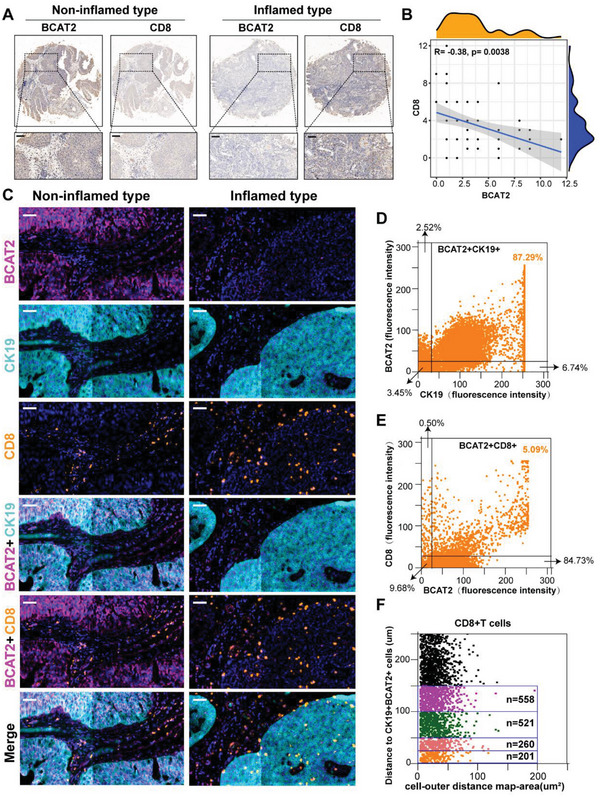
Validation of exclusive spatial relationships of BCAT2^+^ tumor cells and CD8^+^T cells by TMA of Xiangya BLCA cohort. A) IHC image of BCAT2 and CD8 in inflamed and noninflamed types of TME. Scale bar: 50 µm. B) Correlation between BCAT2 and CD8 on the basis of IHC scores of them in overall TMA. C) Multicolor IF image of BCAT2, CK19, CD8, and combination index in inflamed and noninflamed types of TME. BCAT2^+^ cell (pink), CK19^+^ cell (cyan), CD8+T cell (orange), and cell nucleus (blue). Scale bar: 50 µm. D,E) Detailed coexpression rates of BCAT2^+^CK19^+^ and BCAT2^+^CD8^+^ cells in the typical sample. F) Distance gradient analysis (0–25, 25–50, 50–100, and 100–150 µm) of CD8^+^T cells around BCAT2^+^CK19^+^ cells in the typical sample.

According to outcomes of bulk RNA‐seq, scRNA‐seq, and vitro experiment, we demonstrated that BCAT2 plays an immunosuppressive role in BLCA by suppressing recruitment and cytotoxicity of CD8^+^T cells. However, interaction of BCAT2^+^ tumor cell and CD8^+^T cell is still unknown in human tissue level. In Xiangya BLCA cohort, representative images and overall score of IHC revealed a negative correlation between BCAT2 and CD8 (*R* = −0.38, *p* = 0.0038) (**Figure** **5**A,B). Multicolor IF staining of BCAT2^+^ tumor cells (BCAT2^+^CK19^+^) and BCAT2^+^CD8^+^T cells were conducted in TMA and semiautomatic analyzed using TissueFAXS panoramic quantification platform. As shown in Figure 5C, the expression scope of BCAT2 and CK19 was largely overlapped. Conversely, the expression scope of BCAT2 and CD8 was distinct separate. Detailed coexpression proportions of BCAT2^+^CK19^+^ cells and BCAT2^+^CD8^+^T cells were 87.29% and 5.09% (Figure 5D,E), which was consistent with expression pattern of BCAT2 in scRNA‐seq. In overall TMA, there was still a significant difference on coexpression proportion between them (Figure S19A, Supporting Information). More importantly, in multidimensional distance gradient analysis (0–25, 25–50, 50–100, and 100–150 µm) around BCAT2^+^ tumor cells, counts of CD8^+^T cells were gradually increasing from near to far (Figure 5F; Figure S19B, Supporting Information). Together, we demonstrated that on the human tissue level, BCAT2 also mainly expressed on tumor cells and BCAT2^+^ tumor cell has exclusive spatial relationship with CD8^+^T cell.

### 2.5 Loss of BCAT2 Enhances Efficacy of Anti‐PD‐1 Therapy

**Figure 6:**
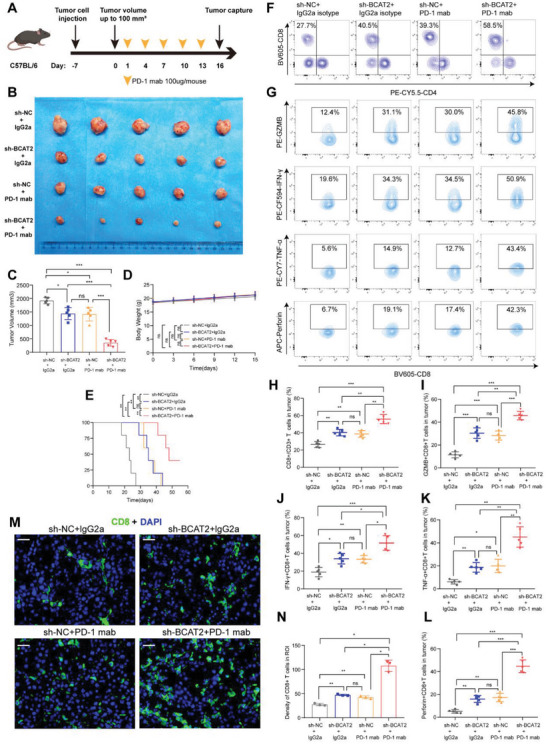
Loss of BCAT2 enhances efficacy of anti‐PD‐1 therapy. A) Flow diagram of treatment plan. B) Harvested tumors of different therapy regimens (*n* = 5 per group). C–E) Quantification of tumor volume, body weight and survival time in different therapy regimens (*n* = 5 per group). ns: no significance; *p< 0.05; **p < 0.01; ***p < 0.001. F) Contour plots indicate proportion of CD8^+^T cells; G) proportions of GZMB^+^CD8^+^T cells, IFN‐*γ* ^+^CD8^+^T cells, TNF‐*α* ^+^CD8^+^T cells, and Perforin^+^CD8^+^T cells in different therapy regimens. H) Quantified scatter plots exhibit proportion of CD8^+^T cells; I–L) proportions of GZMB^+^ CD8^+^T cells, IFN‐*γ* ^+^ CD8^+^T cells, TNF‐*α* ^+^ CD8^+^T cells, and Perforin^+^ CD8^+^T cells in different therapy regimens (*n* = 5 per group). ns: no significance; *p<0.05; **p < 0.01; ***p < 0.001. M,N) IF image and quantified histogram show densities of CD8^+^T cells in different therapy regimens (*n* = 3 per group). Scale bar: 20 µm. CD8^+^T cell (green) and cell nucleus (blue). ns: no significance, *p<0.05; **p < 0.01; ***p < 0.001.

With prominently adverse impact on TME and negative close correlation to inhibitory checkpoint blockade, it arouses us great interest to explore synergistic effect of BCAT2 loss and anti‐PD‐1 treatment. Ahead of immunotherapy, subcutaneous bladder cancer model was built by BCAT2 KD and control murine cell lines. Then, anti‐PD‐1 therapy and control therapy were applied to tumor‐bearing mice (**Figure** **6**A). Consistently with its oncogenic role and regulation mechanism on chemokines in vitro, BCAT2 deficiency inhibited tumor growth and prolonged survival time in vivo, by upregulating CD8^+^T related chemokines (Figure 6B–E; Figure S20A, Supporting Information). In the aspect of immunotherapy efficacy, although anti‐PD‐1 monotherapy could partly decrease tumor burden, cotreatment of BCAT2 KD and anti‐PD‐1 monoclonal antibody (mab) indicated a better tumor suppression effect and gained more survival benefit. On the scheduled day, tumors were harvested and prepared for further analysis. A part of them were digested into single‐cell suspension and conducted flow cytometry analysis. With similar population of leukocytes and T cells (Figure S20B–E, Supporting Information) in tumor, a higher proportion of CD8^+^T cells was shown in BCAT2 deficiency group than in negative control group. Similarly, combination therapy group had a more massive infiltration of CTLs than monotherapy group (Figure 6F–H). More importantly, cytotoxicity indicators (GZMB, IFN‐*γ*, TNF‐*α*, and Perforin) of CTLs were also reinforced in murine tumor with BCAT2 loss and combination treatment compared to corresponding control (Figure 6G,I–L). The other parts of tumors were made into frozen sections and implemented IF staining. As expected, density of CD8^+^T cells in region of interest (ROI) showed same tendency with flow cytometry analysis (Figure 6M,N). These findings indicated that BCAT2 deficiency on tumor cells can shape an inflamed TME and have a great efficacy with cotreatment of immunotherapy.

To figure out the role of CD8^+^T cells in the synergistic effect of cotreatment, CD8*α* mab was applied to antagonize them in immune‐competent mice (Figure S21A, Supporting Information) and validated its depletion effect by flow cytometry and IF (Figure S21B,C, Supporting Information). Obviously, after depletion, tumor burden and survival time were significantly reversed (Figure S21D–G, Supporting Information). These findings indicated that CD8^+^T cells act an indispensable part in synergistic effect of combination therapy.

Furthermore, alongside on tumor cells, a small proportion of BCAT2 also expressed on CD8^+^T cells. Hence, we further explored whether different expression level of BCAT2 on CD8^+^T cells can affect their activities. After staining single‐cell suspension of murine spleen with BCAT2 and T cell related fluorescent antibodies, we compared proportions of CD8^+^ TNF‐*α* ^+^T and CD8^+^ IFN‐*γ* ^+^T cells between BCAT2^+^CD8^+^ and BCAT2^−^CD8^+^ groups. Interestingly, we found that proportions of them were similar between two groups, revealing that activity of CD8^+^T cell is independent of its expression level of BCAT2 (Figure S22A–C, Supporting Information).

### 2.6 BCAT2 Predicts Response to Immunotherapy in Several Real‐World Immunotherapy Cohorts

Above findings have demonstrated an immunosuppressive role of BCAT2 in TME and synergetic effect of combining BCAT2 loss with anti‐PD‐1 therapy. However, its predictive potential on immunotherapy efficacy is unclear. Therefore, in TCGA‐BLCA cohort, enrichment scores of immunotherapy‐related pathways were compared between high and low BCAT2 groups. As shown in Figure S23A (Supporting Information), low‐BCAT2 group had more active genes in immunotherapy‐related pathways, which can infer that low expression of BCAT2 represents a more sensitive state to immunotherapy.

**Figure 7:**
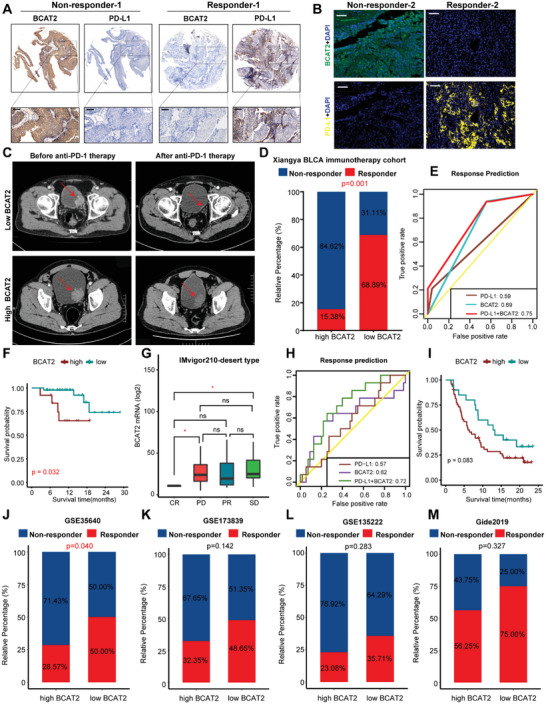
BCAT2 predicts response to immunotherapy in several real‐world immunotherapy cohorts. A) IHC image of BCAT2 and PD‐L1 between responder and nonresponder in Xiangya BLCA immunotherapy cohort. Scale bar: 50 µm. B) Multicolor IF image of BCAT2 and PD‐L1 between responder and nonresponder in Xiangya BLCA immunotherapy cohort. Scale bar: 50 µm. BCAT2^+^cell (green), PD‐L1^+^cell (yellow), and cell nucleus (blue). C) CT image of difference on immunotherapy efficacy between individual with high and low expression level of BCAT2. Red arrow points to the tumor area. D) Overall difference on response rate between high and low BCAT2 groups in Xiangya BLCA immunotherapy cohort. E) Prediction accuracy of BCAT2, PD‐L1, and combination index (BCAT2+PD‐L1) on response to immunotherapy in Xiangya BLCA immunotherapy cohort. F) Difference on disease‐free survival (DFS) between high and low BCAT2 groups in Xiangya BLCA immunotherapy cohort. G) Differences on expression level of BCAT2 among CR, PR, SD, and PD groups in desert type of IMvigor210 cohort. *p < 0.05; ns: no significance. H) Prediction accuracy of BCAT2, PD‐L1, and combination index (BCAT2+PD‐L1) on response to immunotherapy in desert type of IMvigor210 cohort. I) Difference on overall survival (OS) between high and low BCAT2 groups in desert type of IMvigor210 cohort. J–M) Differences on response to immunotherapy between high and low BCAT2 groups in GSE35640, GSE173839, GSE135222, and Gide2019 cohorts.

For further validation, 58 muscle‐invasive bladder cancer (MIBC) patients who accepted neoadjuvant anti‐PD‐1 therapy in our hospital were included in Xiangya BLCA immunotherapy cohort. Representative IHC, IF, and CT images of responder and nonresponder implied that expression level of BCAT2 has a close relation to efficacy of immunotherapy—individual with low BCAT2 expression level is more likely response to immunotherapy than individual with high BCAT2 expression level (**Figure** **7**A–C). Moreover, IHC score of Xiangya BLCA immunotherapy cohort and mRNA expression matrix of IMvigor210 cohort indicated a robust negative correlation between BCAT2 and PD‐L1 (*R* = −0.4, *p* = 0.002; *R* = −0.41, *p* < 0.001) (Figure S23B,C, Supporting Information). Hence, we direct compared efficacy of immunotherapy between high and low BCAT2 groups in Xiangya BLCA immunotherapy cohort and found that low BCAT2 group had a significant higher proportion of responder than high BCAT2 group (*p* = 0.001) (Figure 7D). Moreover, we assessed the prediction values of BCAT2, PD‐L1 and combination index (BCAT2+PD‐L1) on pathological response to immunotherapy and found the great performance of combination index (BCAT2+PD‐L1) on prediction accuracy (Figure 7E). Inspiringly, in the aspect of survival outcome, low BCAT2 group had a longer disease‐free survival (DFS) than high BCAT2 group (*p* = 0.032) (Figure 7F), demonstrating the prognosis value of BCAT2 in BLCA immunotherapy.

In clinical, individual with desert TME more likely has a limited immunotherapy efficacy than individual with inflamed TME, further highlight the importance of forecasting indicator. Consequently, we selected all the individuals with desert TME in IMvigor210 cohort and conducted comprehensive assessment. In direct comparison of expression level of BCAT2 among different response groups, CR group (responder) had a significant lower expression level of BCAT2 than SD and PD groups (nonresponder) (Figure 7G). More importantly, combination index (BCAT2+PD‐L1) also had a terrific performance on prediction accuracy (Figure 7H). Although there was no significant difference on overall survival (OS) between high and low BCAT2 groups in desert type of IMvigor210 cohort, prognosis tendency still similar with the outcome of Xiangya BLCA immunotherapy cohort (Figure 7I).

Apart from PD‐L1, microsatellite instability (MSI) is the other essential predictor on efficacy of immunotherapy. The status of mismatch repair (MMR)—proficient MMR (pMMR) and deficient MMR (dMMR) keep high conformity with low‐frequency MSI (MSI‐L) and high‐frequency MSI (MSI‐H).^[^ ^13^ ^]^ Hence, we conducted a comprehensive evaluation of all the individuals in Xiangya BLCA immunotherapy cohort based on staining results of four MMR marker genes (MLH1, MSH2, MSH6, and PMS2). The outcome revealed that a significant higher proportion of dMMR (MSI‐H) in low BCAT2 group comparing to in high BCAT2 group (*p* = 0.02) (Figure S23D,E, Supporting Information). Besides, we evaluated the predictive values of MMR, BCAT2 and combination index (MMR+BCAT2) on immunotherapy efficacy and found that combination index (MMR+BCAT2) had an eligible performance on prediction accuracy (Figure S23F, Supporting Information). Together, these findings demonstrated that BCAT2 is qualified to act as a complementary predictor on efficacy of BLCA immunotherapy.

At last, predictive value of BCAT2 on response to immunotherapy was explored in various cancer types. We found that individuals with high expression of BCAT2 showed a significant poorer response rate (*p* = 0.04) in GSE35640 cohort (melanoma) (Figure 7J). Although there were no significant differences on response rate between high and low BCAT2 group in GSE173839 (breast cancer), GSE135222 (NSCLC) and Gide 2019 cohort (melanoma), patients with low expression of BCAT2 were still more likely to have positive response to immunotherapy (Figure 7K–M).

### 2.7 The Value of BCAT2 in Forecasting Molecular Subtype and Guiding Precision Therapy

With great heterogeneity exists in traditional histology classification, increasing evidences showed that molecular subtype is able to provide more accurate classification on the basis of transcriptome profile in BLCA.^[^ ^14^ ^]^ Furthermore, with similar immunological characteristic in the same subtype, molecular classification has great potential to predict TME and guide precision therapy in clinical.^[^ ^15^ ^]^ Through comprehensive analysis on different molecular subtype systems in TCGA‐BLCA cohort, we found that individuals with low expression of BCAT2 are more likely classified into basal subtype, accompanied with activated pathways of basal differentiation, EMT differentiation, immune differentiation, interferon response and so on. Individuals with high expression of BCAT2 are mainly classified into luminal subtype, accompanied with active pathways of luminal differentiation, urothelial differentiation and Ta (Figure S24A, Supporting Information). According to consensus of molecular subtype, basal subtype has more infiltration level of effector lymphocytes and more active IFN‐*γ* signaling pathway than luminal subtype,^[^ ^16^ ^]^ which imply a better efficacy of immunotherapy. Then, AUC was utilized to evaluate accuracy of prediction. Amazingly, except Baylor subtype system (AUC = 0.76), most of AUC were beyond 0.90 in other systems (Figure S24B, Supporting Information), which mean a robust prediction accuracy of BCAT2 in molecular subtype. To further explore its role in predicting efficacy of other therapies, we compared activity of epidermal growth factor receptor (EGFR) target therapy and radiotherapy related pathways between high and low BCAT2 groups. With obvious up‐regulated activities, patients in low‐BCAT2 group more likely have a positive response to EGFR‐target therapy and radiotherapy (Figure S24C, Supporting Information). For enhancing prediction reliability on molecular subtype and treatment sensitivity, eight BLCA cohorts (Xiangya BLCA cohort, GSE31684, GSE69795, GSE48075, GSE128702, GSE83586, GSE52329, and E‐MTAB‐1803) and one BLCA immunotherapy cohort (IMvigor210) were applied for external validation. Along with excellent accuracy of prediction, similar findings were found in these cohorts (Figures S25–S27, Supporting Information).

Hyperprogressive disease (HPD) is an extreme insensitive state to immunotherapy. For exploring influence of BCAT2 on HPD, HPD associated biomarkers were collected from previous studies^[^ ^17^ ^]^ and CNV of biomarkers were compared between low and high BCAT2 groups. Except for EGFR, CNV amplification rates of other HPD‐positive associated biomarkers (red symbol) were higher in high‐BCAT2 group, especially MDM2 (*p* = 0.0116). CNV deletion rates of HPD‐negative associated biomarkers (green symbol) were lower in high‐BCAT2 group (Figure S24D, Supporting Information). The outcome indicated that patient with high expression of BCAT2 have a potential risk of HPD during immunotherapy. Neoadjuvant chemotherapy (NAC) is a crucial therapy in BLCA. Markers forecasting response to NAC in BLCA were collected from review of Buttigliero et al.^[^ ^18^ ^]^ and their mutation rates were compared between two groups. Based on higher overall mutation rates of biomarkers and more frequent mutation of RB1 in low‐BCAT2 group, we speculated that individual with low expression of BCAT2 has a better efficacy of NAC in clinical (Figure S24E, Supporting Information). Hence, Drugbank database was utilized to compare efficacies of several common chemotherapy regimens between two groups and found that individuals with low expression of BCAT2 are more likely response to chemotherapy. Additionally, low‐BCAT2 group also showed better efficacies in common regimens of immunotherapy and ERBB therapy. Unexpected, patients with high expression of BCAT2 probably gain better curative effect in antiangiogenic therapy (Figure S24F, Supporting Information). Collectively, individuals with low expression of BCAT2 belong to an inflamed molecular subtype, basal subtype, which means a more positive response to immunotherapy, chemotherapy, radiotherapy, EGFR‐target therapy, and ERBB therapy. Unfortunately, patients with high expression of BCAT2 have a poor response to these treatments. However, antiangiogenic therapy maybe a glimmer of light to them.

## 3 Discussion

As a key enzyme in the catabolism process of BCAAs, previous researches mainly focused on metabolic‐related role of BCAT2 in the diseases, such as obesity, diabetes and arrhythmia. Ma et al. demonstrated that knock out BCAT2 in adipose tissue can accelerate adipose browning and thermogenesis, which leads to reduced obesity rate in mice.^[^ ^19^ ^]^ Through detecting blood samples of more than 2000 individuals, Gerszten et al. depicted a diabetes‐related metabolite profile and found that BCAT2‐transformed BCAAs act a crucial part in the development of disease.^[^ ^20^ ^]^ Portero et al. revealed that nonsense mutations of BCAT2^p.Q300*/p.Q300*^ result in elevated level of BCAAs and induce arrhythmias in mice.^[^ ^21^ ^]^ Recently, a couple of studies found that BCAT2 has a close relationship with cancer. Lei et al. revealed that acetylation‐mediated degradation of BCAT2 can retard development of pancreatic ductal adenocarcinoma (PDAC) by downregulating metabolism level of BCAAs.^[^ ^22^ ^]^ Wang et al. considered that restrained transcription level of BCAT2, leading to a decreased intracellular glutamate level, which stimulates ferroptosis of hepatoma cells.^[^ ^23^ ^]^ However, all of existing researches emphasized the influence of BCAT2‐mediated BCAAs catabolism on biological process of cancer rather than exploring a brand‐new mechanism.

In this study, we revealed immunosuppressive role of BCAT2 in TME for the first time. Application of immunotherapy redefines cancer treatment, with terrific living quality and prolonged survival time. Nonetheless, on account of heterogeneity of tumor‐immune ecological system, just a portion of individuals gain satisfactory efficacy. TME is a complicated system, composing of tumor cells, immune cells, stromal cells and capillaries.^[^ ^24^ ^]^ According to infiltration level of CTLs, TME can be classified into inflamed and noninflamed type in general.^[^ ^25^ ^]^ Growing evidence indicated that inflamed TME plays a positive role in eliciting effective antitumor immune response during therapy.^[^ ^26^ ^]^ Hence, we comprehensive analyzed multiple TME‐related indicators and found that high expression of BCAT2 shapes a noninflamed TME, with impeded cancer immunity cycle, repressive expression pattern of chemokine profile, MHC molecules and insufficient infiltration level of TIICs. In addition, BCAT2 is negative related to TIS and effector genes of ICB, which implies that individuals with high expression of BCAT2 are more likely not response to immunotherapy. For validating our hypothesis, we compared response rate between high and low BCAT2 group in Xiangya BLCA immunotherapy cohort and other immunotherapy cohorts. Surprisingly, there were significant differences in multiple cohorts, which indicated that BCAT2 is also capable of playing a predictor of immunotherapy's efficacy, especially in BLCA.

Furthermore, scRNA‐seq was used for exploring regulating mechanism of BCAT2 in TME and indicated that activity of cytokine/chemokine related signaling pathways, T cell chemotaxis signaling pathway and T cell migration signaling pathway have significant negative correlations with BCAT2. As a critical regulation network, cytokines and chemokines are indispensable for trafficking various immune cells into TME. Chemokine system has four superfamily: C, CC, CXC, and CX_3_C, with considerable redundancy on pairing of ligands and receptors.^[^ ^27^ ^]^ Cytokine can be divided into Th1, Th2, and Th17 subfamily based on their biological function.^[^ ^28^ ^]^ Through secretion of different level of them, the wrestle between tumor cell and immune cell is enough to reprogram the characteristic of TME.^[^ ^29^ ^]^ Nonetheless, among a bulk of cytokine and chemokine, it is requisite to screen out the most valuable cluster. By means of a 34‐cytokine and chemokine immunoassay panel, we revealed that chemokines including CCL3, CCL4, CCL5, CXCL9, and CXCL10, are robust negative correlated with expression of BCAT2 in human and murine bladder cancer cell. It is well known that CXCL9 and CXCL10 are responsible for recruiting CD8^+^T cells into TME.^[^ ^30^ ^]^ For ascertaining function of CCL3, CCL4, and CCL5, we looked up previous researches. Harlin et al. made use of protein arrays and qPCR to reveal that upregulation of CCL3, CCL4, and CCL5 are capable of promote migration of CD8^+^T cells into melanoma.^[^ ^31^ ^]^ Noman et al. found that increased secretion population of CCL5 assists to establish a proinflammatory TME by trafficking CTLs into colorectal cancer tissue.^[^ ^32^ ^]^ Through IHC and RNA‐seq of chemokine profile in multiple cohorts, Romero et al. demonstrated that CCL4 and CCL5 have a strongly association with infiltration level of CD8^+^T cells in pancreatic cancer.^[^ ^33^ ^]^ Hence, we considered that CD8^+^T‐cell‐related chemokines are major chemokines regulated by BCAT2 in bladder cancer.

Although we demonstrated the robust negative correlations between BCAT2 and CD8^+^T cell related chemokines, the underlying regulatory mechanism between them still need to further explore. The study of Peterson er al. demonstrated that activation of MAP kinase (MAPK) signaling pathway can induce production of CX3CL1.^[^ ^34^ ^]^ Xu et al. revealed that JAK‐STAT signaling pathway regulated secretion of Th1 related chemokines, causing a decreased infiltration level of TILs.^[^ ^35^ ^]^ Recently, Peng et al. found that demethylase JMJD3 can inhibit the expression of CD4^+^T‐cell‐related chemokines and reduce infiltration level of cytotoxicity T cells in the TME, representing a crucial role of epigenetic modification in regulating chemokine expression.^[^ ^36^ ^]^ Mayo et al. also revealed that an epigenetic inhibitor, histone deacetylase 1, has great capacity to suppress the expression of CXCL8 via activation of nuclear factor‐*κ*B (NF‐*κ*B) signaling pathway.^[^ ^37^ ^]^ Moreover, as symbols of tumor cell metabolism, aerobic glycolysis and reactive oxygen species (ROS), indicating great performances on inducing expression of CXCL8 and CXCL14 by elevating activities of signaling pathways of NF‐*κ*B and transcription factor activator protein 1 (AP‐1).^[^ ^38^ ^]^ Interestingly, in our study, signaling pathways of NF‐*κ*B, STAT, and MAPK were significantly enriched between high and low BCAT2 cell lines (Figure 3K; Figure S16A,B, Supporting Information). Hence, combining with above studies, we will further explore the key molecule in the regulation mechanism of BCAT2 and CD8^+^T related chemokines.

Limited objective response rate of monotherapy with ICB prompts advent of combination therapy strategy. For converting nonimmunologic TME into immunologic TME, Wolchok et al. applied cotreatment of nivolumab (anti‐PD‐1 therapy) and ipilimumab (anti‐CTLA‐4 therapy) in patients with melanoma and found an encouraging curative effect, ascribing to synchronous enhanced priming, activation and killing of CTLs.^[^ ^39^ ^]^ More than combining with other type of ICB, chemotherapy plus immunotherapy is s alternative therapy option. As a first‐line treatment regimen for advanced urothelial carcinoma, a couple of studies found that cisplatin‐based chemotherapy is capable of shaping a proinflammatory TME by increasing expression level of MHC I class on dendritic cells and damaging MDSCs and Tregs.^[^ ^40^ ^]^ Coincidentally, Homma et al. revealed that another common chemotherapy drug for bladder cancer—gemcitabine—can improve infiltration quantity of CD8^+^T cells and impair immunosuppressive immune cells in TME.^[^ ^41^ ^]^ In our preclinical animal experiment, cotreatment of BCAT2 loss and anti‐PD‐1 mab displayed a more distinct antitumor efficacy compared to monotherapy of anti‐PD‐1 mab in immune‐competent mice, which provides an innovative treatment strategy for ICB‐resistance patients. Meanwhile, through flow cytometry analysis and IF, we demonstrated that knocking down of BCAT2 not only recruits more population of CTLs into TME but also enhances killing activity of CTLs. Similarly, Peng et al. found that overexpression of LGALS2 decreases the quantity of infiltrating CTLs as well as cytotoxic biomarkers in breast‐cancer‐bearing mice.^[^ ^42^ ^]^ Yang et al. illustrated that CXCL13 is capable of heightening response to immunotherapy in ovarian cancer mouse model by increasing infiltration level of CD8+T cells and secretion level of GZMB, IFN‐*γ* and IL‐2.^[^ ^43^ ^]^ Accompanying stronger anticancer response, combination therapy further upsets existed immune feedback loop, which probably leads to severe side effects. According to expression pattern of BCAT2 in scRNA level, specifically expressed in tumor cells rather than in immune cells and endothelial cells, we can preliminarily infer that BCAT2 loss or inhibitor of BCAT2 less likely results in terrible adverse effects. However, optimal dosage and interval time of BCAT2 inhibitor and anti‐PD‐1 mab in combination therapy still need to explore.

For guiding precision therapy in individual, we correlated BCAT2 to molecular subtype of BLCA and critical biomarkers of various therapies. Based on credible prediction in multiple cohorts, we found that immunotherapy, chemotherapy, radiotherapy and EFGR‐target therapy are suitable for patient with low expression of BCAT2. Antiangiogenic therapy is recommended to patient with high expression of BCAT2. Different from complicated detection process of molecular subtype, BCAT2 is a portable predictor for precision therapy.

Inevitably, there are some limitations in our studies. First, the sample sizes and the follow‐up time of Xiangya BLCA cohort and Xiangya BLCA immunotherapy cohort were limited. We need to further enlarge the sample size and continue standardized follow‐up. Second, as a real‐world cohort, Xiangya BLCA immunotherapy cohort contained different surgery options which probably cause a potential bias. We will further enlarge our cohort and conduct subgroup analysis based on same surgery option. Third, cooperative effect of combination therapy in vivo need to further validate using system application of BCAT2 inhibitor.

In summary, as an immunosuppressive role in TME of BLCA, BCAT2 is an emerging target in combination therapy of ICB and an accurate biomarker of precision therapy.

## 4 Experimental Section

### Data Source and Processing–Multiple Xiangya Cohorts

Xiangya BLCA cohort: As illustrated in previous study,^[^ ^44^ ^]^ 56 eligible BLCA patients accepted surgical treatment (TURBT (transurethral resection of bladder tumor) or radical cystectomy) in Xiangya Hospital. All the samples were conducted high throughput RNA sequencing (RNA‐seq) to acquire transcriptome information. Then, data of RNA‐seq, clinicopathologic features and follow‐up information of these patients were utilized to construct Xiangya BLCA cohort (Table S1, Supporting Information).

Xiangya BLCA immunotherapy cohort: 58 MIBC patients were included in Xiangya BLCA immunotherapy cohort. All the samples were obtained by diagnostic TURBT before implement of neoadjuvant anti‐PD‐1 therapy. After at least two cycles standard neoadjuvant immunotherapy (NAI), TURBT or radical cystectomy (RC) were conducted based on treatment response and patients’ willing. 17 individuals with complete response (CR) and 16 individuals with partial response (PR) were classified into responders, 17 individuals with stable disease (SD), and 8 individuals with progressive disease (PD) were classified into nonresponders. The detailed clinicopathological characteristics were listed in Table S2 (Supporting Information).

Xiangya scRNA cohort: Three samples of muscle‐invasive bladder cancer were implemented scRNA‐seq, constituting Xiangya scRNA cohort. Detailed preparation of single‐cell suspension, data transformation and cell quality control have been elaborated in previous articles.^[^ ^45^ ^]^ In simple terms, three tumor samples were loaded on a Chromium Single Cell Controller instrument (10×Genomics, USA) to produce single‐cell gel beads in suspension. Seurat R package was applied to transformed count matrix into Seurat format. Four standards were set up for excluding low quality cells in the matrix: unique molecular identifier (UMI) amount < 1000, gene quantity < 200, log_10_GenesPerUMI < 0.70, and mitochondrial‐originated UMI counts > 20%. After integrating the samples based on the top 3000 variable traits, principal component analysis (PCA) and Findclusters algorithm were employed to identify main cell clusters. Based on level of copy number variation (CNV) in epithelial cells, malignant bladder carcinoma cells were selected from clusters.

The study plan was approved by the ethics committee of Xiangya Hospital, Central South University (Item number: 2021101175). All the specimens were collected complying with informed consent right.

### The Cancer Genome Atlas (TCGA) Database

RNA expression matrix, survival outcome and CNV of 33 types of carcinomas were acquired from UCSC Xena database. Log2 transformation was employed to normalize RNA‐seq data and GISTIC algorithm was applied to process CNV data.

### Gene Expression Omnibus (GEO) Database

Transcriptome data of 1740 samples from nine BLCA cohorts: GSE31684 (93 samples), GSE48075 (142 samples), GSE69795(61 samples), GSE32894 (308 samples), GSE48276 (116 samples), GSE83586 (307 samples), GSE86411 (132 samples), GSE52329 (20 samples), GSE87304 (305 samples), and GSE128702 (256 samples) were obtained from GEO database.

RNA‐seq data of three immunotherapy cohorts: GSE35640 (14 samples, melanoma, and MAGE‐A3 therapy), GSE173839 (105 samples, breast cancer, and anti‐PD‐L1 therapy) and GSE135222 (27 samples, non‐small cell lung carcinoma (NSCLC), and anti‐PD‐1/PD‐L1 therapy) were downloaded from GEO database.

Single‐cell transcriptomic characterization of two BLCA scRNA cohorts: GSE135337 (8 samples) and GSE145137 (3 samples) were obtained from GEO database. Data processing of them were similar with Xiangya scRNA cohort.

### Other Databases

RNA expression matrix and clinicopathologic characteristics of immunotherapy cohorts: IMvigor210 (348 samples, bladder cancer, and anti‐PD‐1 therapy) and Gide2019 (58 samples, melanoma, anti‐PD‐1, or anti‐CTLA‐4 therapy) were collected from http://research‐pub.gene.com/IMvigor210CoreBiologies and TIDE database (http://tide.dfci.harvard.edu/). Data of RNA‐seq and clinical characteristics of a BLCA cohort—E‐MTAB‐1803 (85 samples)—was downloaded from ArrayExpress (https://www.ebi.ac.uk/arrayexpress/). BCAT2 expression level in various types of cancer cells lines were downloaded from Cancer Cell Line Encyclopedia (CCLE) database.

Specific clinicopathologic and follow‐up information of public databases were listed in our previous studies.^[^ ^44^, ^46^ ^]^

### Assessment of Immunological Identity of TME in BLCA

As depicted in our previous study,^[^ ^44^ ^]^ a comprehensive evaluation of immunological trait of TME in BLCA were summarized. Tracking tumor immunophenotype (TIP) website (http://biocc.hrbmu.edu.cn/TIP/) was used to assess activities of cancer immunity cycles, which reflected the effectiveness of antitumor immunity. It is separated into seven concrete steps: release and presentation of tumor antigen (steps 1 and 2), startup and activation of effector T cells (step 3), trafficking and assisting diverse immune cells into TME (steps 4 and 5), recognition and killing cancer cells (steps 6 and 7).^[^ ^47^ ^]^ Six independent algorithms (TIMER, CIBERSORT, CIBERSORT‐ABS, MCP‐COUNTER, TISIDB, and XCELL) were employed to calculate the infiltration levels of tumor‐infiltrating immune cells (TIICs).^[^ ^48^ ^]^ Furthermore, effector genes of immune cells, ligands and receptors of chemokines, major histocompatibility complex (MHC) molecules and immunostimulators were collected for evaluating immunological characteristic of TME multidimensionally. T cell‐inflamed score (TIS) and markers of ICB were utilized to assess host sensitivity state to immunotherapy.^[^ ^49^ ^]^

### Enrichment Pathways Analysis

Limma R package with empirical Bayesian function was employed to screen differentially expressed genes (DEGs) between high and low expression of BCAT2 in RNA expression matrix.^[^ ^50^ ^]^ Screening thresholds were |log (fold change) (log FC) |> 1.5 and the adjusted *p*‐value < 0.05. Gene Ontology (GO) and Kyoto Encyclopedia of Genes and Genomes (KEGG) analyses were conducted on identified DEGs by ClusterProfiler R package.^[^ ^51^ ^]^

Seurat R package with Findmarker algorithm was applied to calculate expression level of BCAT2 on epithelial cells in scRNA‐seq. On the basis of varied gene expression ranking by fold change (FC) value, gene set enrichment analysis (GSEA) was implemented for judging positive and negative correlation.

### Identification of Immune‐Related DEGs

ESTIMATE R package was employed to compute immune and stromal score in TCGA‐BLCA cohort. According to median value of two scores and expression level of BCAT2, Limma R package was applied to identify positive/negative immune‐related and stromal‐related DEGs. VennDiagram R package was used to take intersection of various groups and make certain the common DEGs.

### Classification of Individuals Using Molecular Subtypes of BLCA

Seven molecular subtypes of BLCA (UNC, Baylor, TCGA, MDA, Lund, CIT, and Consensus) were widely applied in clinical for assessing characteristic of TME and efficacies of various therapies.^[^ ^52^ ^]^ In consideration of complex interaction of various subtypes, we collectively divided them into two main categories—basal and luminal subtypes.^[^ ^16^ ^]^ R packages of ConsensusMIBC and BLCAsubtyping were employed to classify individual into specific subtype. After normalization, area under the ROC curve (AUC) was utilized to assess the reliability of BCAT2 in predicting classification.

### Precision Therapy Assessment of Patients

As illustrated in our previous study,^[^ ^53^ ^]^ a series of critical gene signatures, which can predict efficacy of chemotherapy, radiotherapy, targeted therapy, and immunotherapy, were gathered from different studies and databases.^[^ ^16^, ^54^ ^]^ ssGSEA algorithm was used to reckon the enrichment scores of gene signatures.^[^ ^55^ ^]^

### Cells Lines

Human bladder cancer cells (T24) and murine bladder cancer cell (MB49) were purchased from Procell Life Science & Technology (Wuhan, China) and Meisen CTCC (Jinhua, China), respectively. They were maintained in DMEM medium (BasalMedia, China) with 10% fetal bovine serum (FBS) (BI, Israel), 1% penicillin and streptomycin (NCM Biotech, China), and cultured in incubator at 37 °C temperature containing 5% CO_2_.

### Construction and RNA‐seq of Stable Transfection Cells

Lentiviral vector plenti‐BCAT2‐flag‐puromycin (GV341) was designed and constructed by GeneChem (Shanghai, China) for stable BCAT2‐overexpression (BCAT2 OE) cell line and its negative control (oe‐vector). In addition, BCAT2‐short hairpin RNA (shRNA) was cloned into GV112‐lentiviral vector by GeneChem (Shanghai, China) for stable BCAT2‐knock down (BCAT2 KD) cell line. Target sequences of shRNA were listed in Table S3 (Supporting Information).

Then, recombinant BCAT2 lentiviruses were transfected into objective cells according to manufacturer's instructions. 2 *µ*g mL^−1^ puromycin (Amersco, USA) was employed to filter stable transfection cells for three days. Western blot and quantitative reverse‐transcription PCR (qRT‐PCR) were applied to validate efficacy of transfection. Eventually, stable transfection cell with best efficacy was picked for RNA‐seq and further experiments.

Three duplicate samples of each cell line were sent to BGI RNA‐seq platform (Shenzhen, China). Criteria of selecting DEGs and analysis of enrichment pathways were same as depicted in 2.3 (enrichment pathways analyses).

### ProcartaPlex Multiple Immunoassays for Detecting Secretion Level of Chemokines and Cytokines of Cancer Cell Lines

Human ProcartaPlex immunoassay panel (Cat: EPX340‐12167‐901) and mouse ProcartaPlex immunoassay panel (Cat: EPX360‐26092‐901) were purchased from ThermoFisher Scientific (Massachusetts, USA) for detecting protein expression levels of more than 30 crucial chemokines and cytokines in stable transfection cancer cells. In brief, cell culture supernatants were collected from 24‐well plate and performed centrifugation for removing cells and cell debris. Then, clarifying supernatants was incubated with beads in panel following manufacturer's instruction. Luminex detection platform (ThermoFisher Scientific, USA) was used to perform quantitative analysis on each sample. Undetected indicators were excluded from analysis.

### qRT‐PCR

Total RNA was isolated from cells by Cell Total RNA Isolation Kit and Animal Total RNA Isolation Kit (Foregene, China) according to manufacturer's protocol. cDNA was synthesized using UeIris II RT‐PCR System for First‐Strand cDNA Synthesis (US Everbright, China). qRT‐PCR was performed using SYBR Green qPCR Master Mix (US Everbright, China) on CFX Connect System (Bio‐Rad, USA). GAPDH was used as internal standard control. The primers were designed and synthesized by Sangon Biotech (Shanghai, China) and detailed primer sequences were listed in Table S4 (Supporting Information).

### ELISA

Concentrations of CCL3, CCL4, CCL5, CXCL9 (MIG), and CXCL10 (IP10) in human bladder cancer cell culture supernatants were determined by human ELISA kits following manufacturer's protocol (Proteintech, USA). Biotech microplate reader (ThermoFisher Scientific, USA) was employed to measure optical density (OD) values. In view of a huge diversity of secretion level among different cytokines and chemokines, we conducted data standardization (log 2 transformation) before analysis.

### Western Blot

RIPA buffer (NCM biotech, China) added with 1% Protease Inhibitor Cocktail (NCM biotech, China) was used to lyse cells. BCA Protein Assay Kit (NCM biotech, China) was employed to determine protein concentrations. After transferred onto PVDF membranes, cell proteins were incubated with primary antibodies and specific HRP‐conjugated secondary antibodies successively. Signals were captured by XRS imaging system (Bio‐Rad, USA). Primary antibodies include: anti‐BCAT2 antibody (Cat: ab95976, Abcam, USA) and anti‐GAPDH antibody (Cat: ab8245, Abcam, USA). Secondary antibodies include: HRP goat anti‐rabbit IgG (Cat: 7074, Cell Signaling Technology, USA) and HRP goat anti‐mouse IgG (Cat: 7076, Cell Signaling Technology, USA).

### T Lymphocyte‐Mediated Cancer Cell‐Killing Assay and Coculture Assay

Blood samples were collected from 10 healthy donators in our hospital. According to manufacturer's instruction, gradient centrifugation was employed to extract peripheral blood mononuclear cells (PBMCs) by Lymphoprep (Cat: 07851, StemCell Technologies, USA). In addition, Red Blood Cell Lysis Buffer (Solarbio, China) was used to eliminate red cells mixed with PBMCs. In order to activate T cells, PBMCs were cultured in DMEM medium (Gibco, USA), supplementing Recombinant Human IL‐2 (10 ng mL^−1^, Cat: 202‐1L‐050, R&D, USA), and ImmunoCult Human CD3/CD28/CD2 T cell activator (25 *µ*L mL^−1^, Cat: 10970; STEMCELL Technologies, USA) for 1 week. At the ratio of 1:5, human bladder cancer cells (BCAT2‐OE, BCAT2‐KD and their negative controls) were cocultured with activated T cells in DMEM medium with anti‐CD3 antibody (100 ng mL^−1^, Thermo Scientific, USA) and IL‐2 (10 ng mL^−1^) from one donator in 12‐well plate for 72 h. Then, T cells were collected for flow cytometry analysis. The detailed gate strategy of flow analysis was shown in Figure S28 (Supporting Information). Remained cancer cells were stained by crystal violet and measured OD value at 570 nm by microplate reader.

Antibodies for flow cytometry analysis include: Zombie Aqua Fixable Viability Kit (Cat: 423101, Biolegend, USA), APC/ Cy7 anti‐human CD45 (Cat: 368516, Biolegend, USA), Pacific Blue anti‐human CD3 Antibody (Cat: 300329, Biolegend, USA), PerCP/Cy5.5 anti‐human CD4 Antibody (Cat: 317427, Biolegend, USA), FITC anti‐human CD8a Antibody (Cat: 301006, Biolegend, USA), PE/Dazzle 594 anti‐human TNF‐*α* Antibody (Cat: 502946, Biolegend, USA), and Brilliant Violet 711 anti‐human IFN‐*γ* Antibody (Cat: 502540, Biolegend, USA).

### Chemotaxis Assay

Chemotaxis assay was implemented in a 24‐well transwell plate with 3 µm core diameter (Corning, USA). Human CD8^+^T Cell Isolation Kit (Cat: 480012, Biolegend, USA) was used to extracted CD8^+^T cells from PBMC. 1 × 10^5^ isolated cells in 200 *µ*L volume were added into upper chamber and 600 *µ*L supernatants of different stable transfection cell lines were added into lower chamber. After incubation for 6 h at 37 °C, cells migrated into lower chamber were harvested and counted by flow cytometry.

### Cell Proliferation, Migration, and Invasion Assays

Plate colony formation assay was used to assess ability of cell proliferation. BCAT2‐OE, BCAT2‐KD, and negative control human bladder cancer cells were cultured into 6‐well plates per well. After incubating in 37 °C humidified atmosphere for 2 weeks, colonies were fixed and dyed by paraformaldehyde and crystal violet, respectively.

Transwell chambers with 8.0 µm pore polycarbonate membrane inserts (Corning, USA) were employed to evaluate capacity of cell migration and invasion in vitro. 1 × 10^5^ stable transfected cells were suspended in serum‐free medium and seeded into top chamber with or without Matrigel (Corning, USA), for invasion and migration assay separately. After incubating for 24 h (migration assay) and 48 h (invasion assay), cells adhering to lower surface of membrane were fixed and stained. Cells remained in top chamber were removed by swabs. Five random fields were selected under microscope to calculate cell numbers.

### Immunofluorescence (IF) and Immunohistochemistry (IHC)

Multicolor IF on TMAs of Xiangya BLCA Cohort and Xiangya BLCA immunotherapy Cohort was implemented by multiple fluorescent immunohistochemical staining kit (Absin, China). In brief, after dewaxing, rehydrating and antigen renovating, TMA was incubated with primary antibodies and secondary antibodies which prompts binding of different luciferins by tyramide signal amplification (TSA). Following wiping off unbound antibodies with citrate buffer, process of incubation was repeated twice. DAPI was employed to counterstained cell nucleus and images were scanned by Pannoramic MIDI platform (3DHISTECH, Hungary). Primary antibodies on TMA of Xiangya BLCA cohort include: anti‐BCAT2 (Cat: ab95976, Abcam, USA), anti‐Cytokeratin 19 (CK19) (Cat: ab52625, Abcam, USA), anti‐CD8 (Cat: ab237709, Abcam, USA) and DAPI (Invitrogen, USA). Secondary antibody and fluorochrome on TMA of Xiangya BLCA cohort include: HRP Goat Anti‐Rabbit/Mouse IgG, Absin 520 TSA Plus, Absin 570 TSA Plus, Absin 650 TSA Plus (abs50012, Absin). Primary antibodies on TMA of Xiangya BLCA immunotherapy cohort include: anti‐BCAT2 (Cat: ab95976, Abcam, USA), anti‐PD‐L1 (Cat: ab213524, Abcam, USA) and DAPI (Invitrogen, USA). Secondary antibody and fluorochrome on TMA of Xiangya BLCA immunotherapy cohort include: HRP Goat Anti‐Rabbit/Mouse IgG, Absin 520 TSA Plus and Absin 650 TSA Plus (abs50012, Absin). IF on murine tumor tissues was incubated with primary antibody—anti‐CD8 (Cat: 372902, BioLegend, USA)—and secondary antibody—anti‐mouse Alexa Fluor 488 dye conjugate (Invitrogen, USA) sequentially. DAPI was applied to visualized cell nucleus. Five random fields were selected under microscope to calculate positive staining cell numbers.

IHC on TMAs of Xiangya BLCA Cohort and Xiangya BLCA immunotherapy Cohort was conducted by SP‐9000 Kit (ZSGB‐BIO, China). After antigen retrieval and blocking, primary antibodies and secondary antibody were incubated with tumor tissues in turn. Then, diaminobenzidine (DAB) was applied to dye target antigens. Brown signal was regarded as positive staining. Score of staining intensity (absent = 0, weak = 1, moderate = 2, and strong = 3) multiplying score of positive percentage (no staining = 0, staining cells less than 25% = 1, 25%–50% staining cells = 2, 50%–75% staining cells = 3, and staining cells more than 75% = 4) was ultimate IHC score of samples. The scores of BCAT2/CD8/PD‐L1 less than six points were classified as low expression, the scores of them great than or equal to six points was classified as high expression. For judging individual's status on MMR, all the samples from Xiangya BLCA immunotherapy cohort were assessed based on staining outcomes of four MMR marker genes (MLH1, MSH2, MSH6, and PMS2). As depicted in previous studies,^[^ ^56^ ^]^ when all four marker genes were positive staining, the individual was marked as pMMR. When any of four marker genes was negative staining, the individual was marked as dMMR. Two independent pathologists were invited to conduct assessment. Antibodies include: anti‐BCAT2 (Cat: ab95976, Abcam, USA), anti‐CD8 (Cat: ab4055, Abcam, USA), anti‐PD‐L1 (Cat: ab213524, Abcam, USA), anti‐MLH1(Cat: A4858, Abcolonal, China), anti‐MSH2 (Cat: A22177, Abcolonal, China), anti‐MSH6 (Cat: A16381, Abcolonal, China), anti‐PMS2 (Cat: A4577, Abcolonal, China), and HRP Goat Anti‐Rabbit/Mouse IgG (ZSGB‐BIO, China).

### TissueFAXS Panoramic Analyses of Spatial Interaction in TME

For evaluating spatial interaction of BCAT2^+^ malignant cells and effector T cells, TissueFAXS panoramic platform (Tissue Gnostics, Austria) was utilized to scan and semiautomatically analyze objective cells stained by multicolor IF on TMA of Xiangya BLCA Cohort. The TMA was constituted of 1.5 mm core biopsies from paraffin‐embedded specimens of tumor tissues. In detailed, BCAT2^+^ cells, malignant cells and CD8^+^T cells were stained by specific primary antibodies and secondary antibodies. DAPI (Invitrogen, USA) was used to stain cell nucleus. Detailed steps have been described in the study of Makarevic et al.^[^ ^57^ ^]^ In brief, according to fluorescence intensities of different indicators and physical properties of cells, positive cells were marked and quantified. More important, spatial distributions of CD8^+^T cells around BCAT2^+^CK19^+^ cells were depicted by scatter plots on the basis of space distance (0–25, 25–50, 50–100, and 100–150 µm). Two experienced pathologists were invited to check the decision outcome of TissueFAXS panoramic platform.

### Animal Experiments

Female C57BL/6 mice (6–7 weeks) were obtained from Department of Laboratory Animal, Central South University. All operations on mice were checked and approved by Animal care and Use Committee of Xiangya Hospital, Central South University (Item number: 2021101175). Above all, to investigate the role of BCAT2 on tumor growth in vivo, BCAT2 KD MB49 cells (5 × 10^5^) and its control cells in 100 *µ*L medium volume were subcutaneous injected into right flank of mice. Moreover, after constructing subcutaneous tumor model successfully (tumor volume up to 100 mm^3^), 100 *µ*g InVivomAb anti‐mouse PD‐1 (Cat: BE0146, Bioxcell, USA) and IgG2a isotype control (Cat: BE0089, Bioxcell, USA) were intraperitoneal injected into per mouse, for assessing synergistic effect of BCAT2 loss and anti‐PD‐1 therapy. Anti‐PD‐1 therapy was implemented on mouse every 3 days and lasted up five courses. Further, for judging whether the cooperative effect of cotreatment was dependent on CD8^+^T cells. Accompanying combination therapy,100 *µ*g InVivoPlus anti‐mouse CD8*α* (Cat: BP0117, Bioxcell, USA) and IgG2b isotype control (Cat: BP0090, Bioxcell, USA) were employed to deplete CD8^+^T cells in mice. Body weights of mice were recorded every three days. On scheduled date, tumors were harvested, measured and prepared for flow cytometry analysis, IF staining and qRT‐PCR.

For exploring the interaction between expression level of BCAT2 on CD8^+^T cell and activity of CD8^+^T cell, spleens of female C57BL/6 mice (6–7 weeks) without tumor‐bearing were dissociated and prepared for single‐cell suspension for further analysis.

### Flow Cytometry Analysis

Murine tumors were ground and digested into single‐cell suspension by Collagenase IV (Cat: C5138, Sigma, USA), hyaluronidase (Cat: H3506, Sigma, USA) and DNase I (Cat: DN25, Sigma, USA). 70 µm cell strainers (BIOFIL, China) were used to filter out impurities and cell counter (Countstar, China) was employed to gain (3–5) × 10^6^ cells in each sample. After identifying live cells using Zombie Aqua Fixable Viability Kit (Cat: 423101, Biolegend, USA) and blocking Fc receptor with anti‐mouse CD16/32 antibody (Cat: 156603, Biolegend, USA), Cell membrane antigens were stained by APC‐Cy7 anti‐mouse CD45 (Cat: 103116, Biolegend, USA), BV421 anti‐mouse CD3 (Cat: 100341, Biolegend, USA), BV605 anti‐mouse CD8a (Cat: 100744, Biolegend, USA) and PerCP‐Cy5.5 anti‐mouse CD4 (Cat: 100540, Biolegend, USA). Then, eBioscienc Foxp3/Transcription Factor Staining Buffer Set (Cat: 2400632, Invitrogen, USA) was used for fixing and permeabilizing cell and nucleus membrane. Cytotoxic effect related antigens were dyed by PE anti‐human/mouse Granzyme B (Cat: 372208, Biolegend, USA), PE‐Cy7 anti‐mouse TNF‐*α* (Cat: 506324, Biolegend, USA), PE‐Dazzle 594 anti‐mouse IFN‐*γ* (Cat: 505846, Biolegend, USA), APC anti‐mouse Perforin (Cat: 154304, Biolegend, USA). The gating strategy of flow cytometry analysis was shown in Figure S29 (Supporting Information).

Anti‐human/mouse BCAT2 (Cat: A7426, Abclonal, China) was incubated with Flexable 488 antibody labeling kit (Cat: KFA001, Proteintech, USA) for synthesize personalized BCAT2 fluorescent antibody for flow cytometry analysis. Then, BCAT2 antibody and T‐cell‐related antibodies were mixed and added into single‐cell suspension of murine spleen for exploring the interaction between expression level of BCAT2 on CD8+T cell and activity of CD8+T cell. The gating strategy of flow cytometry analysis was shown in Figure S30 (Supporting Information).

Stained samples were detected on Cytek DxpAthena Flow cytometry (Cytek Biosciences, USA) and data were analyzed by Flowjo version10 software (BD Biosciences, USA).

### Statistical Analysis

Data were presented as mean ± SD. *t*‐test with or without Welch correction was utilized to compare continuous variables between two groups. One‐way ANOVA analysis with or without Brown–Forsythe and Welch tests was used to compare continuous variables between multiple groups. Chi‐squared test or Fisher's exact test was applied to compare dichotomous variables. Pearson or Spearman correlation coefficients test was employed to estimate intensity of correlation between different variables. Kaplan–Meier survival curve was employed to show prognostic analyses of dichotomous variables, log‐rank test was used to judge statistical difference. Two side *p* < 0.05 was defined as threshold of significance. R software (version 4.0) and GraphPad Prism8 were applied to process data in the study.

## Conflict of Interest

The authors declare no conflict of interest.

## Author Contributions

Conception and design: X.Z., J.H., and Z.C. Collection data from public databases: Z.C., Z.Y., J.C., Z.O., and M.C. Collection surgical specimens from Xiangya Hospital: D.D., J.L., C.C., C.Z. Bioinformatic analyses: J.H., H.L., and Z.L. Experimental works: Z.C., Z.Y., and J.H. Manuscript writing: Z.C. and J.H. Final approval of manuscript: all authors.

## Supporting information

Supporting Information

## Data Availability

Research data are not shared.
