# Supplementary material for: BCAT2 Shapes a Noninflamed Tumor Microenvironment and Induces Resistance to Anti‐PD‐1/PD‐L1 Immunotherapy by Negatively Regulating Proinflammatory Chemokines and Anticancer Immunity
Source: Adv Sci (Weinh). 2023 Jan 15;10(8):2207155. doi: 10.1002/advs.202207155 (PMC10015882; doi:10.1002/advs.202207155)
Supplement: Supplementary file 1 — Supporting Information [file ADVS-10-2207155-s001.pdf]

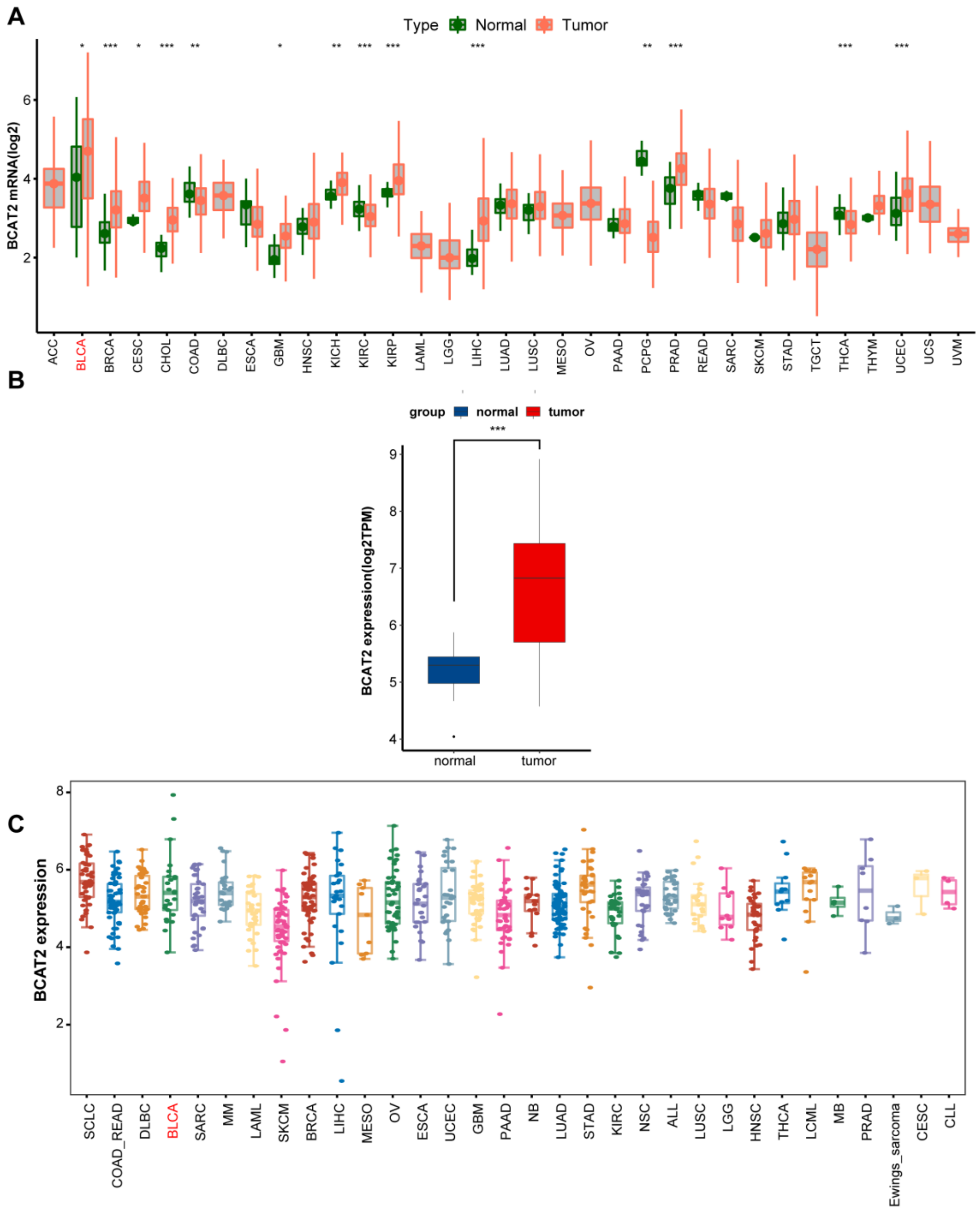

Supplementary Fig 1. Pan-cancer analysis of BCAT2 in tissues and cancer cell lines. (A) Expression patterns of BCAT2 in 33 types of cancer and adjacent normal tissues. (b) Expression pattern of BCAT2 in Xiangya BLCA cohort. (C) Expression patterns of BCAT2 in various cancer cell lines.

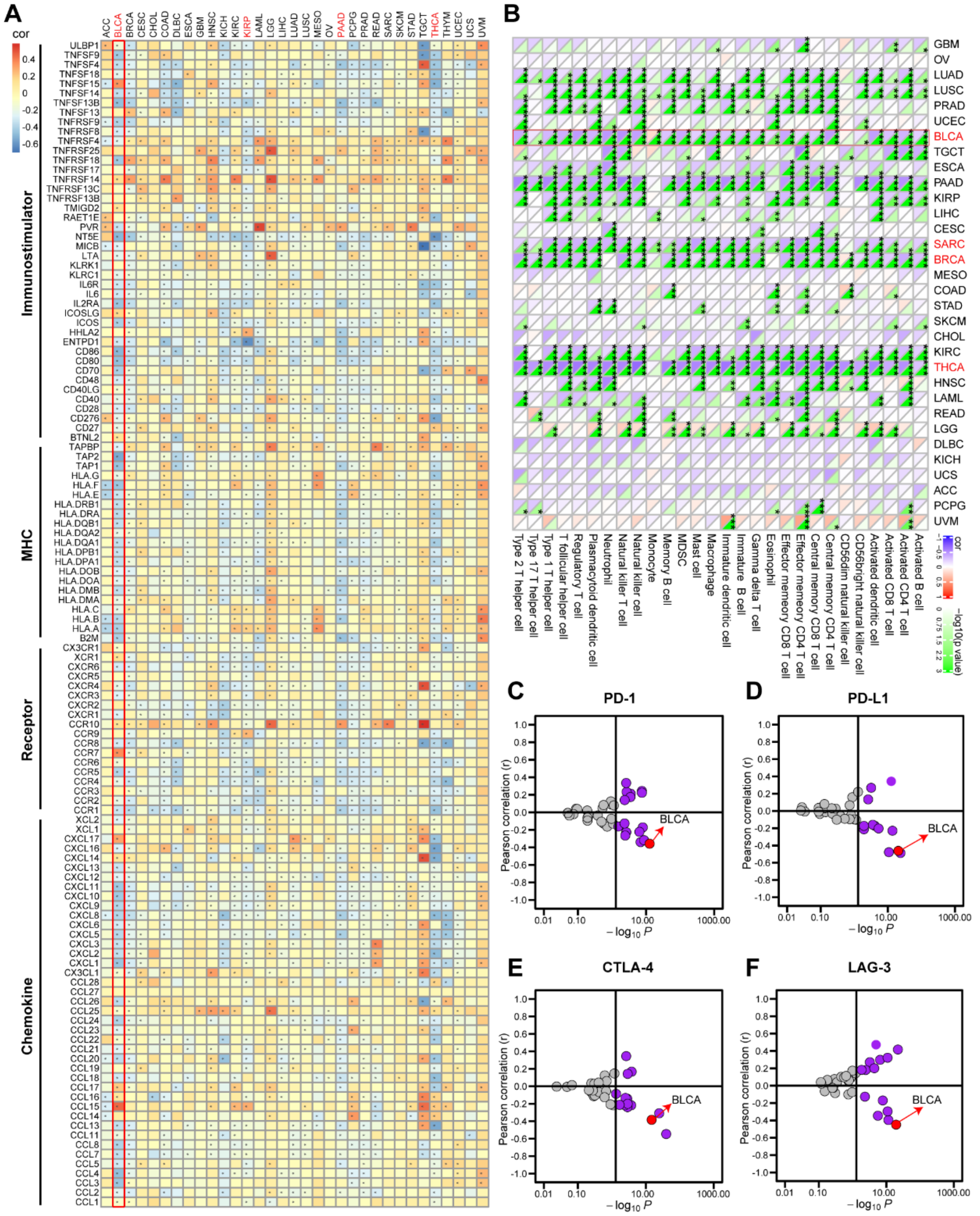

Supplementary Fig 2. Pan-cancer analysis of immunosuppressive role of BCAT2. (A) Correlation analyses between BCAT2 and immunological related indicators in 33 types of cancer. Grid color represents positive or negative correlation. *P< 0.05. (B) Correlation analyses between BCAT2 and TIICs in 33 types of cancer. Grid color represents positive or negative correlation. *P< 0.05, **P< 0.01, ***P< 0.001. Correlation analyses between BCAT2 and effector genes of ICB (C) PD-1 (D) PD-L1 (E) CTLA-4 (F) LAG-3 in 33 types of cancer. Purple circle means correlation with significance and grey circle means correlation with no significance. Circle in the upper

half of square represents positive relationship and circle in the bottom half square represents negative relationship. Red arrow points to BLCA.

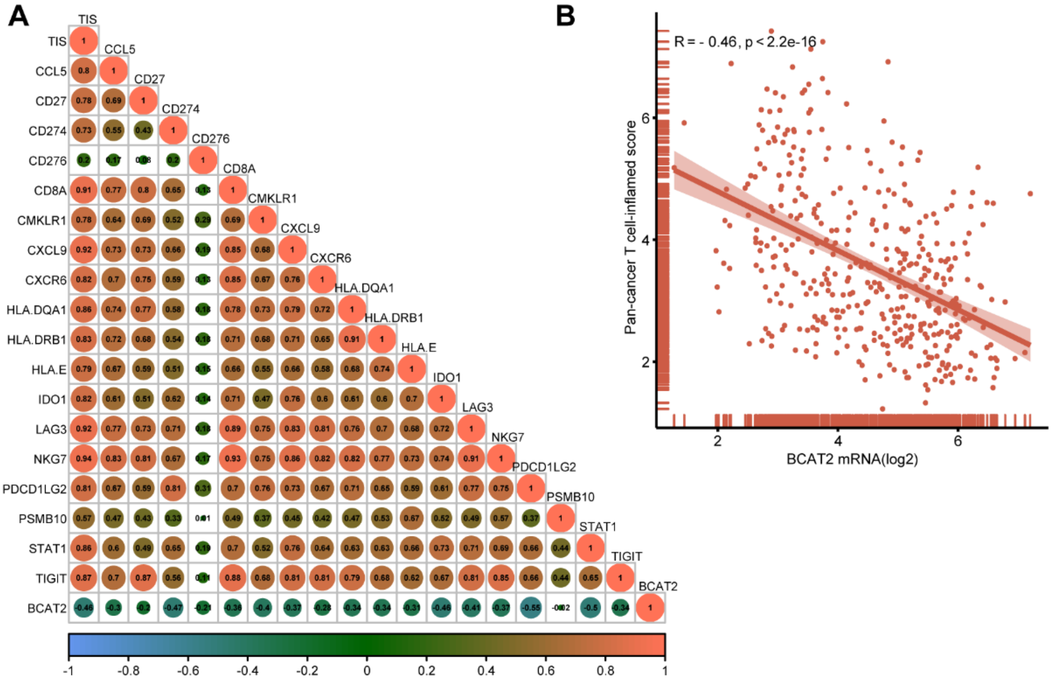

Supplementary Fig 3. Correlation between BCAT2 and TIS. (A) Correlation between BCAT2 and effector genes of TIS in BLCA. (B) Correlation between BCAT2 and TIS in pan-cancer analysis.

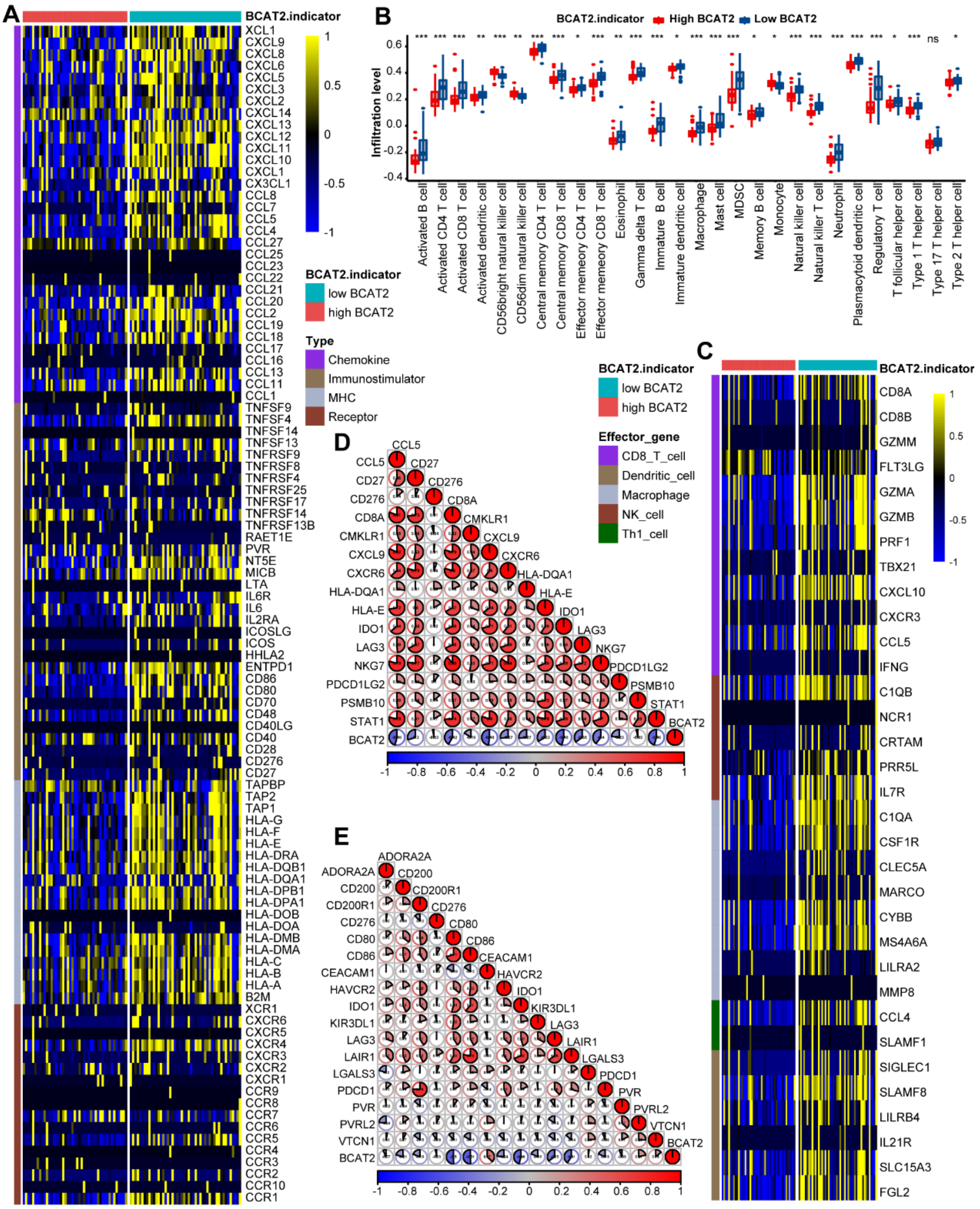
A
BCAT2.indicator
BCAT2.indicator
low BCAT2
high BCAT2
Type
Chemokine
Immunostimulator
MHC
Receptor
B
BCAT2.indicator High BCAT2 Low BCAT2
Infiltration level
C
BCAT2.indicator
Effector_gene
CD8_T_cell
Dendritic_cell
Macrophage
NK_cell
Th1_cell
D
E

GSE31684

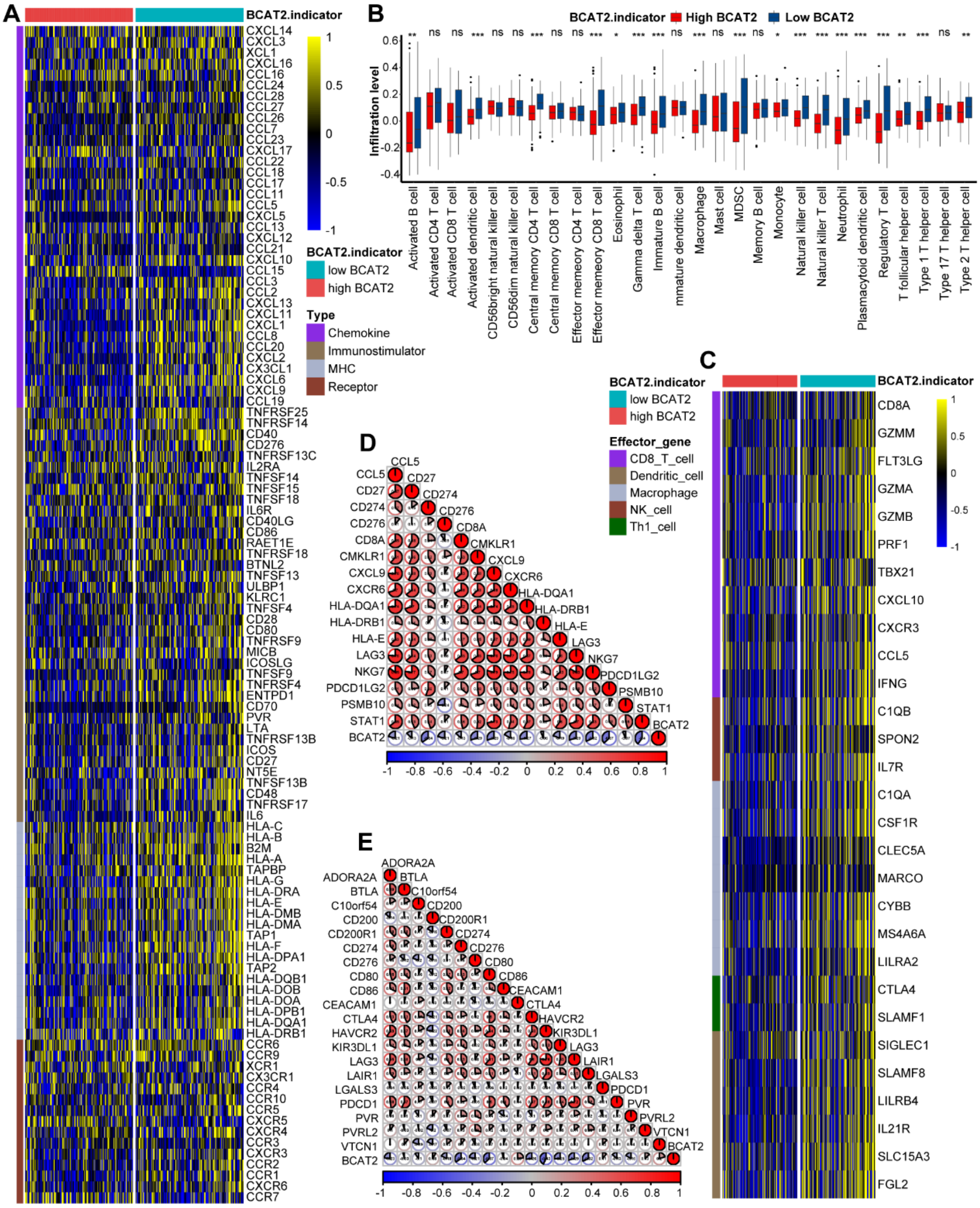
A
BCAT2.indicator
CXCL14
CXCL3
XCL1
CXCL16
CCL16
CCL24
CCL28
CCL27
CCL26
CCL7
CCL23
CXCL17
CCL22
CCL18
CCL17
CCL11
CCL5
CXCL5
CCL13
CXCL12
CCL21
CXCL10
CCL15
CCL3
CCL2
CXCL13
CXCL11
CXCL1
CCL8
CCL20
CXCL2
CX3CL1
CXCL6
CXCL9
CCL19
TNFRSF25
TNFRSF14
CD40
CD276
TNFRSF13C
IL2RA
TNFSF14
TNFSF15
TNFSF18
IL6R
CD40LG
CD86
RAET1E
TNFRSF18
BTNL2
TNFSF13
ULBP1
KLRC1
TNFSF4
CD28
CD80
TNFRSF9
MICB
ICOSLG
TNFSF9
TNFRSF4
ENTPD1
CD70
PVR
LTA
TNFRSF13B
ICOS
CD27
NT5E
TNFSF13B
CD48
TNFRSF17
IL6
HLA-C
HLA-B
B2M
HLA-A
TAPBP
HLA-G
HLA-DRA
HLA-E
HLA-DMB
HLA-DMA
TAP1
HLA-F
HLA-DPA1
TAP2
HLA-DQB1
HLA-DOB
HLA-DOA
HLA-DPB1
HLA-DQA1
HLA-DRB1
CCR6
CCR9
XCR1
CX3CR1
CCR4
CCR10
CCR5
CXCR5
CXCR4
CCR3
CXCR3
CCR2
CCR1
CXCR6
CCR7
1
0.5
0
-0.5
-1
BCAT2.indicator
low BCAT2
high BCAT2
Type
Chemokine
Immunostimulator
MHC
Receptor
B
BCAT2.indicator High BCAT2 Low BCAT2
Infiltration level
0.6
0.4
0.2
0.0
-0.2
-0.4
** ns ns *** ns ns *** ns ns *** * *** *** ns *** ns *** ns * *** *** *** *** *** ** *** ns **
Activated B cell
Activated CD4 T cell
Activated CD8 T cell
Activated dendritic cell
CD56bright natural killer cell
CD56dim natural killer cell
Central memory CD4 T cell
Central memory CD8 T cell
Effector memeory CD4 T cell
Effector memeory CD8 T cell
Eosinophil
Gamma delta T cell
Immature B cell
mmature dendritic cell
Macrophage
Mast cell
MDSC
Memory B cell
Monocyte
Natural killer cell
Natural killer T cell
Neutrophil
Plasmacytoid dendritic cell
Regulatory T cell
T follicular helper cell
Type 1 T helper cell
Type 17 T helper cell
Type 2 T helper cell
C
BCAT2.indicator
low BCAT2
high BCAT2
Effector_gene
CD8_T_cell
Dendritic_cell
Macrophage
NK_cell
Th1_cell
BCAT2.indicator
CD8A
GZMM
FLT3LG
GZMA
GZMB
PRF1
TBX21
CXCL10
CXCR3
CCL5
IFNG
C1QB
SPON2
IL7R
C1QA
CSF1R
CLEC5A
MARCO
CYBB
MS4A6A
LILRA2
CTLA4
SLAMF1
SIGLEC1
SLAMF8
LILRB4
IL21R
SLC15A3
FGL2
1
0.5
0
-0.5
-1
D
CCL5
CD27
CD274
CD276
CD8A
CMKLR1
CXCL9
CXCR6
HLA-DQA1
HLA-DRB1
HLA-E
LAG3
NKG7
PDCD1LG2
PSMB10
STAT1
BCAT2
-1 -0.8 -0.6 -0.4 -0.2 0 0.2 0.4 0.6 0.8 1
E
ADORA2A
BTLA
C10orf54
CD200
CD200R1
CD274
CD276
CD80
CD86
CEACAM1
CTLA4
HAVCR2
KIR3DL1
LAG3
LAIR1
LGALS3
PDCD1
PVR
PVRL2
VTCN1
BCAT2
-1 -0.8 -0.6 -0.4 -0.2 0 0.2 0.4 0.6 0.8 1

GSE32894

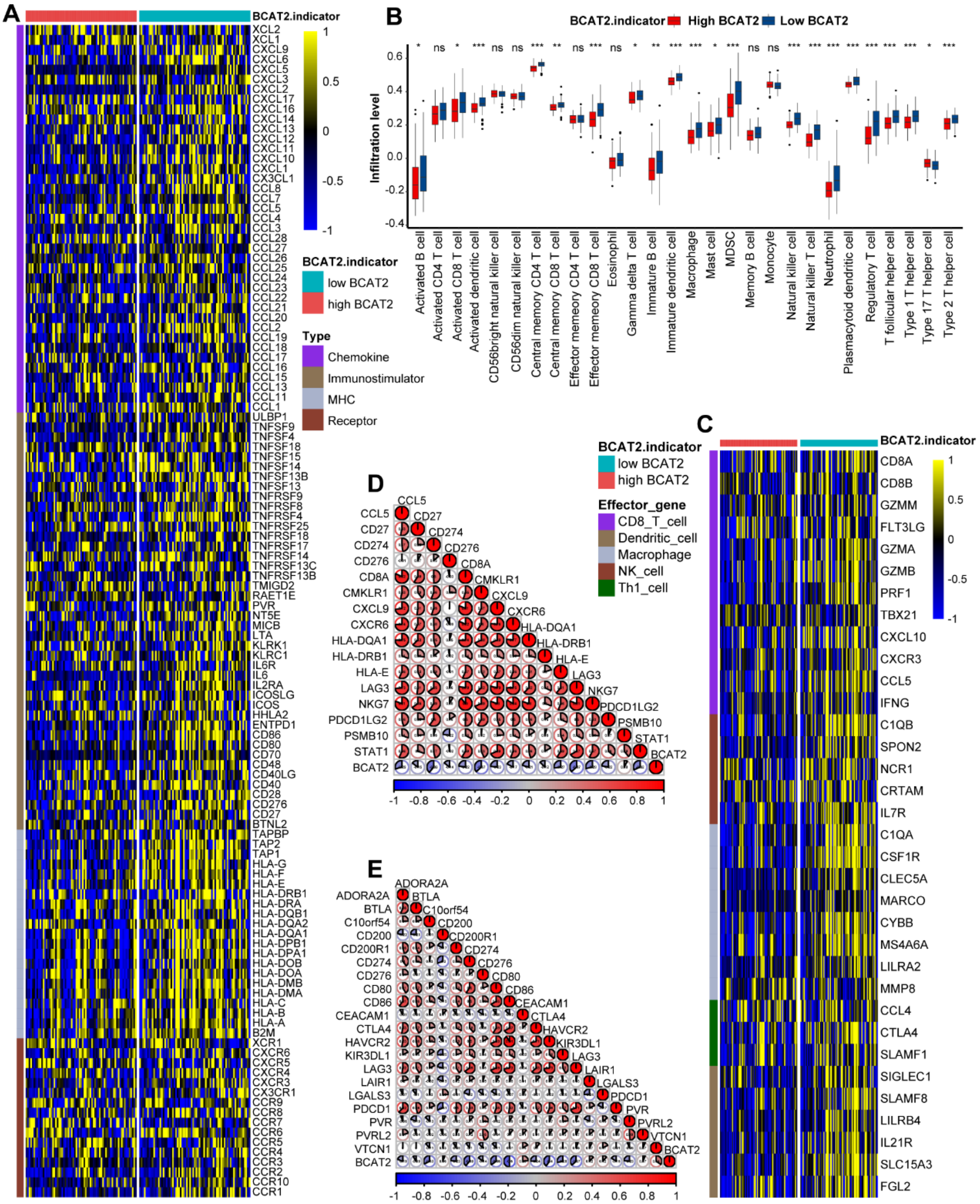

A
BCAT2.indicator
XCL2
XCL1
CXCL9
CXCL6
CXCL5
CXCL3
CXCL2
CXCL17
CXCL16
CXCL14
CXCL13
CXCL12
CXCL11
CXCL10
CXCL1
CX3CL1
CCL8
CCL7
CCL5
CCL4
CCL3
CCL28
CCL27
CCL26
CCL25
CCL24
CCL23
CCL22
CCL21
CCL20
CCL2
CCL19
CCL18
CCL17
CCL16
CCL15
CCL13
CCL11
CCL1
ULBP1
TNFSF9
TNFSF4
TNFSF18
TNFSF15
TNFSF14
TNFSF13B
TNFSF13
TNFRSF9
TNFRSF8
TNFRSF4
TNFRSF25
TNFRSF18
TNFRSF17
TNFRSF14
TNFRSF13C
TNFRSF13B
TMIGD2
RAET1E
PVR
NT5E
MICB
LTA
KLRK1
KLRC1
IL6R
IL6
IL2RA
ICOSLG
ICOS
HHLA2
ENTPD1
CD86
CD80
CD70
CD48
CD40LG
CD40
CD28
CD276
CD27
BTNL2
TAPBP
TAP2
TAP1
HLA-G
HLA-F
HLA-E
HLA-DRB1
HLA-DRA
HLA-DQB1
HLA-DQA2
HLA-DQA1
HLA-DPB1
HLA-DPA1
HLA-DOB
HLA-DOA
HLA-DMB
HLA-DMA
HLA-C
HLA-B
HLA-A
B2M
XCR1
CXCR6
CXCR5
CXCR4
CXCR3
CX3CR1
CCR9
CCR8
CCR7
CCR6
CCR5
CCR4
CCR3
CCR2
CCR10
CCR1
1
0.5
0
-0.5
-1
BCAT2.indicator
low BCAT2
high BCAT2
Type
Chemokine
Immunostimulator
MHC
Receptor
B
BCAT2.indicator High BCAT2 Low BCAT2
Infiltration level
0.6
0.4
0.2
0.0
-0.2
-0.4
* ns * *** ns ns *** ** ns *** ns * ** *** *** * *** ns ns *** *** *** *** *** *** *** * ***
Activated B cell
Activated CD4 T cell
Activated CD8 T cell
Activated dendritic cell
CD56bright natural killer cell
CD56dim natural killer cell
Central memory CD4 T cell
Central memory CD8 T cell
Effector memeory CD4 T cell
Effector memeory CD8 T cell
Eosinophil
Gamma delta T cell
Immature B cell
Immature dendritic cell
Macrophage
Mast cell
MDSC
Memory B cell
Monocyte
Natural killer cell
Natural killer T cell
Neutrophil
Plasmacytoid dendritic cell
Regulatory T cell
T follicular helper cell
Type 1 T helper cell
Type 17 T helper cell
Type 2 T helper cell
C
BCAT2.indicator
CD8A
CD8B
GZMM
FLT3LG
GZMA
GZMB
PRF1
TBX21
CXCL10
CXCR3
CCL5
IFNG
C1QB
SPON2
NCR1
CRTAM
IL7R
C1QA
CSF1R
CLEC5A
MARCO
CYBB
MS4A6A
LILRA2
MMP8
CCL4
CTLA4
SLAMF1
SIGLEC1
SLAMF8
LILRB4
IL21R
SLC15A3
FGL2
1
0.5
0
-0.5
-1
BCAT2.indicator
low BCAT2
high BCAT2
Effector_gene
CD8_T_cell
Dendritic_cell
Macrophage
NK_cell
Th1_cell
D
CCL5
CD27
CD274
CD276
CD8A
CMKLR1
CXCL9
CXCR6
HLA-DQA1
HLA-DRB1
HLA-E
LAG3
NKG7
PDCD1LG2
PSMB10
STAT1
BCAT2
-1 -0.8 -0.6 -0.4 -0.2 0 0.2 0.4 0.6 0.8 1
E
ADORA2A
BTLA
C10orf54
CD200
CD200R1
CD274
CD276
CD80
CD86
CEACAM1
CTLA4
HAVCR2
KIR3DL1
LAG3
LAIR1
LGALS3
PDCD1
PVR
PVRL2
VTCN1
BCAT2
-1 -0.8 -0.6 -0.4 -0.2 0 0.2 0.4 0.6 0.8 1

GSE48075

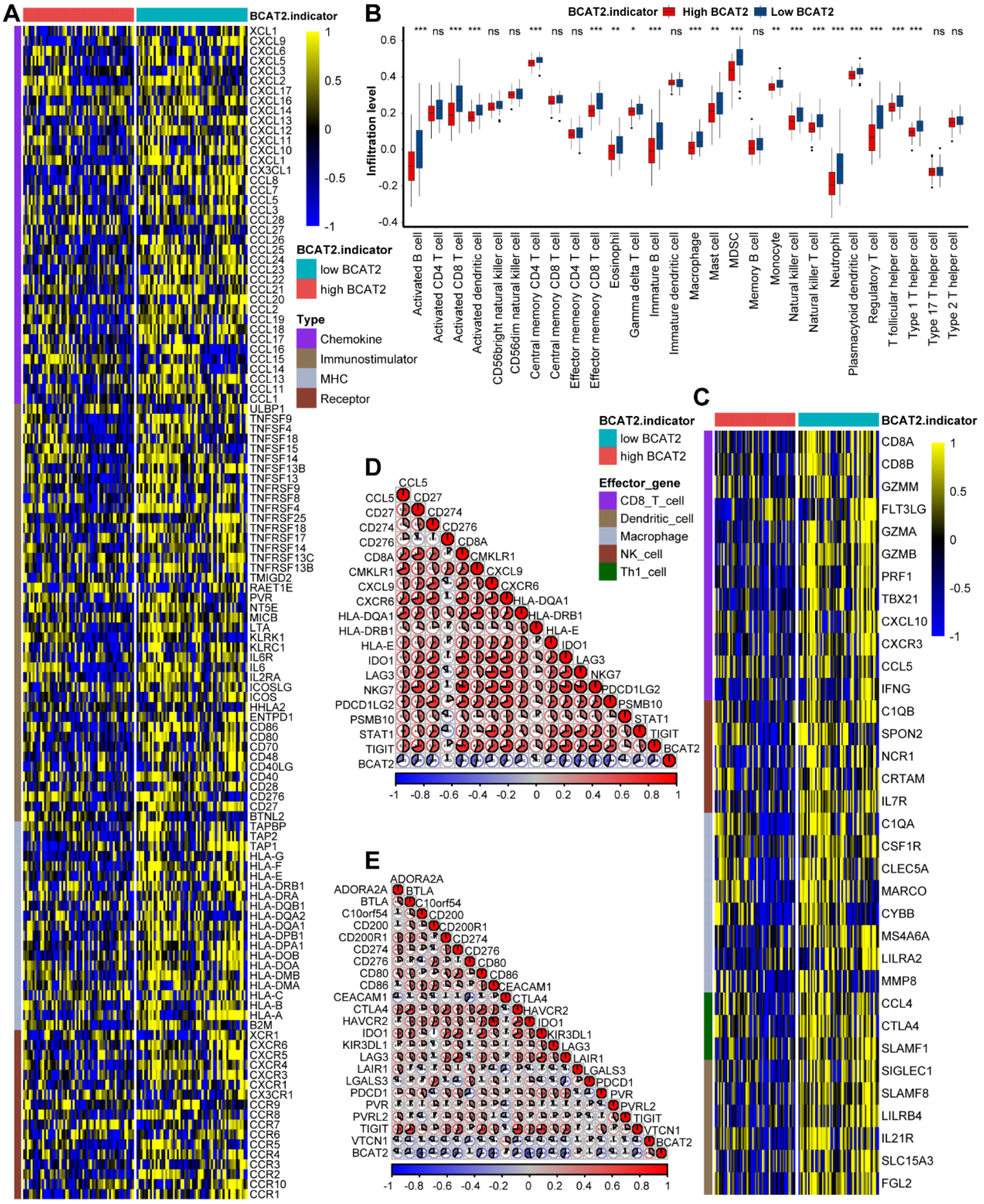
A
BCAT2.indicator
XCL1
CXCL9
CXCL6
CXCL5
CXCL3
CXCL2
CXCL17
CXCL16
CXCL14
CXCL13
CXCL12
CXCL11
CXCL10
CXCL1
CX3CL1
CCL8
CCL7
CCL5
CCL3
CCL28
CCL27
CCL26
CCL25
CCL24
CCL23
CCL22
CCL21
CCL20
CCL2
CCL19
CCL18
CCL17
CCL16
CCL15
CCL14
CCL13
CCL11
CCL1
ULBP1
TNFSF9
TNFSF4
TNFSF18
TNFSF15
TNFSF14
TNFSF13B
TNFSF13
TNFRSF9
TNFRSF8
TNFRSF4
TNFRSF25
TNFRSF18
TNFRSF17
TNFRSF14
TNFRSF13C
TNFRSF13B
TMIGD2
RAET1E
PVR
NT5E
MICB
LTA
KLRK1
KLRC1
IL6R
IL6
IL2RA
ICOSLG
ICOS
HHLA2
ENTPD1
CD86
CD80
CD70
CD48
CD40LG
CD40
CD28
CD276
CD27
BTNL2
TAPBP
TAP2
TAP1
HLA-G
HLA-F
HLA-E
HLA-DRB1
HLA-DRA
HLA-DQB1
HLA-DQA2
HLA-DQA1
HLA-DPB1
HLA-DPA1
HLA-DOB
HLA-DOA
HLA-DMB
HLA-DMA
HLA-C
HLA-B
HLA-A
B2M
XCR1
CXCR6
CXCR5
CXCR4
CXCR3
CXCR1
CX3CR1
CCR9
CCR8
CCR7
CCR6
CCR5
CCR4
CCR3
CCR2
CCR10
CCR1
1
0.5
0
-0.5
-1
BCAT2.indicator
low BCAT2
high BCAT2
Type
Chemokine
Immunostimulator
MHC
Receptor
B
BCAT2.indicator High BCAT2 Low BCAT2
Infiltration level
0.6
0.4
0.2
0.0
-0.2
-0.4
*** ns *** *** ns ns *** ns ns *** ** * *** ns *** ** *** ns ** *** *** *** *** *** *** *** ns ns
Activated B cell
Activated CD4 T cell
Activated CD8 T cell
Activated dendritic cell
CD56bright natural killer cell
CD56dim natural killer cell
Central memory CD4 T cell
Central memory CD8 T cell
Effector memeory CD4 T cell
Effector memeory CD8 T cell
Eosinophil
Gamma delta T cell
Immature B cell
Immature dendritic cell
Macrophage
Mast cell
MDSC
Memory B cell
Monocyte
Natural killer cell
Natural killer T cell
Neutrophil
Plasmacytoid dendritic cell
Regulatory T cell
T follicular helper cell
Type 1 T helper cell
Type 17 T helper cell
Type 2 T helper cell
C
BCAT2.indicator
low BCAT2
high BCAT2
Effector_gene
CD8_T_cell
Dendritic_cell
Macrophage
NK_cell
Th1_cell
BCAT2.indicator
CD8A
CD8B
GZMM
FLT3LG
GZMA
GZMB
PRF1
TBX21
CXCL10
CXCR3
CCL5
IFNG
C1QB
SPON2
NCR1
CRTAM
IL7R
C1QA
CSF1R
CLEC5A
MARCO
CYBB
MS4A6A
LILRA2
MMP8
CCL4
CTLA4
SLAMF1
SIGLEC1
SLAMF8
LILRB4
IL21R
SLC15A3
FGL2
1
0.5
0
-0.5
-1
D
CCL5
CD27
CD274
CD276
CD8A
CMKLR1
CXCL9
CXCR6
HLA-DQA1
HLA-DRB1
HLA-E
IDO1
LAG3
NKG7
PDCD1LG2
PSMB10
STAT1
TIGIT
BCAT2
-1 -0.8 -0.6 -0.4 -0.2 0 0.2 0.4 0.6 0.8 1
E
ADORA2A
BTLA
C10orf54
CD200
CD200R1
CD274
CD276
CD80
CD86
CEACAM1
CTLA4
HAVCR2
IDO1
KIR3DL1
LAG3
LAIR1
LGALS3
PDCD1
PVR
PVRL2
TIGIT
VTCN1
BCAT2
-1 -0.8 -0.6 -0.4 -0.2 0 0.2 0.4 0.6 0.8 1

GSE48276

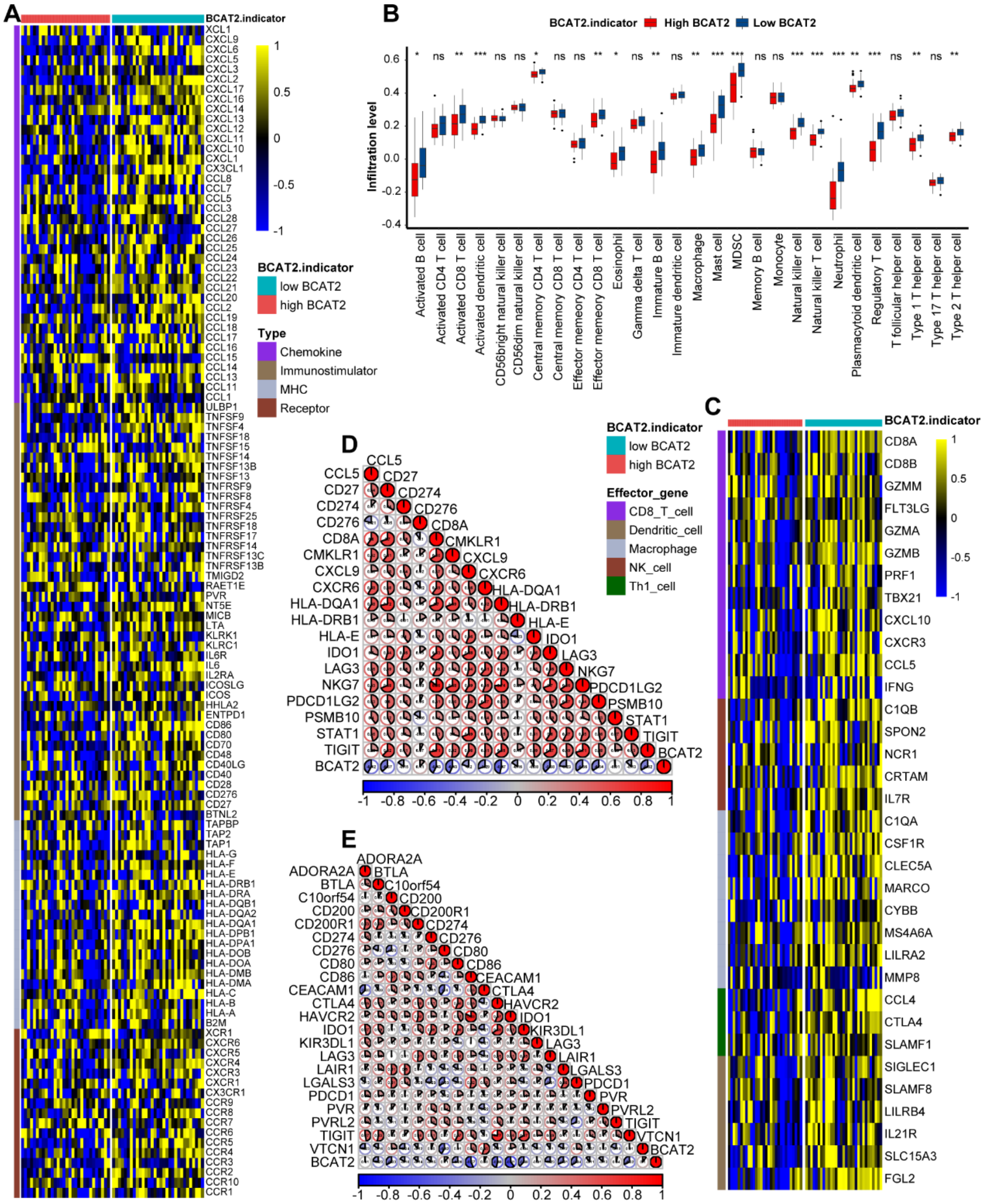
A
BCAT2.indicator
Type
Chemokine
Immunostimulator
MHC
Receptor
B
High BCAT2
Low BCAT2
Infiltration level
C
Effector_gene
CD8_T_cell
Dendritic_cell
Macrophage
NK_cell
Th1_cell
D
E

GSE69795

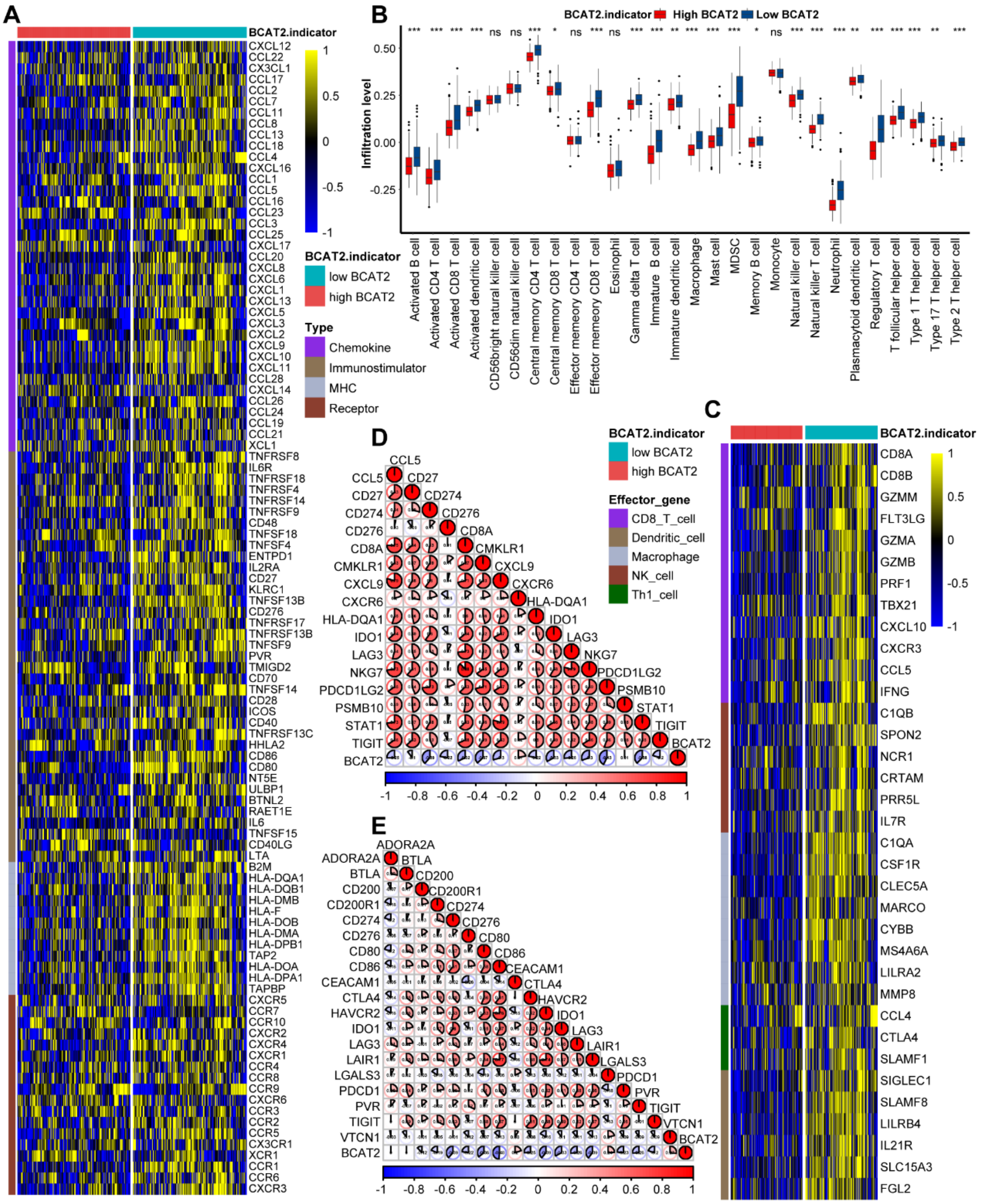
A
B
C
D
E
BCAT2.indicator
low BCAT2
high BCAT2
Type
Chemokine
Immunostimulator
MHC
Receptor
BCAT2.indicator High BCAT2 Low BCAT2
Infiltration level
Effector_gene
CD8_T_cell
Dendritic_cell
Macrophage
NK_cell
Th1_cell

GSE83586

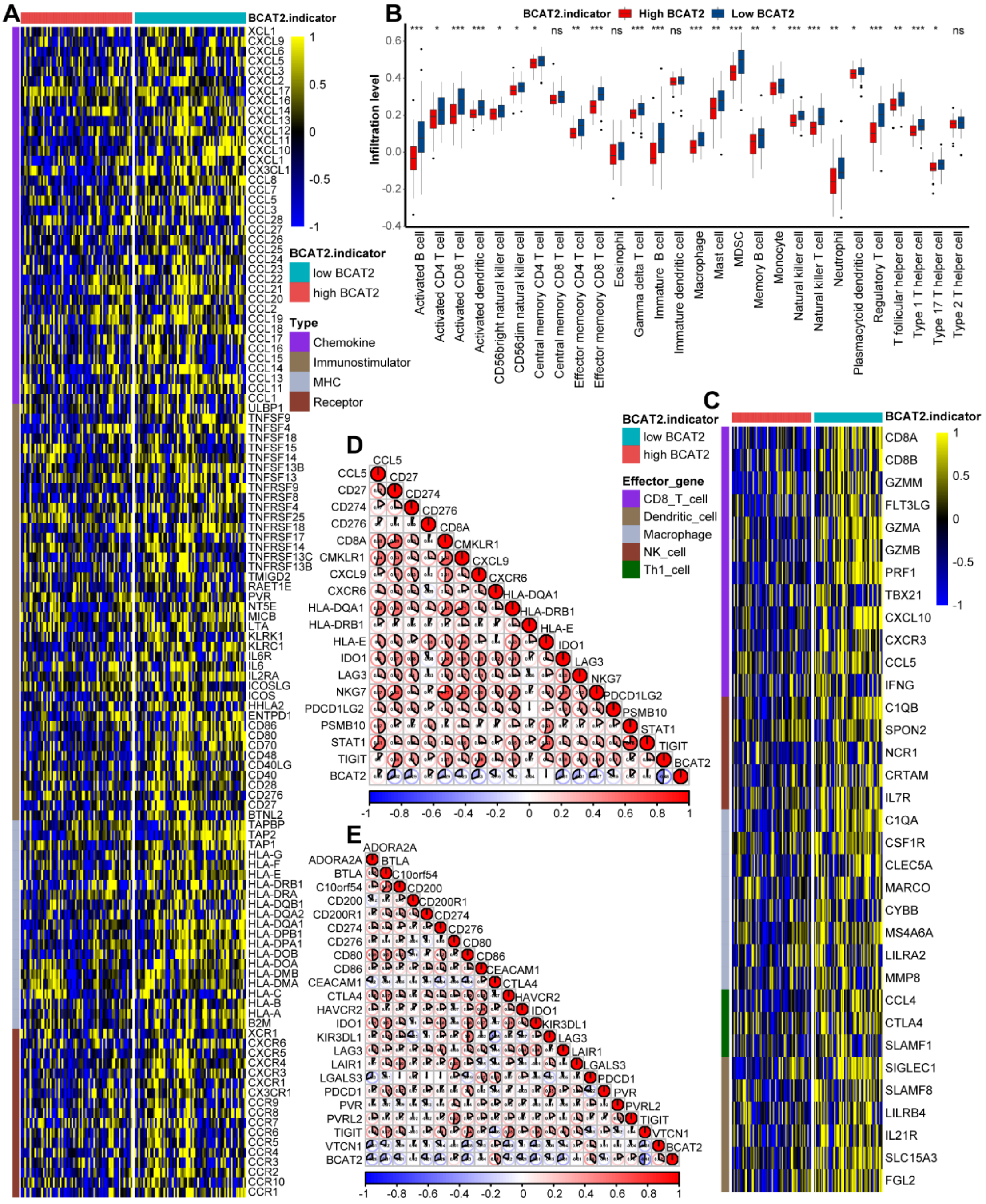
A
BCAT2.indicator
XCL1
CXCL9
CXCL6
CXCL5
CXCL3
CXCL2
CXCL17
CXCL16
CXCL14
CXCL13
CXCL12
CXCL11
CXCL10
CXCL1
CX3CL1
CCL8
CCL7
CCL5
CCL3
CCL28
CCL27
CCL26
CCL25
CCL24
CCL23
CCL22
CCL21
CCL20
CCL2
CCL19
CCL18
CCL17
CCL16
CCL15
CCL14
CCL13
CCL11
CCL1
ULBP1
TNFSF9
TNFSF4
TNFSF18
TNFSF15
TNFSF14
TNFSF13B
TNFSF13
TNFRSF9
TNFRSF8
TNFRSF4
TNFRSF25
TNFRSF18
TNFRSF17
TNFRSF14
TNFRSF13C
TNFRSF13B
TMIGD2
RAET1E
PVR
NT5E
MICB
LTA
KLRK1
KLRC1
IL6R
IL6
IL2RA
ICOSLG
ICOS
HHLA2
ENTPD1
CD86
CD80
CD70
CD48
CD40LG
CD40
CD28
CD276
CD27
BTNL2
TAPBP
TAP2
TAP1
HLA-G
HLA-F
HLA-E
HLA-DRB1
HLA-DRA
HLA-DQB1
HLA-DQA2
HLA-DQA1
HLA-DPB1
HLA-DPA1
HLA-DOB
HLA-DOA
HLA-DMB
HLA-DMA
HLA-C
HLA-B
HLA-A
B2M
XCR1
CXCR6
CXCR5
CXCR4
CXCR3
CXCR1
CX3CR1
CCR9
CCR8
CCR7
CCR6
CCR5
CCR4
CCR3
CCR2
CCR10
CCR1
1
0.5
0
-0.5
-1
BCAT2.indicator
low BCAT2
high BCAT2
Type
Chemokine
Immunostimulator
MHC
Receptor
B
BCAT2.indicator High BCAT2 Low BCAT2
Infiltration level
0.6
0.4
0.2
0.0
-0.2
-0.4
*** * *** *** * * * ns ** *** ns *** *** ns *** ** *** ** * *** *** ** * *** ** *** * ns
Activated B cell
Activated CD4 T cell
Activated CD8 T cell
Activated dendritic cell
CD56bright natural killer cell
CD56dim natural killer cell
Central memory CD4 T cell
Central memory CD8 T cell
Effector memeory CD4 T cell
Effector memeory CD8 T cell
Eosinophil
Gamma delta T cell
Immature B cell
Immature dendritic cell
Macrophage
Mast cell
MDSC
Memory B cell
Monocyte
Natural killer cell
Natural killer T cell
Neutrophil
Plasmacytoid dendritic cell
Regulatory T cell
T follicular helper cell
Type 1 T helper cell
Type 17 T helper cell
Type 2 T helper cell
C
BCAT2.indicator
low BCAT2
high BCAT2
Effector_gene
CD8_T_cell
Dendritic_cell
Macrophage
NK_cell
Th1_cell
BCAT2.indicator
CD8A
CD8B
GZMM
FLT3LG
GZMA
GZMB
PRF1
TBX21
CXCL10
CXCR3
CCL5
IFNG
C1QB
SPON2
NCR1
CRTAM
IL7R
C1QA
CSF1R
CLEC5A
MARCO
CYBB
MS4A6A
LILRA2
MMP8
CCL4
CTLA4
SLAMF1
SIGLEC1
SLAMF8
LILRB4
IL21R
SLC15A3
FGL2
1
0.5
0
-0.5
-1
D
CCL5
CD27
CD274
CD276
CD8A
CMKLR1
CXCL9
CXCR6
HLA-DQA1
HLA-DRB1
HLA-E
IDO1
LAG3
NKG7
PDCD1LG2
PSMB10
STAT1
TIGIT
BCAT2
-1 -0.8 -0.6 -0.4 -0.2 0 0.2 0.4 0.6 0.8 1
E
ADORA2A
BTLA
C10orf54
CD200
CD200R1
CD274
CD276
CD80
CD86
CEACAM1
CTLA4
HAVCR2
IDO1
KIR3DL1
LAG3
LAIR1
LGALS3
PDCD1
PVR
PVRL2
TIGIT
VTCN1
BCAT2
-1 -0.8 -0.6 -0.4 -0.2 0 0.2 0.4 0.6 0.8 1

GSE86411

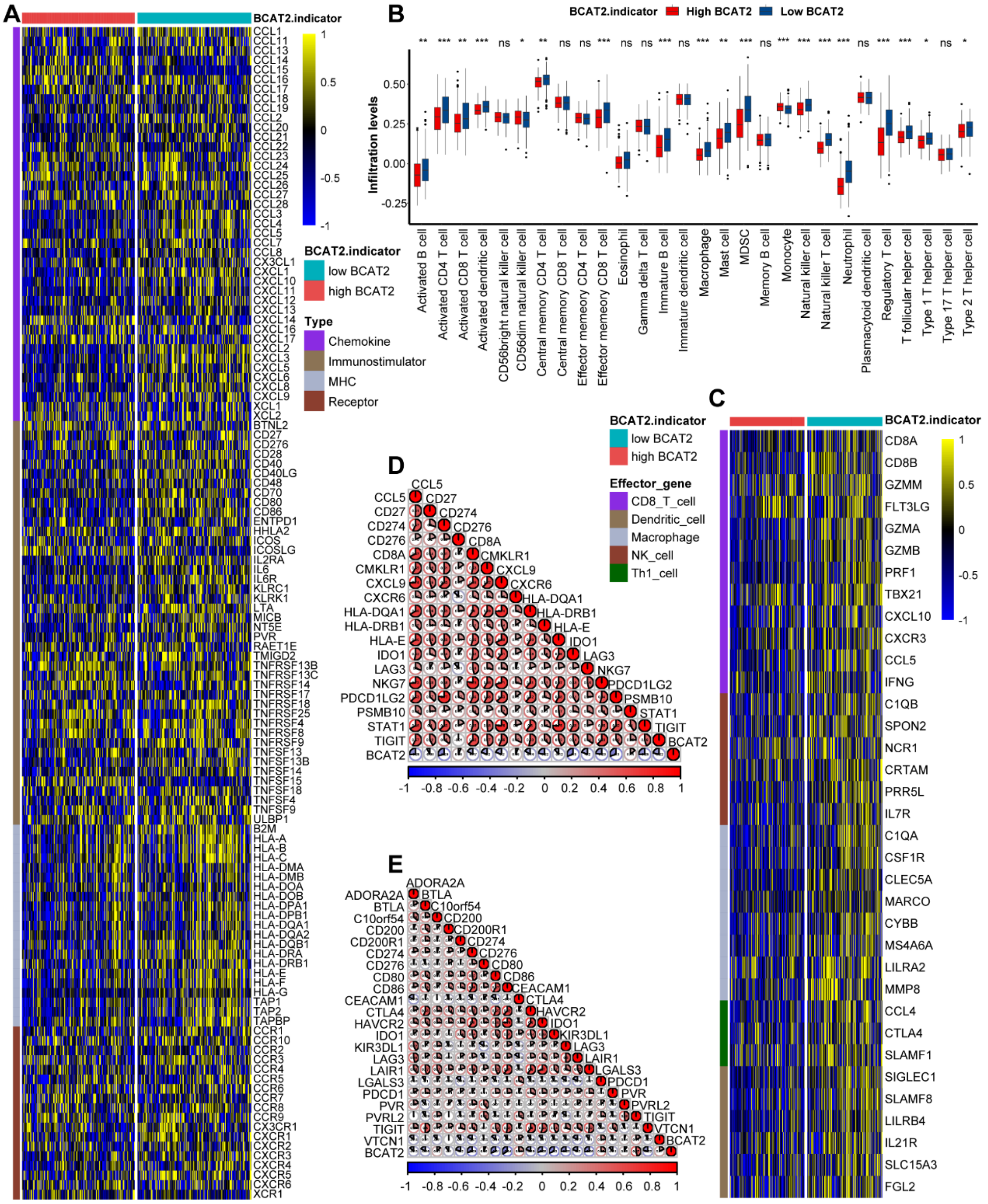

GSE87304

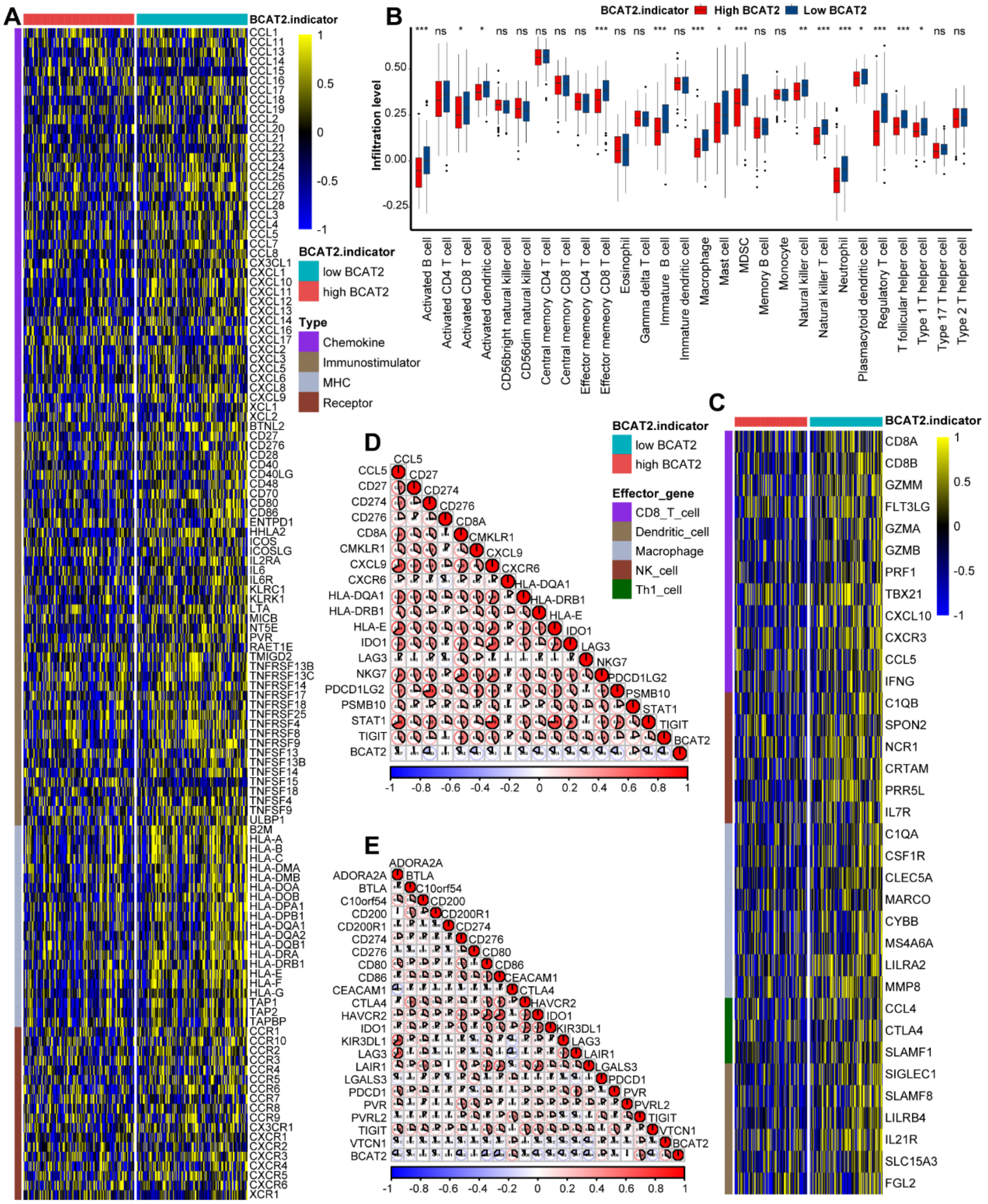

GSE128702

Supplementary Fig 4-12. Validation of immunosuppressive role of BCAT2 in nine BLCA cohorts (GSE31684, GSE32894, GSE48075, GSE48276, GSE69795, GSE83586, GSE86411, GSE87304 and GSE128702). (A) Expression patterns of chemokines, chemokine receptors, MHC effector genes and immunostimulators in high and low BCAT2 groups (B) Infiltration levels of TIICs in high and low BCAT2 groups (C) Effector genes of multiple subtypes of immune cell (CD8$^+$ T cell, DC, Macrophage, NK and Th1) in high and low BCAT2 groups (D) Correlation between BCAT2 and TIS

score (E) Correlation between BCAT2 and inhibitory immune checkpoints. Number in the circle means correlation coefficient. *P< 0.05, **P< 0.01, ***P< 0.001, ns: no significance.

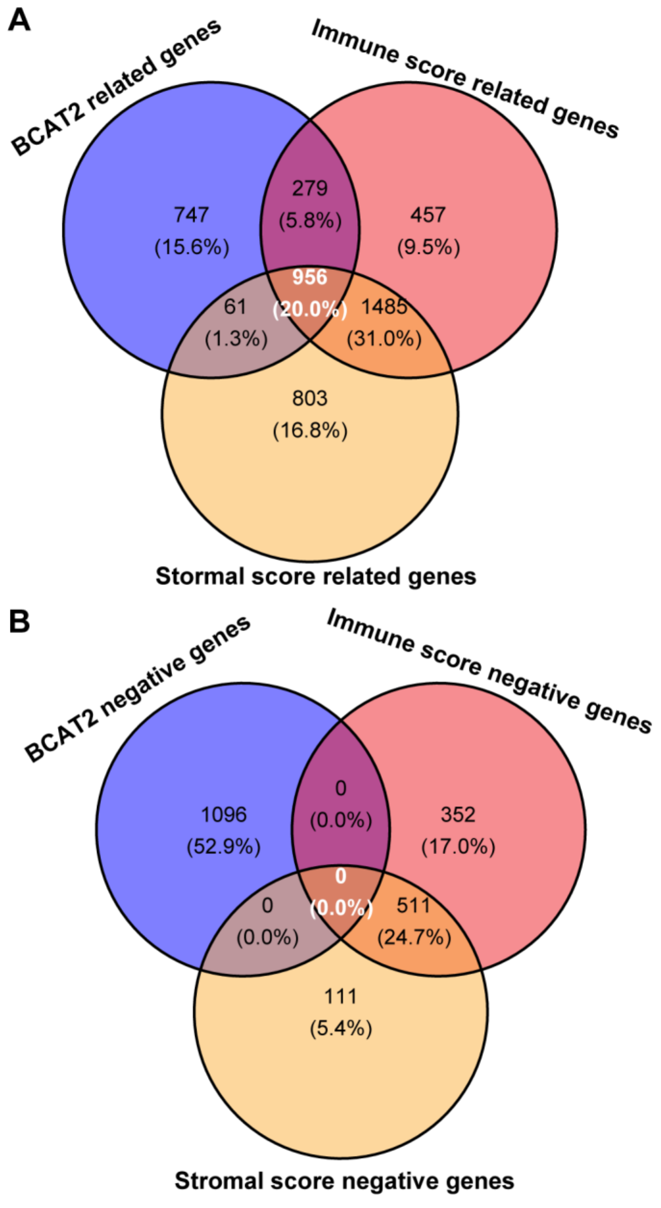

Supplementary Fig 13. Overlap of BCAT2, immune score and stromal score related genes. (A) Intersection of BCAT2, immune score and stromal score related genes. (B) Intersection of BCAT2, immune score and stromal score negative-related genes.

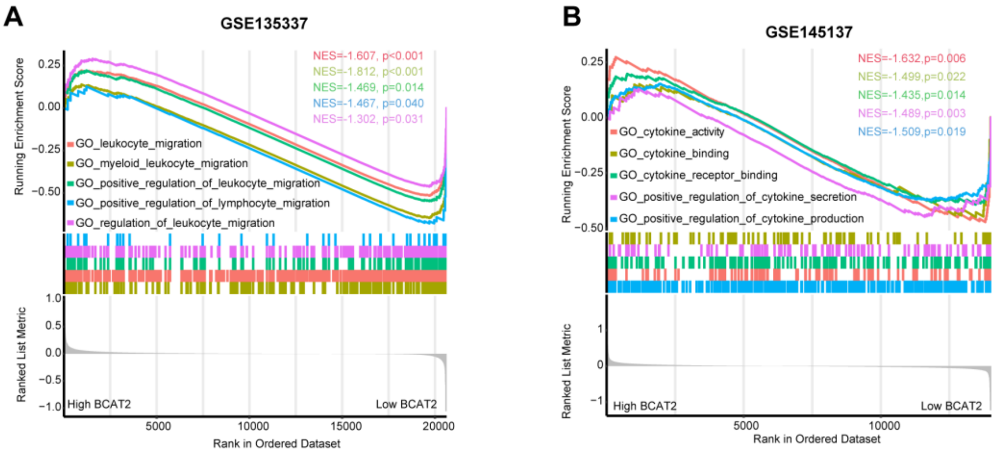

Supplementary Fig 14. GSEA analyses of differences on activity of TME related pathways between high and low BCAT2 groups by scRNA-seq. (A) GSEA analyses of GO terms indicate different activities of immune cell migration related pathways between high and low BCAT2 groups in GSE135337 scRNA cohort (B) GSEA analyses of GO terms indicate different activities of cytokine related pathways between high and low BCAT2 groups in GSE145137 scRNA cohort.

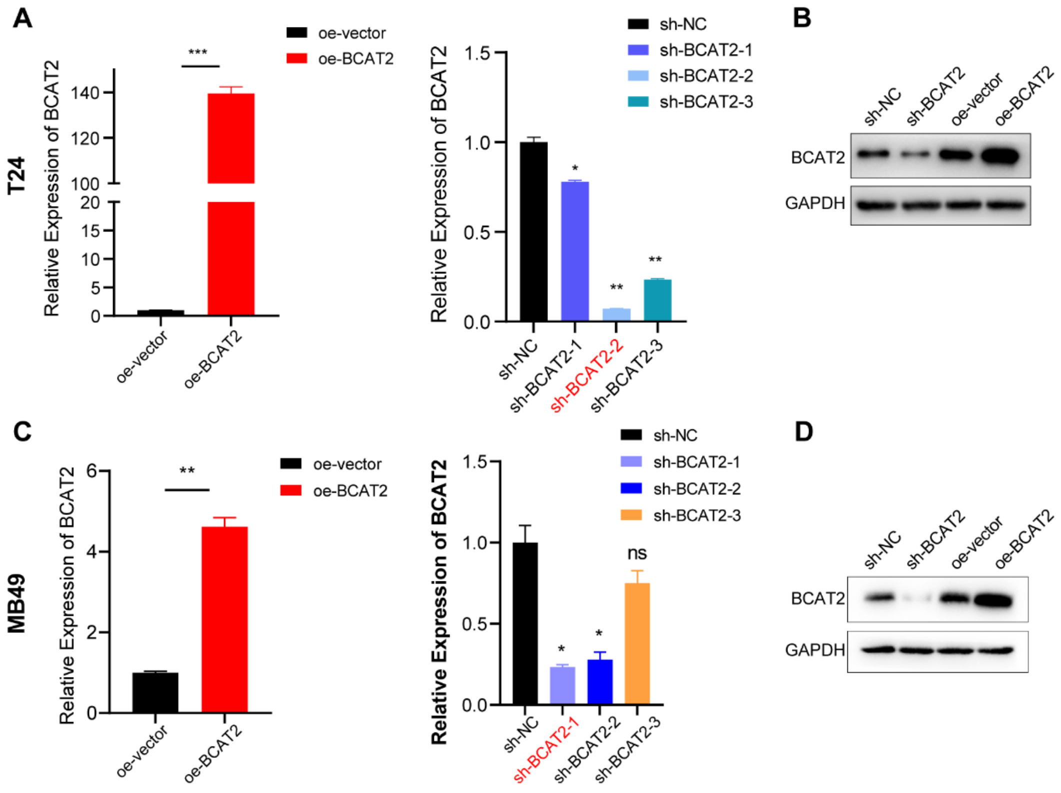

Supplementary Fig 15. Validation of BCAT2 OE and BCAT2 KD cell lines. (A) Validation of BCAT2 expression level in T24 cell line using qRT-PCR. *P<0.05, **P< 0.01, ***P<0.001. (B) Validation of BCAT2 expression level in T24 cell line using Western blot. (C) Validation of BCAT2 expression level in MB49 cell line using qRT-PCR. *P<0.05, **P< 0.01, ns: no significance. (D) Validation of BCAT2 expression level in MB49 cell line using Western blot.

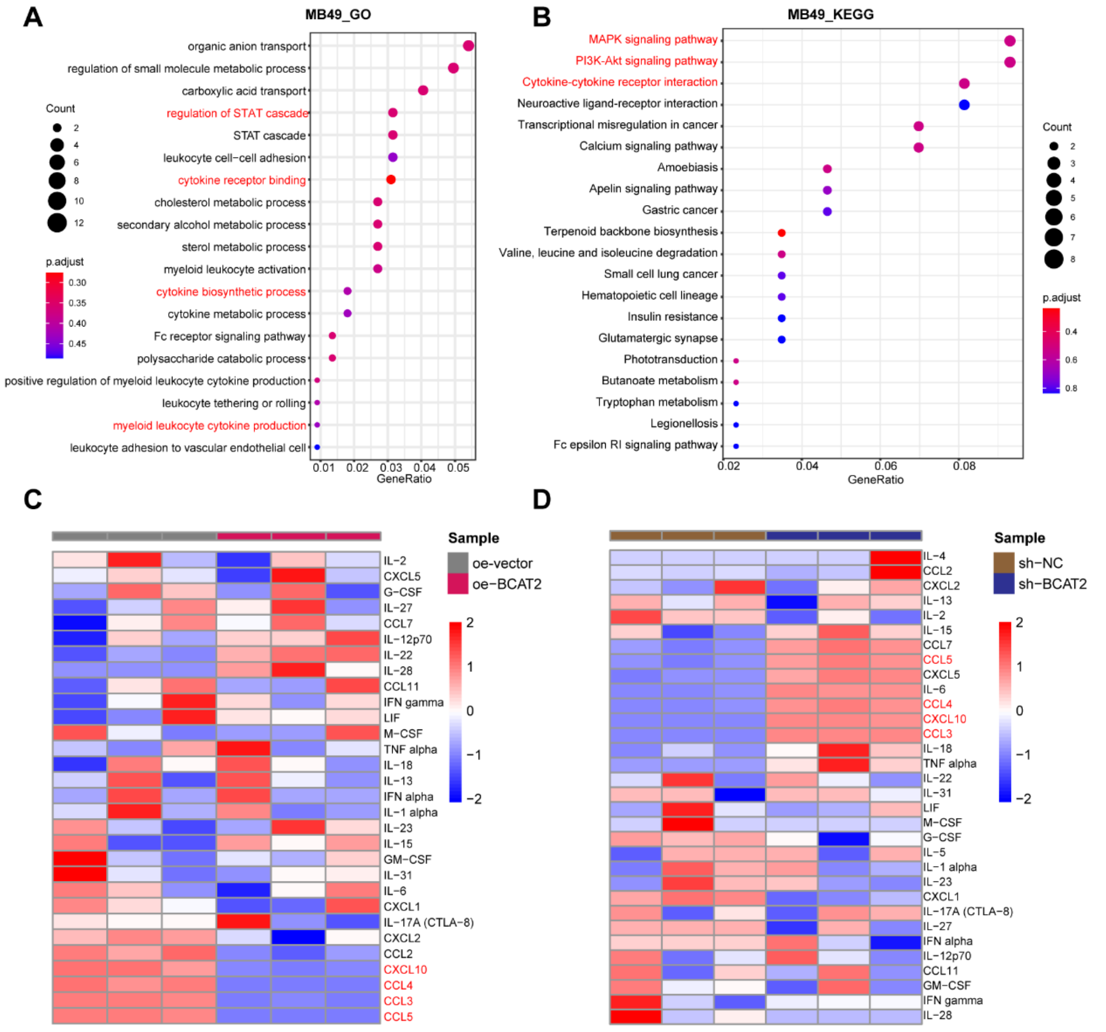

Supplementary Fig 16. Exploring the role of BCAT2 in TME by murine cell line. (A-B) GO and KEGG analyses of DEGs between murine BCAT2 OE and BCAT2 KD cell lines. (C-D) ProcartaPlex multiple immunoassays detected secretion levels of cytokines and chemokines in BCAT2 OE, BCAT2 KD and negative control cell lines.

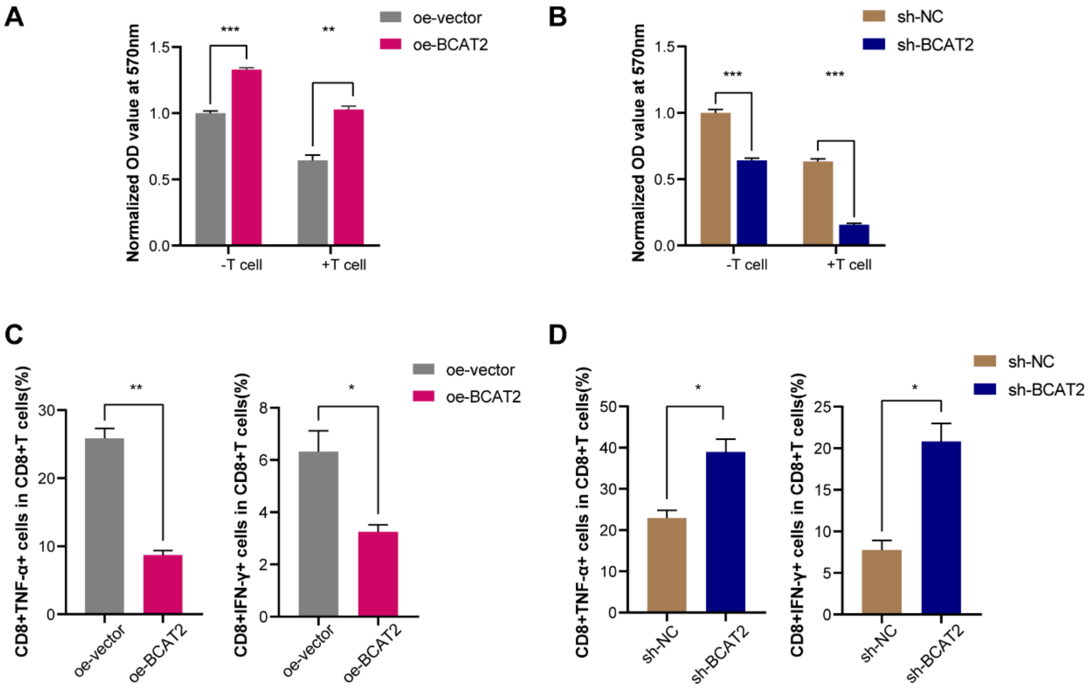

Supplementary Fig 17. The variation of cytotoxicity of T cells and activity of CD8+T cells in different coculture system. (A) Normalized OD value at 570 nm of remained cancer cells (BCAT2-OE and negative control) with and without coculturing with T cells (n=3 per group). **P<0.01, ***P<0.001. (B) Normalized OD value at 570 nm of remained cancer cells (BCAT2-KD and negative control) with and without coculturing with T cells (n=3 per group). ***P<0.001. (C-D) Flow cytometry analysis of proportions of $CD8^{+}TNF\text{-}\alpha^{+}$ T cells and $CD8^{+}IFN\text{-}\gamma^{+}$ T cells in different coculture systems (n=3 per group). *P<0.05, **P<0.01.

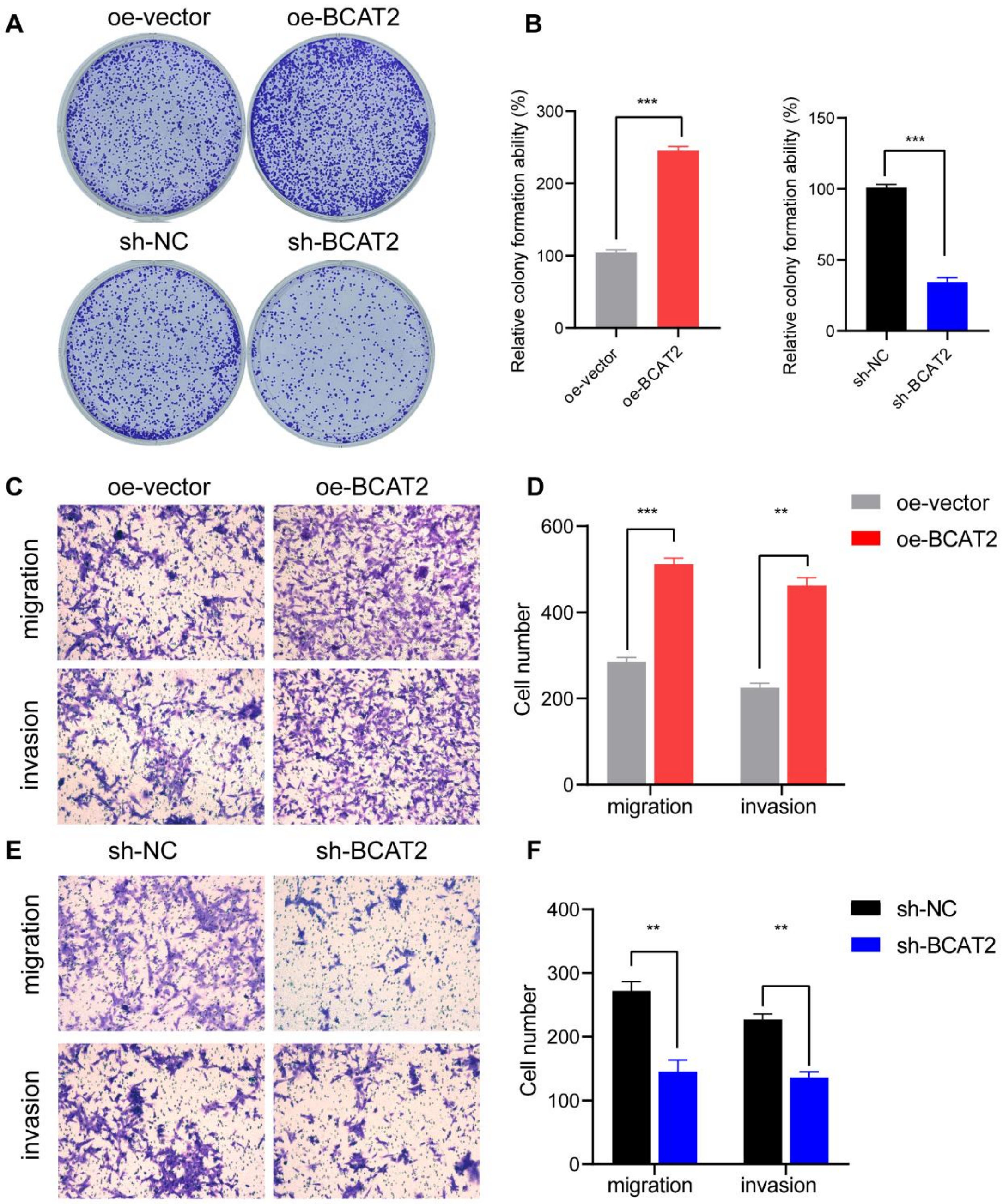

Supplementary Fig 18. The oncogenic role of BCAT2 in vitro. (A-B) Overexpression of BCAT2 significantly enhanced proliferation ability of T24 and knock down of BCAT2 significantly inhibited proliferation ability of T24. ***P< 0.001. (C-F) Overexpression of BCAT2 significantly stimulated migration and invasion abilities of T24 and knock down of BCAT2 significantly impaired migration and invasion abilities of T24. **P< 0.01, ***P< 0.001.

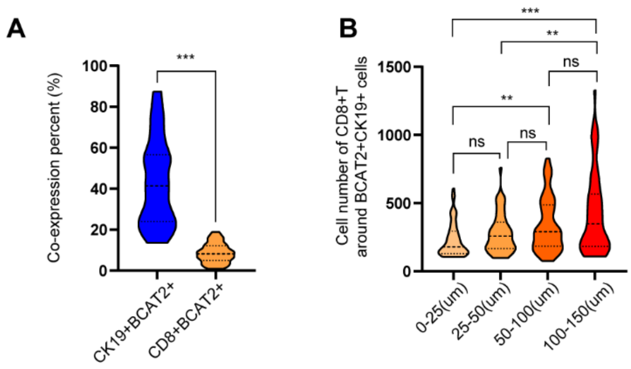

Supplementary Fig 19. Exploring exclusive spatial relationship between BCAT2⁺ tumor cells and CD8⁺T cells on human tissue level. (A) Co-expression proportions of BCAT2⁺CK19⁺ cells and BCAT2⁺CD8⁺ cells in TMA of Xiangya BLCA cohort (n=56). ***P<0.001. (B) Multi-dimensional distance gradient analyses of CD8⁺T cells around BCAT2⁺ tumor cells in TMA of Xiangya BLCA cohort (n=56). **P<0.01, ***P<0.001, ns: no significance.

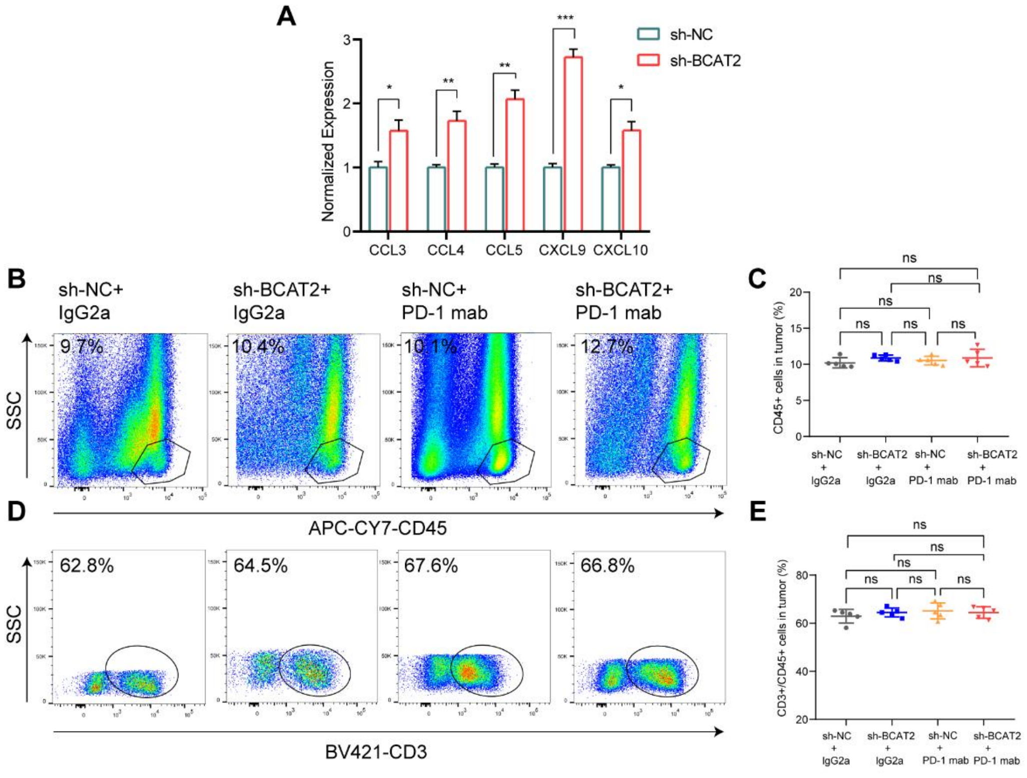

Supplementary Fig 20. The role of BCAT2 on CD8⁺T cell related chemokines, leukocytes and T cells in vivo. (A) Exploring the role of BCAT2 on CD8⁺T cell related chemokines in vivo by qRT-PCR (n=3 per group). *p<0.05, **p<0.01, ***p<0.001. (B-

C) Flow cytometry analysis of proportion of leukocytes ($CD45^+$) (n=5 per group) and (D-E) T cells ($CD45^+CD3^+$) in different therapy regimens (n=5 per group). ns: no significance.

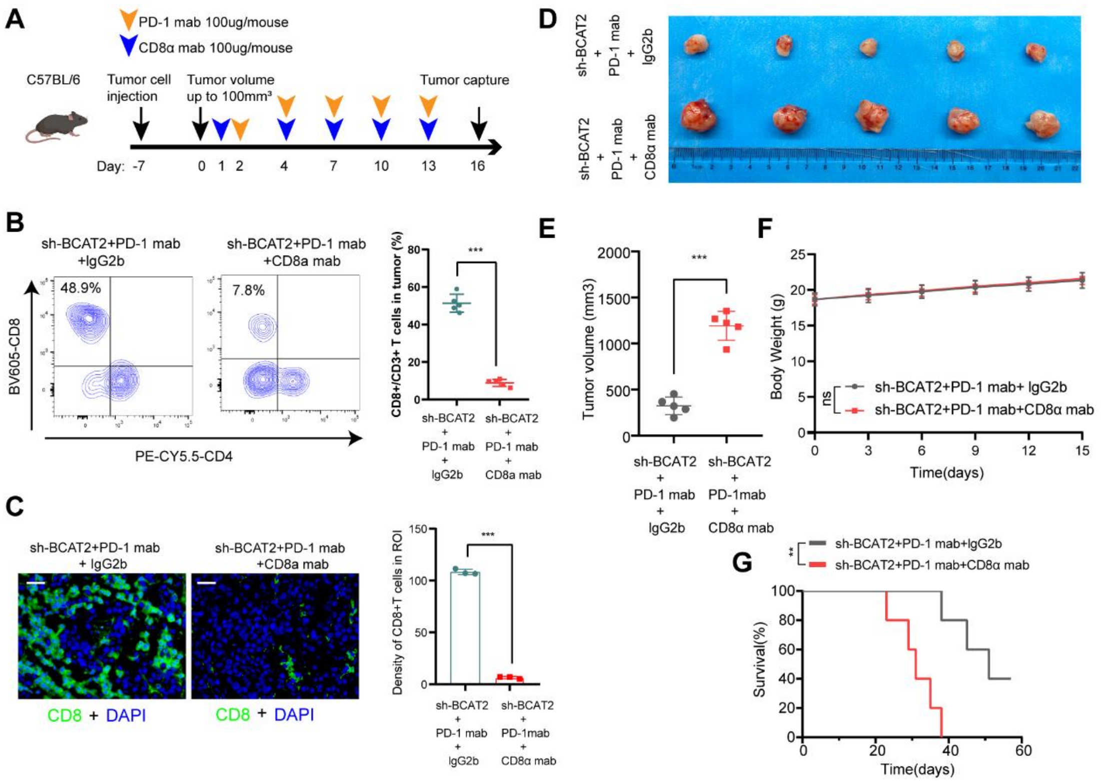

Supplementary Fig 21. $CD8^+$T cells act an indispensable part in synergistic effect of cotreatment. (A) Flow diagram of treatment plan. (B) Validation of successful depletion of $CD8^+$T cells (n=5 per group) by flow cytometry analysis. ***P<0.001. (C) Validation of successful depletion of $CD8^+$T cells by IF (n=3 per group). Scale bar: 20um. ***P<0.001. (D) Subcutaneous tumor model of different therapy regimens. (E) Tumor volume (F) body weight and (G) survival time of tumor-bearing mice (n=5 per group) in different therapy regimens. **P<0.01, ***P<0.001, ns: no significance.

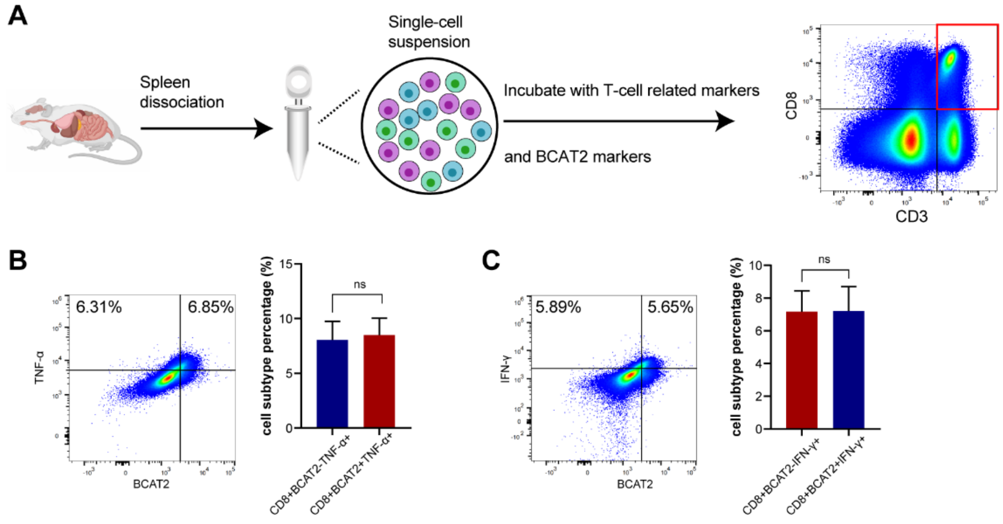

Supplementary Fig 22. The interaction between expression level of BCAT2 on $CD8^{+}$T cell and activity of $CD8^{+}$T cell. (A) Flow diagram of interaction exploration. (B) Proportions of $CD8^{+}$TNF-α^{+} T cells and (C) $CD8^{+}$IFN-γ^{+} T cells between $BCAT2^{+}CD8^{+}$ and $BCAT2^{-}CD8^{+}$ groups (n=3 per group). ns: no significance.

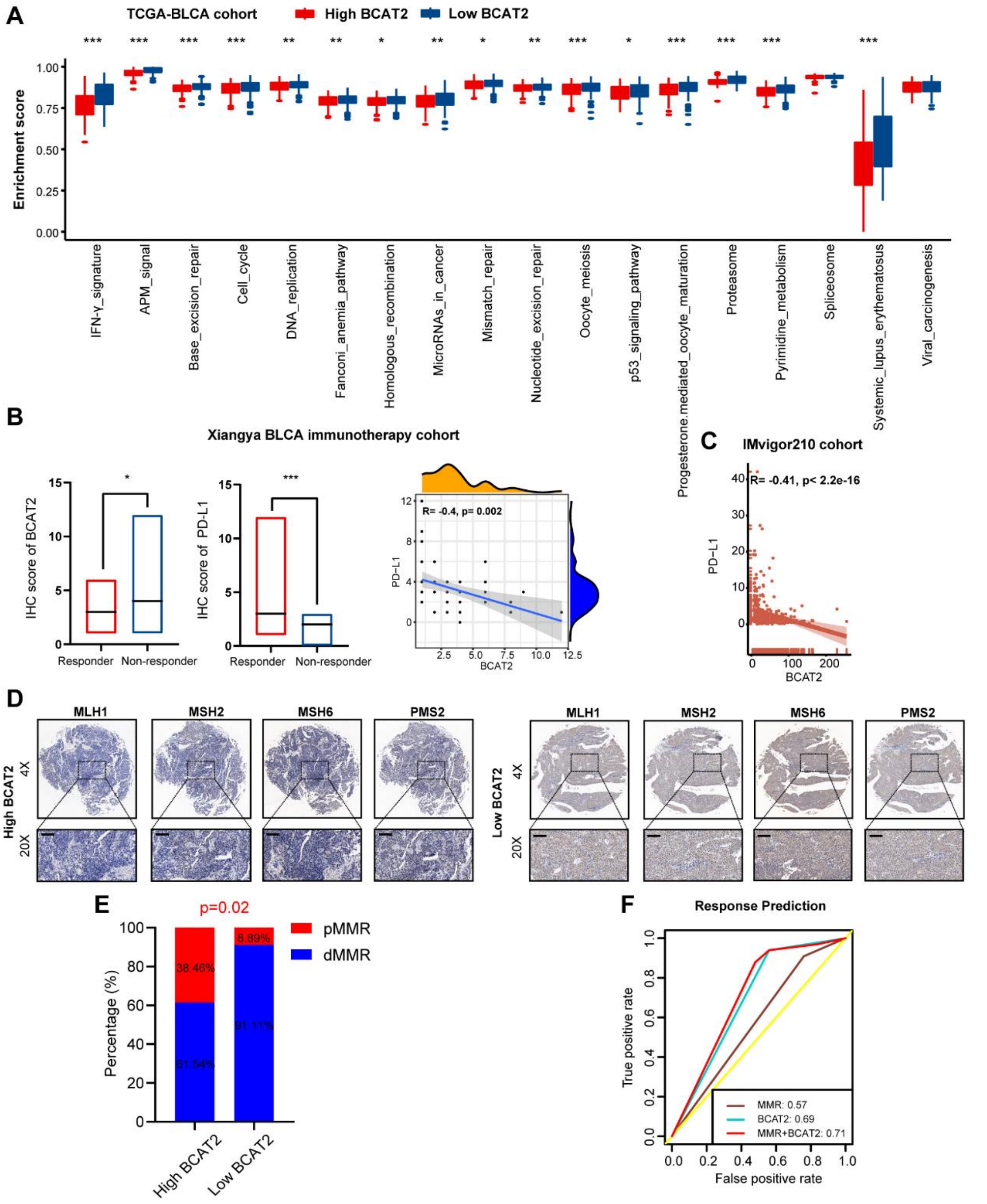

Supplementary Fig 23. The potential of BCAT2 in predicting efficacy of immunotherapy. (A) Enrichment scores of immunotherapy-related pathways between high and low BCAT2 groups in TCGA-BLCA cohort. *P<0.05, **P<0.01, ***P< 0.001. (B) IHC score and correlation of BCAT2 and PD-L1 in Xiangya BLCA immunotherapy cohort. *P<0.05, ***P< 0.001. (C) Correlation between BCAT2 and PD-L1 in IMvigor210 cohort. (D) IHC image (scale bar: 50um) of marker genes of MMR and (E) difference of MMR's status (pMMR and dMMR) between high and low BCAT2 groups in Xiangya BLCA immunotherapy cohort. (F) Prediction accuracy of MMR, BCAT2

and combination index (MMR+BCAT2) in Xiangya BLCA immunotherapy cohort.

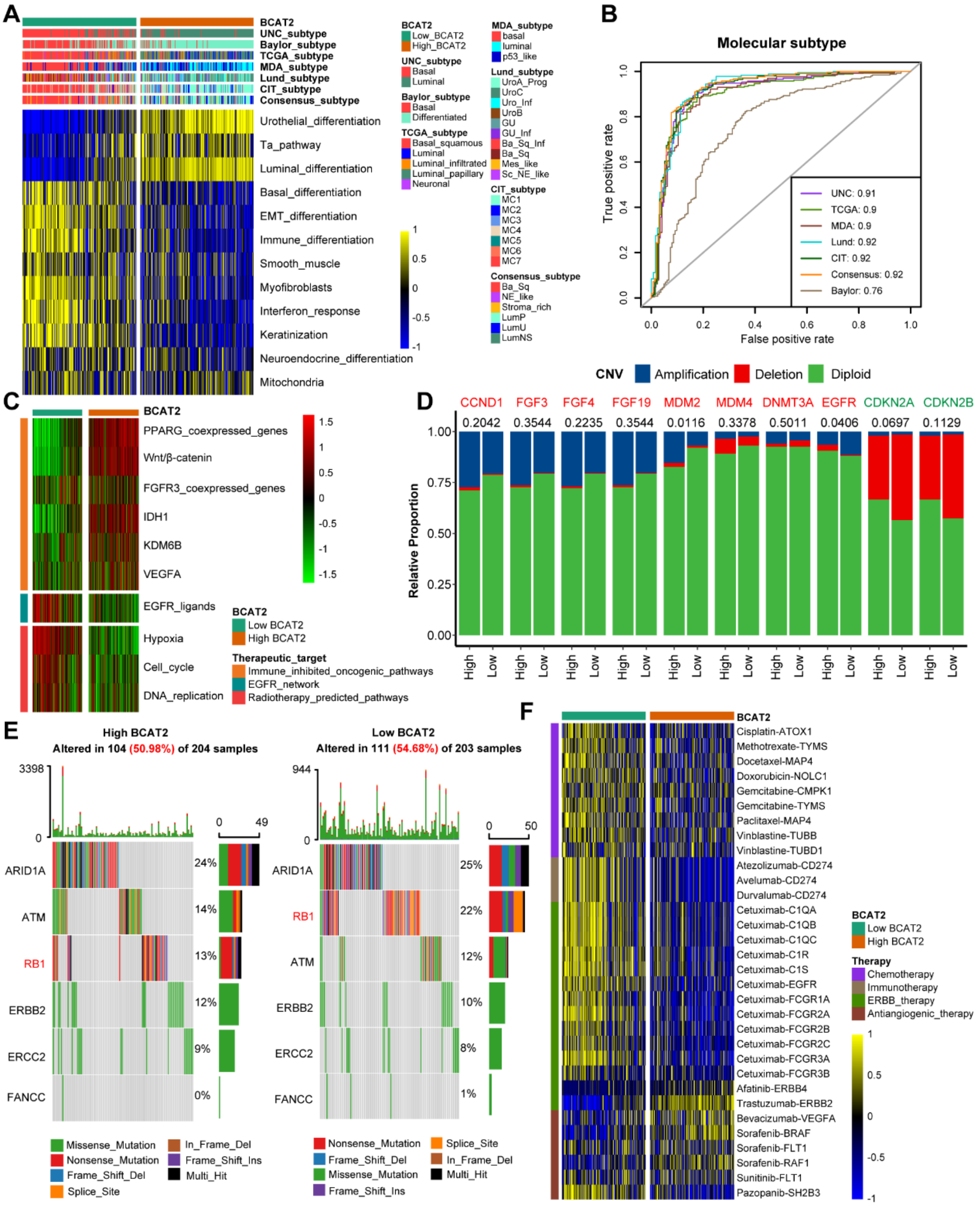

Supplementary Fig 24. The value of BCAT2 in forecasting molecular subtype and guiding precision therapy. (A) The effect of different expression level of BCAT2 on molecular classification systems and activities of basal and luminal related pathways. (B) Prediction accuracy of BCAT2 on molecular subtypes. (C) Activities of EGFR target therapy and radiotherapy related pathways in high and low BCAT2 groups (D) CNV rates of HPD associated biomarkers in different expression of BCAT2. Positive

effector genes of HPD were marked in red and negative effector genes of HPD were marked in green. (E) Mutation rates of NAC related genes in high and low BCAT2 groups. (F) The effect of different expression level of BCAT2 on effector genes of chemotherapy, immunology, ERBB therapy and antiangiogenic therapy.

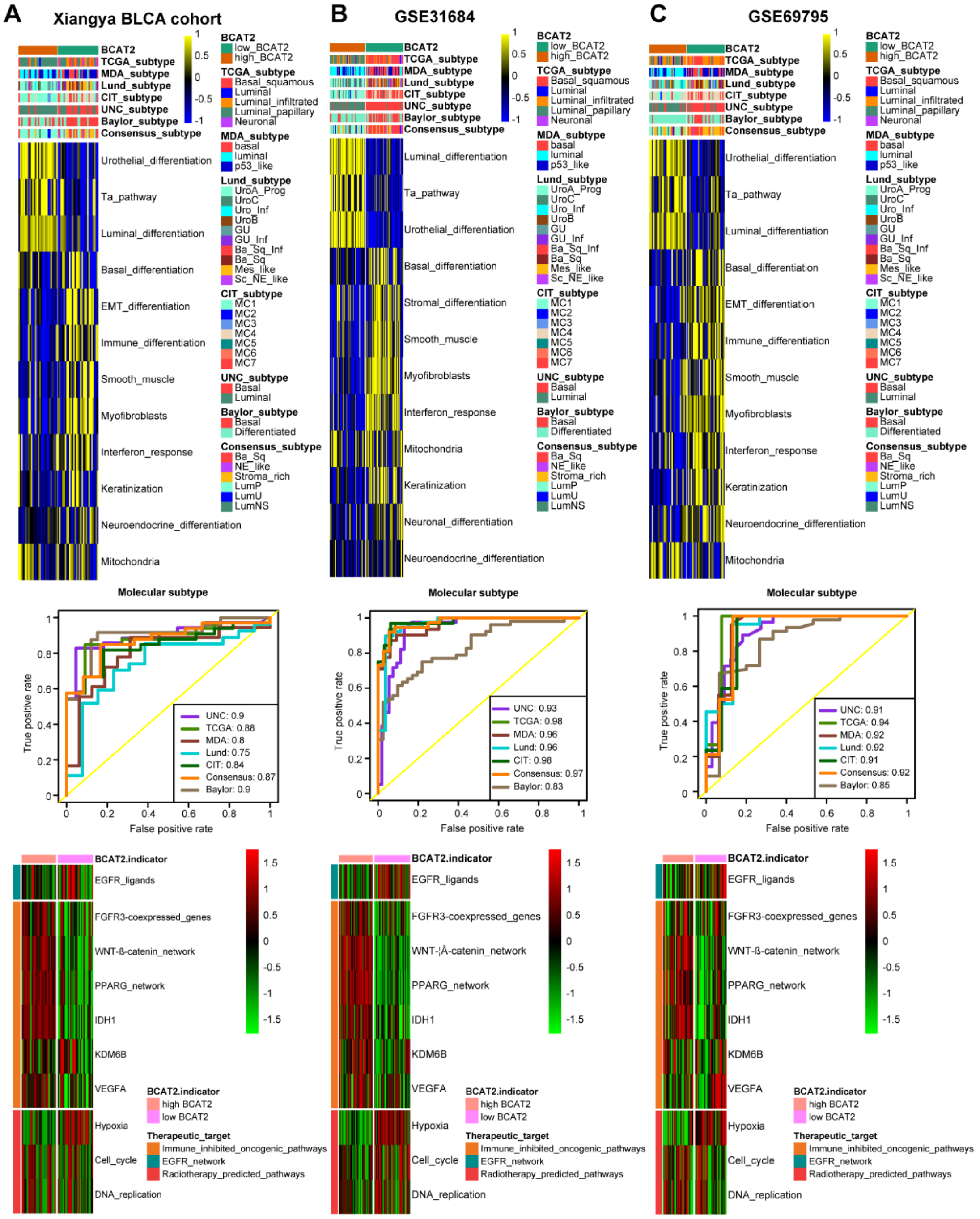

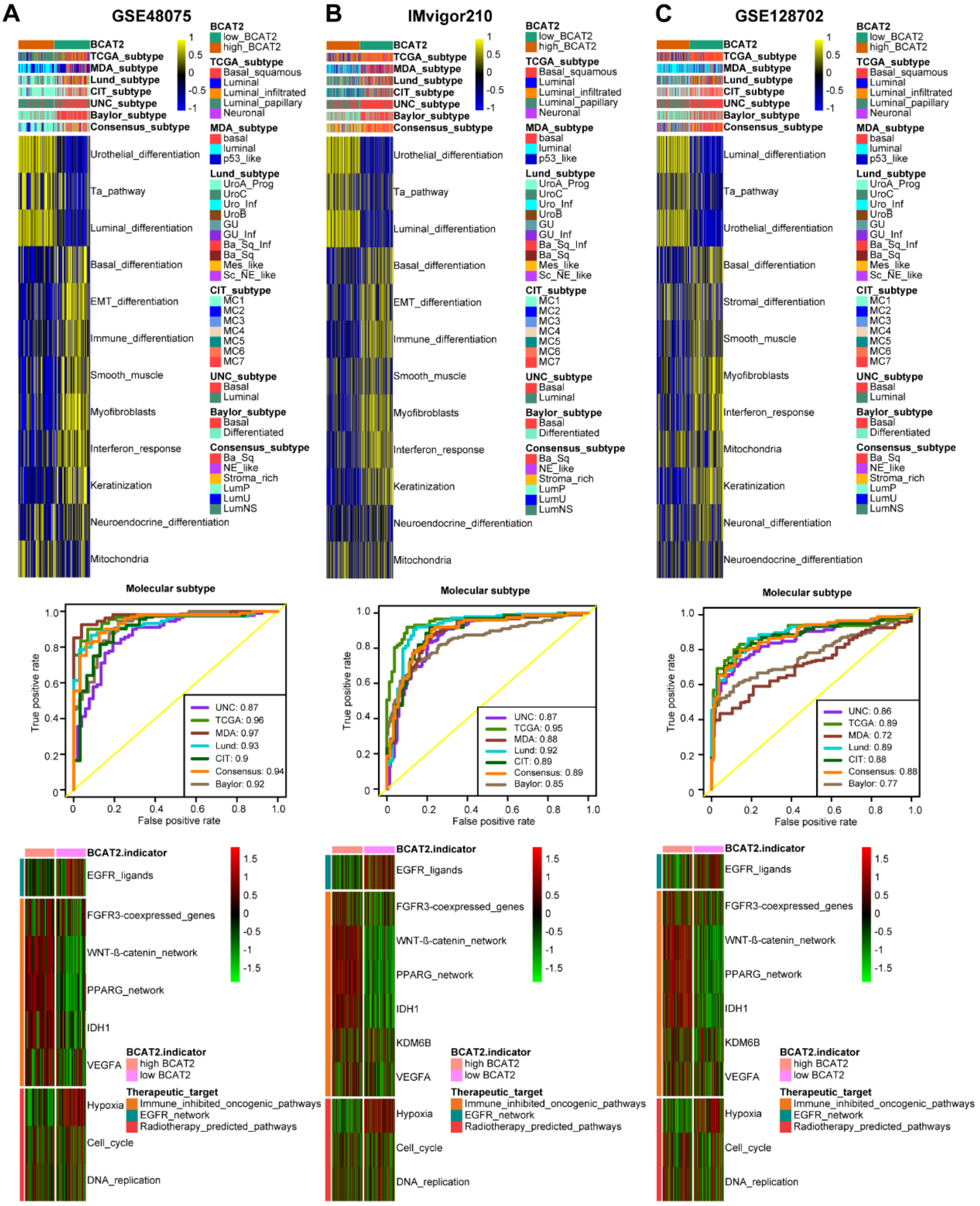
A
GSE48075
B
IMvigor210
C
GSE128702
BCAT2
TCGA_subtype
MDA_subtype
Lund_subtype
CIT_subtype
UNC_subtype
Baylor_subtype
Consensus_subtype
Urothelial_differentiation
Ta_pathway
Luminal_differentiation
Basal_differentiation
EMT_differentiation
Immune_differentiation
Smooth_muscle
Myofibroblasts
Interferon_response
Keratinization
Neuroendocrine_differentiation
Mitochondria
Stromal_differentiation
Neuronal_differentiation
BCAT2
low_BCAT2
high_BCAT2
TCGA_subtype
Basal_squamous
Luminal
Luminal_infiltrated
Luminal_papillary
Neuronal
MDA_subtype
basal
luminal
p53_like
Lund_subtype
UroA_Prog
UroC
Uro_Inf
UroB
GU
GU_Inf
Ba_Sq_Inf
Ba_Sq
Mes_like
Sc_NE_like
CIT_subtype
MC1
MC2
MC3
MC4
MC5
MC6
MC7
UNC_subtype
Basal
Luminal
Baylor_subtype
Basal
Differentiated
Consensus_subtype
Ba_Sq
NE_like
Stroma_rich
LumP
LumU
LumNS
Molecular subtype
True positive rate
False positive rate
UNC: 0.87
TCGA: 0.96
MDA: 0.97
Lund: 0.93
CIT: 0.9
Consensus: 0.94
Baylor: 0.92
UNC: 0.87
TCGA: 0.95
MDA: 0.88
Lund: 0.92
CIT: 0.89
Consensus: 0.89
Baylor: 0.85
UNC: 0.86
TCGA: 0.89
MDA: 0.72
Lund: 0.89
CIT: 0.88
Consensus: 0.88
Baylor: 0.77
BCAT2.indicator
EGFR_ligands
FGFR3-coexpressed_genes
WNT-ß-catenin_network
PPARG_network
IDH1
KDM6B
VEGFA
Hypoxia
Cell_cycle
DNA_replication
BCAT2.indicator
high BCAT2
low BCAT2
Therapeutic_target
Immune_inhibited_oncogenic_pathways
EGFR_network
Radiotherapy_predicted_pathways

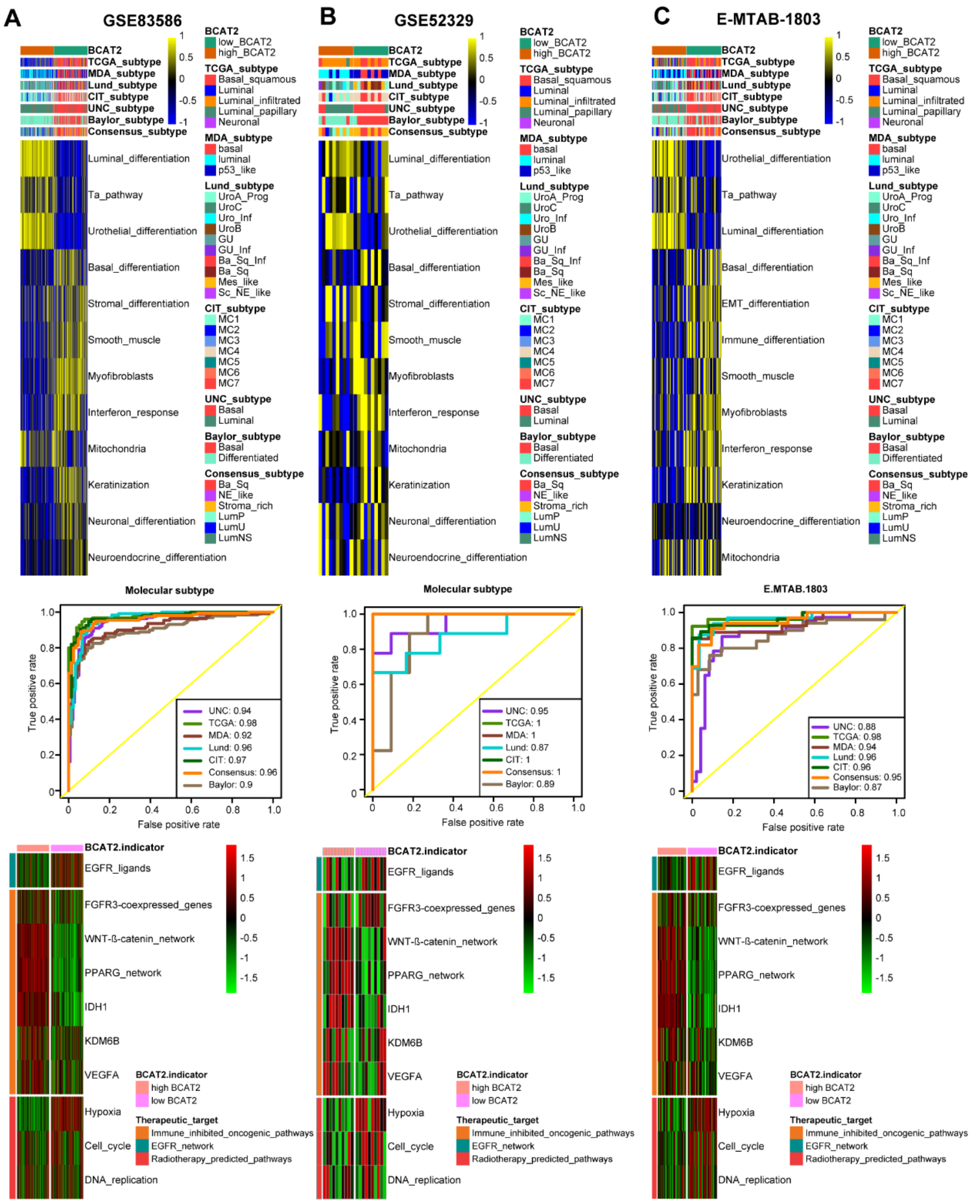

Supplementary Fig 25-27. Validation of prediction accuracy of BCAT2 on molecular classification and treatment sensitivity in nine BLCA cohorts (Xiangya BLCA cohort, GSE31684, GSE69795, GSE48075, IMvigor210, GSE128702, GSE83586, GSE52329 and E-MTAB-1803). (A) The effect of different expression level of BCAT2 on molecular classification systems and activities of basal and luminal related pathways. (B) Prediction accuracy of BCAT2 on molecular subtypes. (C) Activities of EGFR target therapy and radiotherapy related pathways in high and low BCAT2 groups.

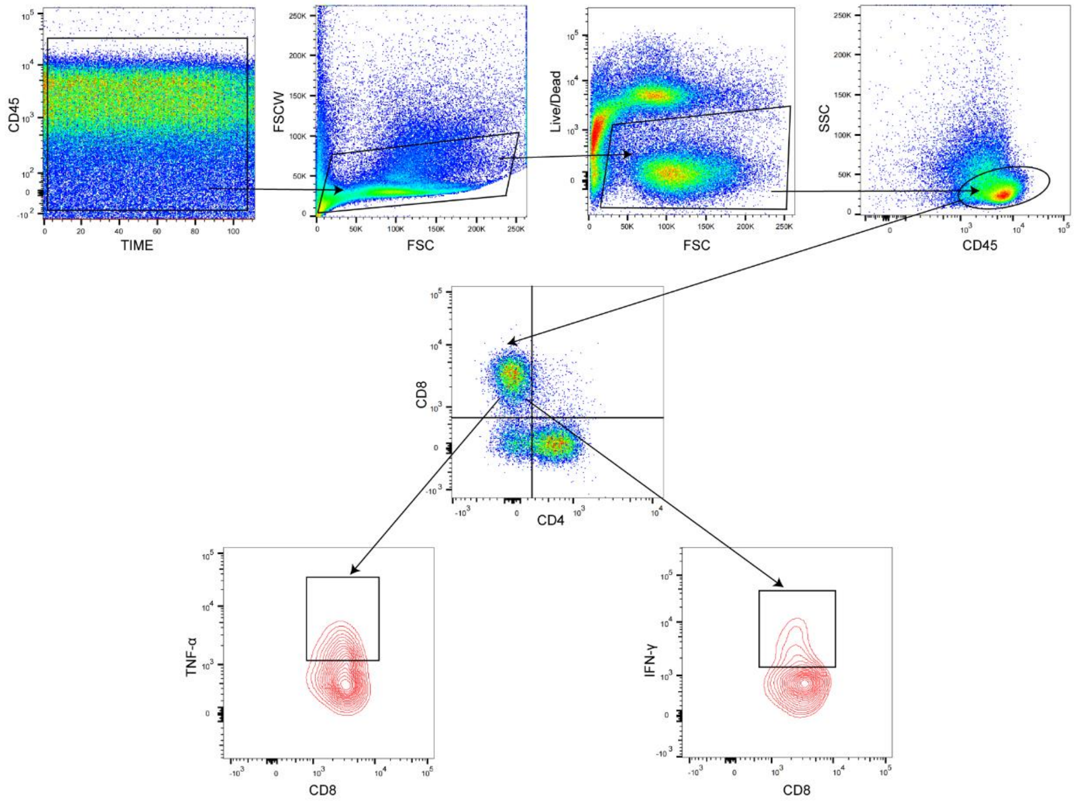

Supplementary Fig 28. Gate strategy of flow cytometry analysis on coculture assay.

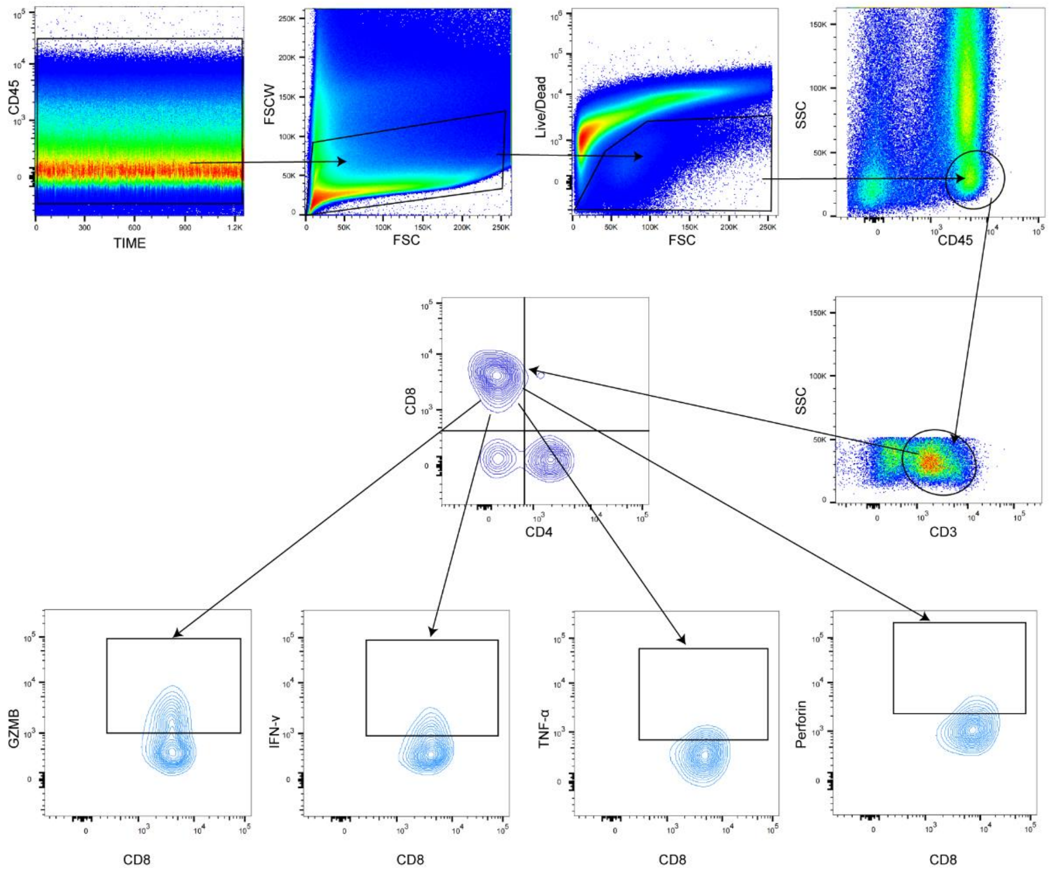

Supplementary Fig 29. Gate strategy of flow cytometry analysis on subcutaneous tumorigenesis model

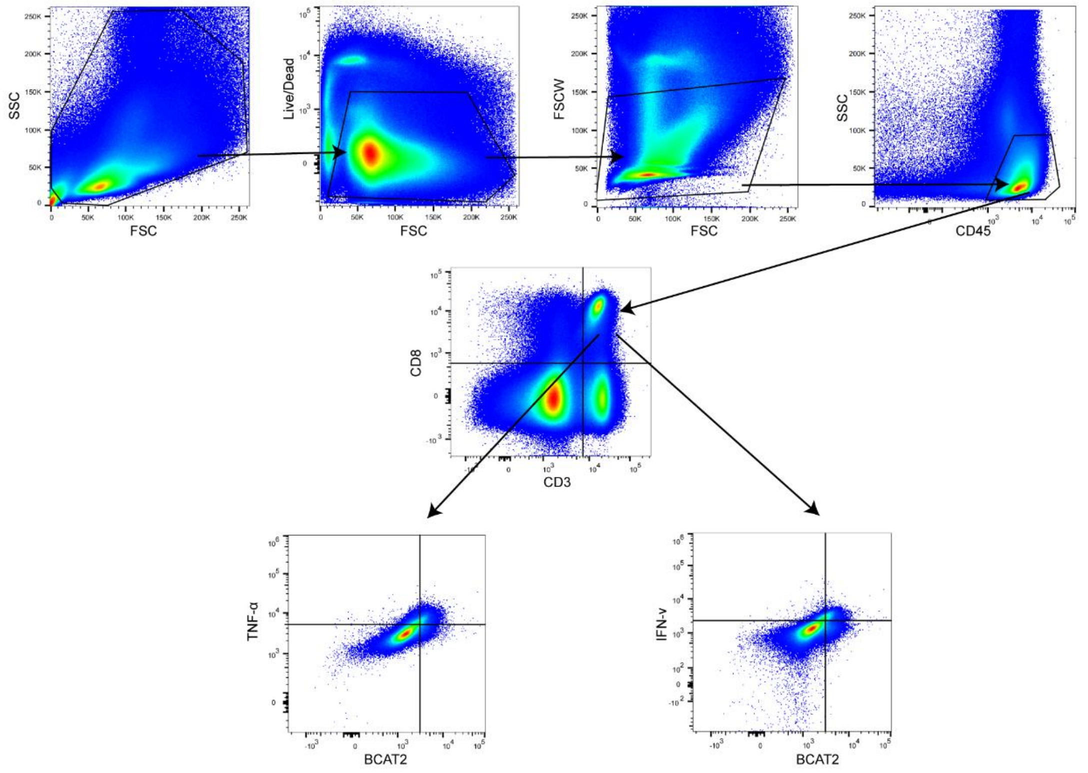

Supplementary Fig 30. Gate strategy of flow cytometry analysis on the interaction between expression level of BCAT2 on CD8^{+}T cell and activity of CD8^{+}T cell.

Supplementary Table 1. The clinicopathological data of Xiangya BLCA cohort.

**ID**	**Age**	**Gender**	**Overall survival (day)**	**Pathological grade**	**Pathological stage**
XYBC-1	58	Female	377	High	T4N0M0
XYBC-2	50	male	1518	High	T2N1M0
XYBC-3	63	male	946	High	T1N0M0
XYBC-4	53	male	1258	High	T1N0M0
XYBC-5	46	male	1213	High	T3bN1M0
XYBC-6	72	Female	1192	Low	T1N0M0
XYBC-7	69	Female	1047	High	T3N0M0
XYBC-8	60	male	970	High	T1N0M0
XYBC-9	57	male	443	High	T3N0M0

XYBC-10	70	male	975	Low	T1N0M0
XYBC-11	54	male	877	High	T1N0M0
XYBC-12	61	male	911	High	T1N0M0
XYBC-13	67	male	914	High	T2N0M0
XYBC-14	62	male	662	High	T3N0M0
XYBC-15	55	male	905	High	T1N0M0
XYBC-16	66	male	423	High	T2aN0M0
XYBC-17	66	male	851	High	T1N0M0
XYBC-18	45	male	876	High	T4N0M0
XYBC-19	71	male	857	High	T2N0M0
XYBC-20	74	male	807	High	T3bN1M0
XYBC-21	63	male	797	High	T4N0M0
XYBC-22	62	Female	766	High	T2N0M0
XYBC-23	55	male	781	Low	T1N0M0
XYBC-24	59	male	881	High	T4N0M0
XYBC-25	70	male	797	High	T4N1M0
XYBC-26	54	male	736	High	T1N0M0
XYBC-27	69	male	736	High	T2N0M0
XYBC-28	73	male	859	High	T2bN0M0
XYBC-29	75	male	659	High	T1N0M0
XYBC-30	77	male	671	High	T3N1M0
XYBC-31	50	Female	689	High	T3N0M0
XYBC-32	66	male	645	Low	T1N0M0
XYBC-33	77	Female	653	High	T3N0M0
XYBC-34	60	male	646	High	T1N0M0
XYBC-35	70	male	622	High	T2N0M0
XYBC-36	60	male	147	High	T3N0M0
XYBC-37	80	male	972	High	T3N0M0
XYBC-38	66	male	606	High	T2bN0M0

XYBC-39	77	male	592	Low	T1N0M0
XYBC-40	83	male	565	Low	T1N0M0
XYBC-41	73	male	592	Low	T1N0M0
XYBC-42	70	male	549	High	T2N0M0
XYBC-43	33	male	556	Low	T1N0M0
XYBC-44	45	male	526	High	T4N0M0
XYBC-45	56	male	523	High	T4N0M1
XYBC-46	64	male	534	High	T2N0M1
XYBC-47	65	Female	239	High	T3aN1M1
XYBC-48	78	male	299	High	T2bN0M0
XYBC-49	67	Female	725	Low	T1N0M0
XYBC-50	71	male	159	High	T4N0M1
XYBC-51	62	male	235	Low	T1N0M0
XYBC-52	65	male	240	High	T1N0M0
XYBC-53	51	male	230	High	T1N0M0
XYBC-54	71	male	204	Low	T0N0M0
XYBC-55	66	Female	242	High	T4N0M1
XYBC-56	64	Female	226	Low	T2N0M0

Supplementary Table 2. The clinicopathological data of Xiangya BLCA immunotherapy cohort.

**ID**	**Response**	**Age**	**Gender**	**Surgical Treatment**
XYBIC-1	CR	49	male	TURBT
XYBIC-2	CR	71	male	Radical cystectomy
XYBIC-3	CR	49	male	TURBT
XYBIC-4	CR	66	male	Radical cystectomy
XYBIC-5	CR	71	male	TURBT
XYBIC-6	CR	46	male	TURBT

XYBIC-7	CR	56	male	TURBT
XYBIC-8	CR	57	male	TURBT
XYBIC-9	CR	63	male	Radical cystectomy
XYBIC-10	CR	69	male	TURBT
XYBIC-11	CR	56	male	Radical cystectomy
XYBIC-12	CR	73	male	Radical cystectomy
XYBIC-13	CR	62	male	Radical cystectomy
XYBIC-14	CR	63	male	Radical cystectomy
XYBIC-15	CR	47	female	TURBT
XYBIC-16	CR	50	male	TURBT
XYBIC-17	CR	59	male	Radical cystectomy
XYBIC-18	PR	63	male	Radical cystectomy
XYBIC-19	PR	69	male	Radical cystectomy
XYBIC-20	PR	57	male	Radical cystectomy
XYBIC-21	PR	71	male	Radical cystectomy
XYBIC-22	PR	73	male	Radical cystectomy
XYBIC-23	PR	52	male	Radical cystectomy
XYBIC-24	PR	57	female	Radical cystectomy
XYBIC-25	PR	57	female	Radical cystectomy
XYBIC-26	PR	63	male	Radical cystectomy
XYBIC-27	PR	58	male	Radical cystectomy
XYBIC-28	PR	68	male	Radical cystectomy
XYBIC-29	PR	66	male	Radical cystectomy
XYBIC-30	PR	54	male	Radical cystectomy
XYBIC-31	PR	68	female	Radical cystectomy
XYBIC-32	PR	72	female	Radical cystectomy
XYBIC-33	PR	79	male	Radical cystectomy
XYBIC-34	SD	67	male	Radical cystectomy
XYBIC-35	SD	72	male	Radical cystectomy

XYBIC-36	SD	81	male	Radical cystectomy
XYBIC-37	SD	71	male	Radical cystectomy
XYBIC-38	SD	56	male	Radical cystectomy
XYBIC-39	SD	67	female	Radical cystectomy
XYBIC-40	SD	74	female	Radical cystectomy
XYBIC-41	SD	32	male	Radical cystectomy
XYBIC-42	SD	73	male	Radical cystectomy
XYBIC-43	SD	62	male	Radical cystectomy
XYBIC-44	SD	60	male	Radical cystectomy
XYBIC-45	SD	80	male	Radical cystectomy
XYBIC-46	SD	63	male	Radical cystectomy
XYBIC-47	SD	58	male	Radical cystectomy
XYBIC-48	SD	49	male	Radical cystectomy
XYBIC-49	SD	61	female	Radical cystectomy
XYBIC-50	SD	74	male	Radical cystectomy
XYBIC-51	PD	69	male	Radical cystectomy
XYBIC-52	PD	57	male	Radical cystectomy
XYBIC-53	PD	46	male	Radical cystectomy
XYBIC-54	PD	57	female	Radical cystectomy
XYBIC-55	PD	63	female	Radical cystectomy
XYBIC-56	PD	68	male	Radical cystectomy
XYBIC-57	PD	68	male	Radical cystectomy
XYBIC-58	PD	71	male	Radical cystectomy

Supplementary Table 3. Target sequences of shRNA

Target sequences of shRNA for human species	
sh-BCAT2-1	gtGCACCGAATCCTGTACAAA
sh-BCAT2-2	acTACAAGTTAGGTGGGAATT
sh-BCAT2-3	tgAAGTGCAATACGAAATAAA

Target sequences of shRNA for murine species	
sh-BCAT2-1	ccACATACCTACCATGGAGAA
sh-BCAT2-2	caAGGTCACTATGAAGGAATT
sh-BCAT2-3	ccCTTCCAGAACCTCACGCTA

Supplementary Table 4. Primer sequences of qRT-PCR

Species: human

	**Forward**	**Reverse**
CCL3	CATGGCTCTCTGCAACCAGTTCTC	CTGGCTGCTCGTCTCAAAGTAGTC
CCL4	TCTGCGTGACTGTCCTGTCTCTC	TCTACCACAAAGTTGCGAGGAAGC
CCL5	CAGCAGTCGTCCACAGGTCAAG	TTTCTTCTCTGGGTTGGCACACAC
CXCL9	AAGACCTTAAACAATTTGCCCC	TGCTGAATCTGGGTTTAGACAT
CXCL10	CTCTCTCTAGAACTGTACGCTG	ATTCAGACATCTCTTCTCACCC

Species: mouse

	**Forward**	**Reverse**
CCL3	CCCAGCCAGGTGTCATTT	AGTTCCAGGTCAGTGATGTATTC
CCL4	CCACTTCCTGCTGTTTCTCTTA	GCAAAGACTGCTGGTCTCATA
CCL5	GCCCACGTCAAGGAGTATTT	CCCACTTCTTCTCTGGGTTG
CXCL9	AGGCACGATCCACTACAAATC	GCAGGTTTGATCTCCGTTCT
CXCL10	GCTGCAACTGCATCCATATC	CGTGGCAATGATCTCAACAC
